# Novel Evolutionarily Conserved Oncogene *COA4* is Driven by *KRAS* Mutant and Promotes Cancer Metastasis Through Dual Mitochondrial Metabolism‐Dependent and ‐Independent Mechanisms

**DOI:** 10.1002/advs.202507533

**Published:** 2025-09-11

**Authors:** Xingzhao Ji, Weiying Zhang, Fuyuan Xue, Jiazhen Zeng, Qinghua Zhao, Xiaoming Sun, Jian Sun, Heng Zhou, Quanlin Xu, Guoyuan Ma, Shengnan Sun, Ying Wang, Qian Mu, Yi Liu, Qiang Wan

**Affiliations:** ^1^ Shandong Provincial Key Medical and Health Laboratory of cell metabolism Central Hospital Affiliated to Shandong First Medical University Jinan Shandong 250021 China; ^2^ Department of Pulmonary and Critical Care Medicine Shandong Provincial Hospital Affiliated to Shandong First Medical University Jinan Shandong 250021 China; ^3^ Medical Science and Technology Innovation Center Shandong First Medical University & Shandong Academy of Medical Sciences Jinan Shandong 250021 China; ^4^ Department of Endocrinology and Metabolism Second Affiliated Hospital of Harbin Medical University Harbin Heilongjiang 150000 China; ^5^ Department of Thoracic Surgery Department Shandong Provincial Hospital Affiliated to Shandong First Medical University Jinan Shandong 250021 China

**Keywords:** COA4, cytochrome c oxidase, KRAS, metastasis, OXPHOS, Saccharomyces cerevisiae

## Abstract

Metastasis remains the leading cause of cancer‐related mortality, yet effective interventions against *KRAS* ^G12C/D^ driven lung adenocarcinoma metastasis ared limited. In this study, using *KRAS* ^G12D/–^;*TP53* ^–/–^;*COA4* ^–/–^ transgenic mice, clinical specimens, organoid models, RNA sequencing, xenograft assays, and Seahorse metabolic profiling are employed to identify *COA4* as an evolutionarily conserved regulator of cytochrome c oxidase (COX) and activator of CDC42. *COA4* is found to be highly overexpressed in *KRAS* mutant tumors, correlating with increased metastatic burden and poor prognosis. Notably, *COA4* deficiency markedly reduces lymph node metastasis. Mechanistically, *KRAS* ^G12C/D^ upregulates *COA4* via PI3K signaling and E2F1‐mediated transcriptional activation. Functionally, *COA4* overexpression enhances transendothelial migration, extravasation, metastatic colonization, and organoid formation in vitro and in vivo, while its knockdown reverses *KRAS*‐driven metastasis without affecting proliferation. Subcellular fractionation reveals that mitochondrial COA4 augments COX activity to drive oxidative phosphorylation, while cytosolic COA4 binds and activates CDC42 to regulate pseudopodia formation. Pharmacological blockade of COX, oxidative phosphorylation, or CDC42 effectively suppressed COA4‐driven metastasis, with combination treatments yielding synergistic inhibition. Remarkably, the *Saccharomyces cerevisiae COA4* ortholog recapitulates these dual functions, underscoring their evolutionary conservation. These findings establish *COA4* as a critical *KRAS* ^G12C/D^ effector governing metastasis through dual COX‐CDC42 modulation, highlighting its therapeutic potential for *KRAS* ^G12C/D^‐driven malignancies.

## Graphical abstract

In KRAS‐mutant tumors, KRAS transcriptionally upregulates COA4 via the PI3K/AKT/E2F1 axis, promoting metastasis. Mechanistically, COA4 enhances tumor cell migration through dual pathways: within mitochondria, it boosts oxidative phosphorylation; in the cytoplasm, it binds and activates CDC42 to stimulate pseudopodia formation. Thus, COA4 represents a potential therapeutic target for inhibiting metastasis in KRAS‐driven cancers.

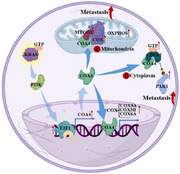

## 1 Introduction

Lung cancer is the leading cause of cancer‐related death worldwide, with non‐small cell lung cancer (NSCLC) representing the predominant histological subtype.^[^ ^1^ ^]^ Lung adenocarcinoma (LUAD), the most common form of NSCLC,^[^ ^2^ ^]^ is associated with high morbidity and mortality due to its propensity for rapid metastasis to lymph nodes, the contralateral lung, and multiple distant organs.^[^ ^3^ ^]^ Notably, nearly half of patients diagnosed at early stages (I or II) eventually develop metastases months to years after primary tumor treatment.^[^ ^4^ ^]^ Metastasis is the principal cause of cancer‐related mortality, including in LUAD.^[^ ^5^ ^]^ Despite significant advances in therapeutic strategies over recent decades, the long‐term prognosis for metastatic LUAD remains poor, with a median survival of merely 5 months, and more than half of patients succumb within one year.^[^ ^4^ ^]^ Furthermore, metastatic LUAD patients typically exhibit poor responsiveness to current treatments and a high rate of post‐treatment relapse.^[^ ^3^ ^]^ Thus, there is an urgent need to elucidate the molecular mechanisms underlying LUAD metastasis and to identify novel biomarkers and therapeutic targets for these patients.

LUAD frequently harbors oncogenic driver mutations in genes such as *EGFR*, *ALK*, *ROS1*, and *RET*, for which targeted therapies have been developed.^[^ ^6^ ^]^ However, effective targeted drugs for gain‐of‐function mutations in Kirsten rat sarcoma viral oncogene homolog (*KRAS*)—present in nearly 30% of LUAD patients—remain scarce.^[^ ^7^ ^]^ Oncogenic *KRAS* mutations impair GTP hydrolysis, resulting in constitutively active, GTP‐bound proteins that continuously recruit and activate downstream effector pathways, including the RAF‐MEK‐ERK (MAPK), PI3K‐AKT, and RALGDS‐RAL cascades, ultimately leading to aggressive malignancies.^[^ ^8^ ^]^ Although clinical trials have shown that LUAD patients harboring the *KRAS* ^G12C^ mutation benefit from the *KRAS* ^G12C^‐specific inhibitor AMG 510, resistance frequently develops, and other *KRAS* mutations remain largely undruggable.^[^ ^9^ ^]^ Pharmacologic blockade of downstream KRAS effectors has thus emerged as an alternative strategy, and emerging evidence suggests that *KRAS*‐mutant cancers are particularly vulnerable to inhibition of aberrant metabolic pathways.^[^ ^10^ ^]^ Although accumulating evidence has established the association between *KRAS* activation and metabolic reprogramming in tumor cells, the molecular mechanisms underlying *KRAS*‐mediated metabolic rewiring in LUAD remain incompletely elucidated.^[^ ^11^ ^]^ Therefore, further investigation into the downstream mechanisms of *KRAS* signaling—especially those related to mitochondrial metabolism—is critical for the development of effective therapies for *KRAS*‐driven LUAD.

Cancer cells exhibit a fundamentally altered metabolic profile compared to normal tissues, characterized by a shift from mitochondrial ATP synthesis via oxidative phosphorylation (OXPHOS) towards high rates of glycolysis—a phenomenon long recognized as the “Warburg effect.”^[^ ^12^ ^]^ This glycolytic switch was once thought to be a principal driver of tumor progression; however, recent studies have challenged this notion by demonstrating that OXPHOS is elevated rather than suppressed in LUAD cells.^[^ ^13^ ^]^ For example, Martínez‐Reyes et al., using intraoperative ^13^C‐glucose infusions in vivo, showed that human NSCLC tumors display enhanced glucose oxidation relative to adjacent benign lung tissue. Moreover, in a murine lung cancer model, they demonstrated that mitochondrial activity is critical for tumor initiation and progression.^[^ ^14^ ^]^ Importantly, several studies have established that functional mitochondrial respiration is essential for tumor metastasis, with metastatic tumors exhibiting high OXPHOS levels.^[^ ^13^, ^15^ ^]^ These findings underscore the importance of further characterizing metabolic reprogramming in LUAD, clarifying the role of OXPHOS in metastasis, and identifying potential therapeutic strategies targeting this metabolic shift.

Cytochrome c oxidase assembly factor 4 (COA4) was initially identified in *Saccharomyces cerevisiae* (*S. cerevisiae*) as a novel member of the cytochrome c oxidase (COX) assembly factor family, characterized by a dual CX9C motif, and predominantly localized in the mitochondrial intermembrane space.^[^ ^16^ ^]^ COA4 is closely associated with the mitochondrial copper delivery pathway at the CuB site of the COX1 subunit, and its absence in yeast leads to reduced COX activity, highlighting its role in maintaining COX assembly and respiratory chain biogenesis.^[^ ^17^ ^]^ Luo et al. identified *COA4* as a potential prognostic marker in osteosarcoma through bioinformatic analysis, while Krobthong et al. reported COA4 as a putative cancer‐promoting protein.^[^ ^18^ ^]^ Moreover, related proteins such as COX6B2 have been reported to drive metastasis in pancreatic ductal adenocarcinoma (PDAC) by enhancing OXPHOS.^[^ ^15a^ ^]^ However, the biological function of COA4 in mitochondrial OXPHOS of mammalian cells has not been demonstrated. Besides, it has been reported that RAS activation could upregulate COX activity and mitochondrial respiration in LUAD cells.^[^ ^19^ ^]^ Whether *COA4* is regulated by KRAS in cancers remains unknown.

In this study, we demonstrate that *COA4* is an evolutionarily conserved gene that participates in the regulation of mitochondrial COX and activation of CDC42 in mammalian cells. We show that *KRAS* ^G12C/D^ drives *COA4* expression via the transcription factor E2F1, thereby promoting metastasis. Mechanistically, COA4 enhances tumor cell metastasis by upregulating mitochondrial OXPHOS and by interacting with CDC42 to activate its activity. Notably, both combined and individual treatments with OXPHOS or COX inhibitors, as well as CDC42 inhibitors, significantly suppressed COA4 overexpression–induced metastasis in LUAD. Finally, we found that *COA4* derived from *S. cerevisiae* is functionally conserved. Taken together, our findings highlight COA4 as a critical target for the treatment of KRAS mutant‐driven cancer metastasis.

## 2 Results

### 2.1 *COA4* Manipulates Mitochondrial Metabolism via Regulating Cytochrome C Oxidase

**Figure 1:**
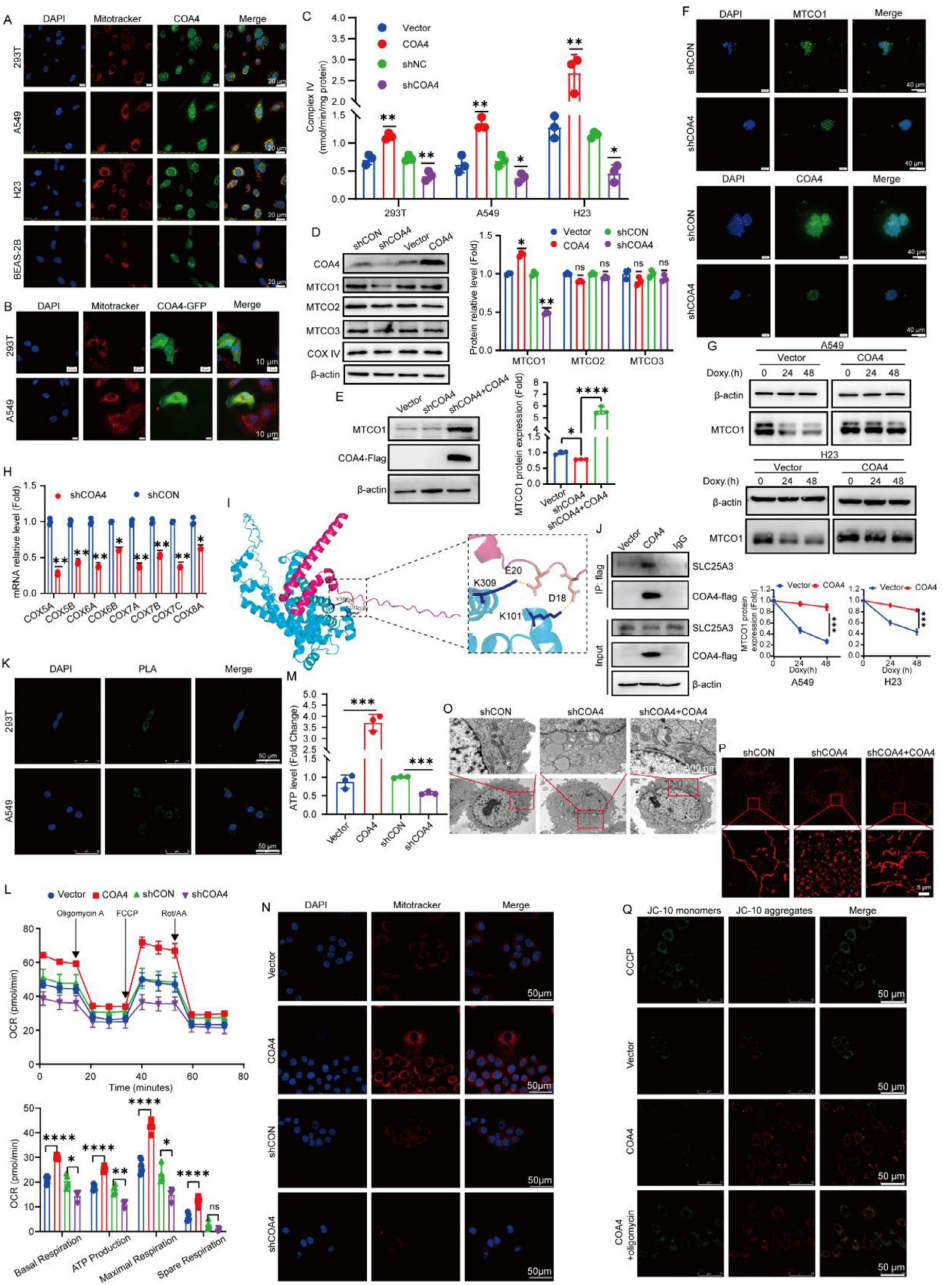
*COA4* manipulates mitochondrial metabolism via regulating cytochrome c oxidase. A) IF analysis of endogenous COA4 in 293T, A549, H23, and BEAS‐2B cells, with nuclei and mitochondria. Scale bar: 20 µm. (*n* = 3). B) IF analysis of exogenous COA4‐GFP in 293T and A549 cells, with nuclei and mitochondria. Scale bar: 10 µm. (*n* = 3). C) Complex IV activity measured in 293T, A549, and H23 cells following *COA4* knockdown or overexpression. (*n* = 3; *p < 0.05; **p < 0.01). D) Western blot analysis showing protein levels of mitochondrially encoded complex IV subunits in A549 cells with *COA4* knockdown or overexpression. (*n* = 3; ns, not significant; *p < 0.05; **p < 0.01). E) Rescue experiment in *COA4*‐knockdown A549 cells demonstrating restoration of MTCO1 protein levels upon re‐expression of *COA4*. (n = 3; *p < 0.05; ****p < 0.0001). F) IF analysis of MTCO1 and COA4 in LUAD patient‐derived organoids (PDOs) following COA4 knockdown. Scale bar: 40 µm. G) Western blot analysis of MTCO1 in *COA4*‐overexpressing A549 and H23 cells treated with doxycycline. Treated with mitochondrial ribosome inhibitor doxycycline (1 µg/mL) for 0, 24, and 48 h (*n* = 3; ***p < 0.001). H) Quantitative RT‐PCR analysis of mRNA levels for nuclear‐encoded complex IV subunits in A549 cells with *COA4* knockdown or overexpression. (*n* = 3; *p < 0.05; **p < 0.01). I) AlphaFold 3 molecular docking model illustrating the predicted interaction between COA4 and SLC25A3. J) Co‐immunoprecipitation (Co‐IP) of *COA4*‐Flag in A549 cells, confirming the interaction between Coa4 and SLC25A3. (*n* = 3). K) Duolink PLA visualizing the COA4‐SLC25A3 interaction in A549 and 293T cells. Scale bar: 40 µm. (*n* = 3). L) Seahorse XFe96 analysis of oxygen consumption rate (OCR) in A549 cells with COA4 knockdown or overexpression, assessing OXPHOS parameters including basal respiration, maximal respiration, ATP production, and spare respiratory capacity. (*n* = 3; ns, not significant; *p < 0.05; **p < 0.01; ****p < 0.0001). M) Intracellular ATP levels in A549 cells with altered *COA4* expression. (*n* = 3; ***p < 0.001). N) IF analysis of mitochondrial mass in A549 cells with *COA4* knockdown or overexpression, visualized using MitoTracker Red CMXRos. Scale bar: 50 µm. O) Transmission electron microscopy (TEM) images of mitochondrial ultrastructure in A549 cells following *COA4* knockdown and after rescue by *COA4* overexpression in the knockdown background. (*n* = 3). P) Mitochondrial ultrastructure analysis by structured illumination microscopy (SIM) in *COA4*‐modulated A549 cells. Scale bar: 5 µm. (*n* = 12 cells each; ***p < 0.001). Q) JC‐10 staining of mitochondrial membrane potential in A549 cells overexpressing *COA4* and treated with oligomycin. H⁺‐ATP synthase inhibitor Oligomycin (1 µM) for 24 h. Scale bar: 50 µm. (*n* = 3). The data are given as mean ± SD and compared by two‐tailed unpaired Student's t‐test (C‐E, H, M, N, and Q), one‐way ANOVA G,L). Significance levels are indicated as follows: ns, not significant; *p < 0.05; **p < 0.01; ***p < 0.001; ****p < 0.0001.

Previous studies have established COA4 as a critical assembly factor for mitochondrial COX in yeast, where it is essential for mitochondrial biogenesis.^[^ ^16^ ^]^ Although bioinformatic analyses have implicated *COA4* as a putative oncogene,^[^ ^18^ ^]^ its biological function in mammalian cells remains uncharacterized. To determine its subcellular localization, we performed immunofluorescence (IF) assays in 293T, A549, H23, and BEAS‐2B cells. Our results indicate that COA4 is predominantly localized in the mitochondria and cytoplasm, with a minor fraction present in the nucleus (**Figure** **1**A). Overexpression of GFP‐tagged COA4 in 293T and A549 cells confirmed these localization patterns and further revealed its accumulation in pseudopod‐like structures at the cell membrane periphery (Figure 1B). Given that yeast COA4 modulates COX enzymatic activity,^[^ ^16a^ ^]^ we hypothesized that a similar, evolutionarily conserved function exists in human cells. Enzymatic assays demonstrated that COA4 knockdown significantly reduced COX activity in 293T, A549, and H23 cells, whereas its overexpression enhanced enzymatic activity (Figure 1C).

Mammalian COX comprises 10 nuclear‐encoded subunits and three mitochondrial‐encoded subunits (MTCO1, MTCO2, MTCO3).^[^ ^20^ ^]^ We first assessed whether COA4 regulates mitochondrial‐encoded gene expression in mammalian cells. In A549 and H23 cells, *COA4* knockdown markedly decreased MTCO1 protein levels, while overexpression upregulated MTCO1 expression; in contrast, levels of MTCO2 and MTCO3 remained unchanged (Figure 1D and Figure S1A, Supporting Information). Importantly, re‐expression of *COA4* in knockdown cells restored MTCO1 protein levels (Figure 1E), even though *MTCO1* mRNA levels were unaltered (Figure S1B, Supporting Information), indicating that COA4 modulates MTCO1 stability post‐transcriptionally. This regulatory role was further validated in LUAD patient‐derived organoids, where *COA4* knockdown similarly reduced MTCO1 protein levels (Figure 1F and Figure S1C, Supporting Information). Protein stability assays confirmed that overexpression of *COA4* extended the half‐life of MTCO1 (Figure 1G). Given its partial nuclear localization, we next investigated COA4's impact on nuclear‐encoded COX subunits. In H23 and A549 cells, *COA4* knockdown significantly downregulated the transcripts of several nuclear‐encoded COX genes, including *COX5A*, *COX5B*, *COX6A*, *COX6B*, and *COX7A* (Figure 1H and Figure S1D, Supporting Information). However, levels of known mitochondrial transcriptional regulators (*TFAM*, *PGC1α*, *NRF2*) and mitochondrial DNA (mtDNA) copy number were not affected by *COA4* modulation (Figure S1E,F, Supporting Information).

To further explore the mechanisms underlying COA4‐mediated regulation of COX, proteomic analysis identified SLC25A3—a copper transporter integral to COX activity—as a potential interactor.^[^ ^21^ ^]^ AlphaFold 3 structural predictions suggested an interaction between COA4 and SLC25A3 (Figure 1I), which was confirmed by immunoprecipitation (IP) in A549 cells (Figure 1J) and Duolink proximity ligation assays (PLA) (Figure 1K). Notably, COA4 manipulation did not alter SLC25A3 protein levels (Figure S1G, Supporting Information). Further analysis revealed that *COA4* overexpression enhances mitochondrial copper transport. Notably, concomitant knockdown of *SLC25A3* abolished this effect (Figure S1H,I, Supporting Information). Consistently, with *SLC25A3* knockdown in *COA4*‐overexpressing cells, COX activity was significantly impaired (Figure S1J, Supporting Information). These data demonstrate that COA4 may functionally cooperate with SLC25A3 in copper‐dependent enzyme activation, consistent with yeast COA4's role in mitochondrial copper transport.^[^ ^16a^ ^]^

We next evaluated the impact of COA4 on mitochondrial metabolism. Seahorse analyses revealed that *COA4* overexpression elevated spare respiratory capacity, basal and maximal respiration, and ATP‐linked oxygen consumption rate (OCR), while *COA4* knockdown suppressed OXPHOS (Figure 1L). Correspondingly, intracellular ATP levels increased with *COA4* overexpression and decreased with its knockdown (Figure 1M and Figure S1K, Supporting Information). Furthermore, glycolytic capacity was inversely correlated with *COA4* expression (Figure S1L, Supporting Information).

Additional IF studies showed that altering COA4 expression significantly affected mitochondrial mass (Figure 1N and Figure S1M, Supporting Information). Electron microscopy demonstrated that *COA4* knockdown induced structural damage in mitochondria, characterized by swelling and reduced cristae density, which was rescued by *COA4* re‐expression (Figure 1O). Structured illumination microscopy (SIM) further confirmed that *COA4* depletion induced mitochondrial fragmentation, with partial restoration of mitochondrial morphology upon re‐expression (Figure 1P and Figure S1N, Supporting Information). Moreover, *COA4* overexpression markedly increased mitochondrial membrane potential, an effect that was partially reversed by treatment with the OXPHOS inhibitor oligomycin (Figure 1Q) or the COX inhibitor (NH_4_)_2_MoS_4_ (Figure S1O, Supporting Information). Notably, both oligomycin (Figure S1P, Supporting Information) and (NH_4_)_2_MoS_4_ (Figure S1Q, Supporting Information) attenuated the increase in mitochondrial mass induced by *COA4* overexpression.

In summary, these findings establish COA4 as a critical novel regulator of mitochondrial metabolic homeostasis in human cells, primarily through its role in modulating COX activity.

### 2.2 *COA4* is Upregulated in LUAD Tissues and Patient‐Derived Organoids and is Associated with Poor Prognosis

The aforementioned findings establish that COA4, originally identified as a regulator of mitochondrial COX in yeast, retains its functional role in modulating mitochondrial OXPHOS in human cells. Given the critical role of OXPHOS in tumor proliferation and metastasis, we next investigated the expression pattern and clinical relevance of COA4 in cancers, particularly in LUAD. Pan‐cancer analysis using the TIMER2.0 database revealed significantly elevated *COA4* expression in multiple malignancies—including LUAD, pancreatic adenocarcinoma (PAAD), colon adenocarcinoma (COAD), breast cancer (BRCA), hepatocellular carcinoma (HCC), and clear cell renal cell carcinoma (ccRCC)—compared to normal tissues (Figure S2A, Supporting Information), suggesting an oncogenic role for COA4.

**Figure 2:**
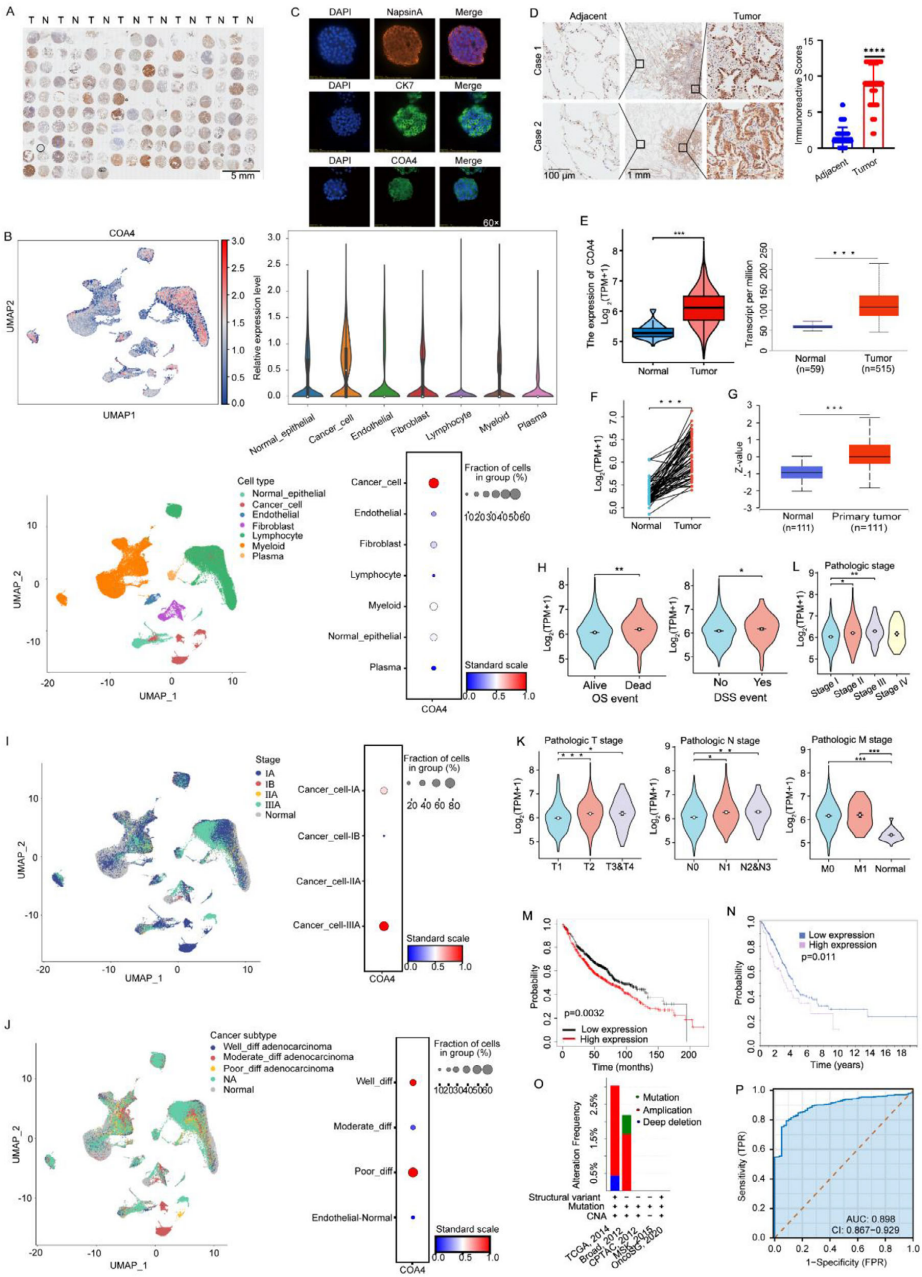
*COA4* is upregulated in LUAD tissues and patient‐derived organoids and is associated with poor prognosis. A) IHC analysis of COA4 expression in a LUAD tissue microarray comprising 75 paired tumor and adjacent normal tissues. B) Single‐cell RNA sequencing (GSE210347) showing the expression profile of *COA4* across various cell types in LUAD. C) IF analysis of CK7, Napsin A, and COA4 in LUAD patient‐derived organoids (PDOs). (*n* = 3). D) Representative IHC images illustrating COA4 expression in LUAD tissues compared to adjacent normal tissues. (*n* = 12 per group; ****p < 0.0001). E) *COA4* mRNA expression analysis in LUAD using GEPIA2 (left) and UALCAN (right) databases, comparing unpaired tumor and normal samples. ***p < 0.001. F) *COA4* mRNA expression analysis in LUAD using the Xiantao Academic database, comparing paired tumor and normal samples. ***p < 0.001. G) COA4 protein expression in LUAD and normal tissues as assessed by UALCAN. ***p < 0.001. H) Correlation of *COA4* expression with overall survival (OS) and disease‐specific survival (DSS) based on TCGA datasets. *p < 0.05; **p < 0.01. I,J) Correlation of *COA4* expression with pathological stage I) and tumor differentiation J) using single‐cell RNA sequencing data. K,L) Correlation of *COA4* expression with tumor (T), node (N), and metastasis (M) classification (K) and with pathological stage (L) using TCGA datasets. M,N) Overall survival analysis of LUAD patients based on *COA4* mRNA (M) and protein (N) expression levels. O) Analysis of *COA4* gene mutations in LUAD using cBioPortal. P) Diagnostic performance of *COA4* in distinguishing LUAD from normal tissue. The data are presented as mean ± SD and compared by Student's *t‐*test (D, E, F, G, H, and L) and one‐way ANOVA (K). The Log‐rank (Mantel–Cox) test was used for survival analyses (M, N). Significance levels are indicated as follows: ns, not significant; *p < 0.05; **p < 0.01; ***p < 0.001; ****p < 0.0001.

To further explore COA4 expression in LUAD, we first examined clinical specimens. Immunohistochemical analysis of a tissue microarray containing 75 paired LUAD and adjacent normal tissues revealed pronounced upregulation of COA4 in tumor samples (**Figure** **2**A). Single‐cell RNA sequencing data (GSE210347) corroborated these findings, demonstrating that NSCLC cells express significantly higher levels of *COA4* than normal pulmonary epithelial cells, endothelial cells, fibroblasts, and lymphocytes (Figure 2B).

To validate the elevated expression of COA4 in LUAD, we established patient‐derived LUAD organoids. IF confirmed robust expression of LUAD markers Napsin A and CK7, verifying successful organoid generation. Notably, the expression level of COA4 was close to that of Napsin A and CK7, and was also highly expressed in organoids (Figure 2C). Subsequent validation in an independent cohort of clinical LUAD specimens revealed significantly higher *COA4* levels in tumors versus matched adjacent tissues (Figure 2D). This finding was further supported by Western blot analysis (Figure S2B, Supporting Information) and by the observation of elevated *COA4* expression in LUAD cell lines relative to normal controls (Figure S2C, Supporting Information and **Table** **1**). Bioinformatic analyses using the GEPIA2, UALCAN, and Xiantao platforms further confirmed the overexpression of *COA4* in LUAD tissues compared to normal lung tissues (Figure 2E,F). Protein‐level analysis via UALCAN also demonstrated elevated *COA4* in LUAD samples (Figure 2G). Analysis of tumor *COA4* expression levels revealed that deceased LUAD patients had significantly higher levels than surviving patients (Figure 2H). Furthermore, among deceased patients, those who died from cancer‐related causes exhibited higher *COA4* levels than those who died from other causes (Figure 2H).

**Table 1:** Characteristics of cell lines used in this study.

Cell line	Cancer type	Tissue o rigin	KRAS Mutation	EGFR Mutation	TP53 Status
A549	NSCLC (LUAD)	Lung adenocarcinoma	G12S	Wild‐type	Wild‐type
H23	NSCLC (LUAD)	Lung adenocarcinoma	G12C	Wild‐type	Mutant
H460	NSCLC (large cell)	Lung	Q61H	Wild‐type	Mutant
H1299	NSCLC	Lung carcinoma; Metastatic site: Lymph node	Wild‐type	Wild‐type	Null
H1975	NSCLC (LUAD)	Lung adenocarcinoma	Wild‐type	L858R/T790M	Mutant
HCC827	NSCLC (LUAD)	Lung adenocarcinoma	Wild‐type	E746‐A750 deletion	Mutant
ASPC‐1	PDAC	Pancreatic adenocarcinoma	G12D	Wild‐type	Mutant

Additionally, single‐cell sequencing data associated elevated *COA4* expression with advanced pathological stages and poor tumor differentiation (Figure 2I,J). Analysis of TCGA data established significant correlations between *COA4* upregulation and TNM staging—particularly T and N stages—as well as with higher pathological grades (Figure 2K,L). Importantly, both elevated *COA4* mRNA (Figure 2M and Figure S2D, Supporting Information) and protein levels (Figure 2N) predicted poor prognosis in LUAD. Although *COA4* was also overexpressed in lung squamous cell carcinoma (Figure S2A and S2E, Supporting Information), no significant survival correlation was observed in this subtype (Figure S2F, Supporting Information), thereby focusing our investigation on LUAD. Notably, COA4 mutations were detected in approximately 3% of LUAD cases (Figure 2O). Diagnostic analysis revealed an area under the curve (AUC) of 0.898 for *COA4* in distinguishing LUAD from normal tissue (Figure 2P), with robust performance across T (AUC = 0.898), N (0.902), and M (0.903) stages, as well as across pathological grades (AUC = 0.897; Figure S2G, Supporting Information). Finally, to evaluate whether *COA4* expression serves as an independent prognostic factor, we performed multivariate Cox regression analysis on the TCGA LUAD cohort (*n* = 779). High *COA4* expression was associated with a 53.3% increase in overall mortality risk compared to low expression (**Table** **2**), indicating that elevated *COA4* is an independent adverse prognostic marker for overall survival in LUAD patients.

**Table 2:** Cox proportional hazards regression analysis of COA4 expression in LUAD.

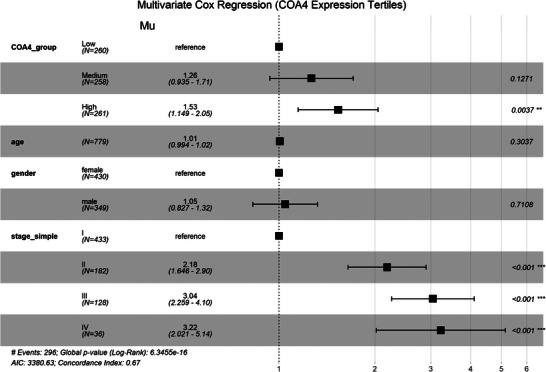

Note: The data were analyzed by multivariable Cox proportional hazards regression. Significance levels are indicated as follows: ns, not significant; *p < 0.05, **p < 0.01, ***p < 0.001.

Collectively, these results establish *COA4* as a novel oncogenic candidate and prognostic biomarker in LUAD, characterized by tumor‐specific overexpression, stage‐dependent elevation, and a strong association with adverse clinical outcomes.

### 2.3 *COA4* Promotes Metastasis of LUAD Cells via Regulating OXPHOS In Vitro and In Vivo

Our findings indicated that COA4, a novel regulator of mitochondrial OXPHOS, was highly expressed in LUAD cells and correlated with poor prognosis. However, its mechanistic role in tumorigenesis and progression had not been previously described. To evaluate its functional significance in LUAD pathogenesis, we established stable *COA4* knockdown and overexpression cell lines in A549 and H23 cells (Figure S3A, Supporting Information), and generated CRISPR‐Cas9‐mediated *COA4* knockout lines (Figure S3B, Supporting Information). Initial assays revealed that *COA4* manipulation did not significantly impact colony formation (Figure S3C, Supporting Information), cell proliferation as assessed by CCK‐8 assay (Figure S3D, Supporting Information), EdU incorporation (Figure S3E, Supporting Information), or anchorage‐independent growth in soft agar (Figure S3F, Supporting Information). Likewise, subcutaneous xenograft models demonstrated no significant differences in tumor volume or weight (Figure S3G, Supporting Information), even under glucose‐deprived conditions simulating nutrient stress (Figure S3H, Supporting Information). Interestingly, however, in patient‐derived LUAD organoids, *COA4* knockdown markedly impaired organoid formation and expansion, whereas its overexpression enhanced these processes (Figure S3I, Supporting Information), suggesting that COA4 exerts context‐dependent regulatory effects.

**Figure 3:**
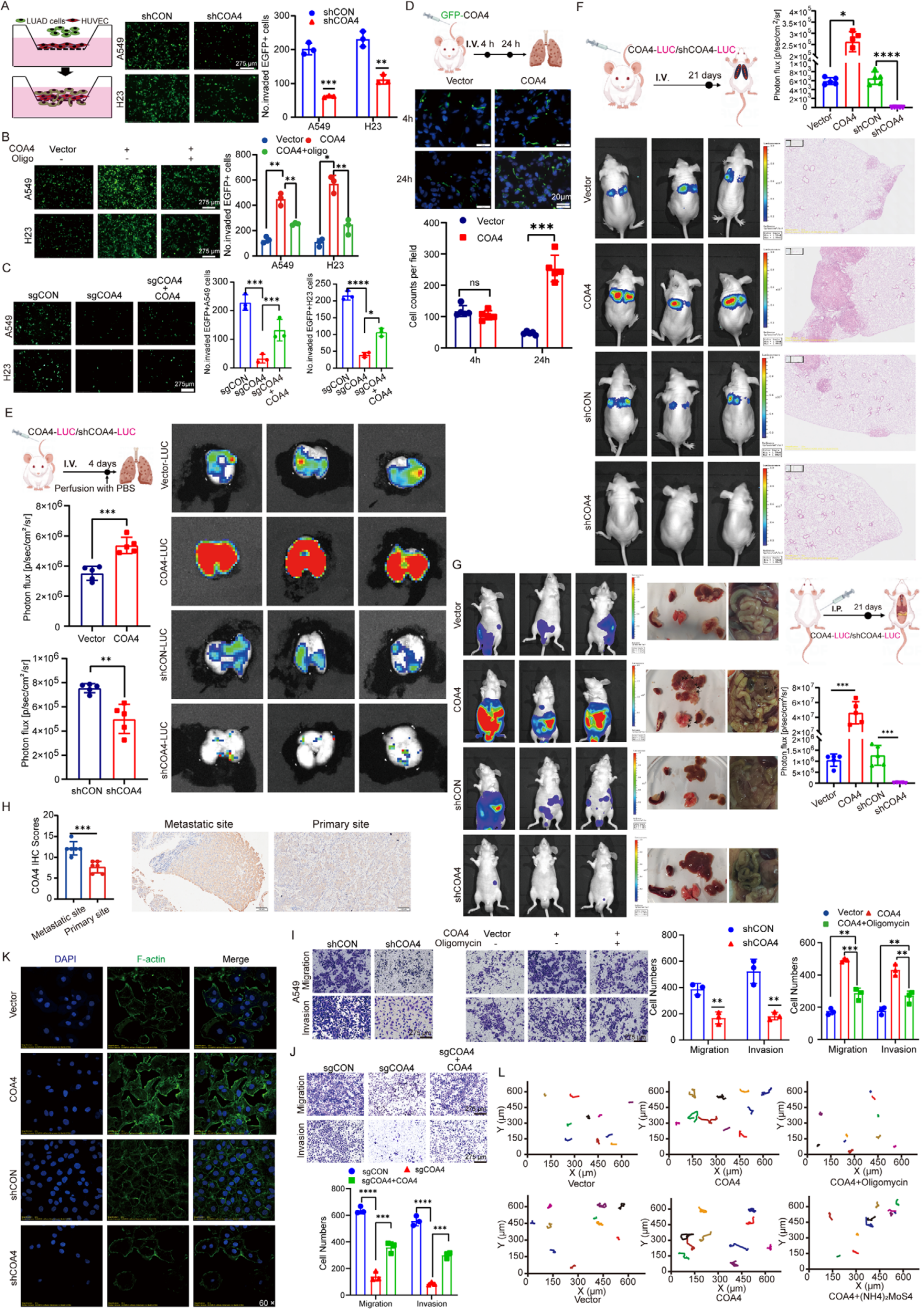
COA4 promotes metastasis of LUAD cells via driving metabolic reprogramming towards OXPHOS in vitro and in vivo. A) Trans‐endothelial migration assays were performed to assess the ability of GFP‐labeled A549 and H23 LUAD cells to traverse HUVEC monolayers following *COA4* knockdown. Scale bar: 275 µm (*n* = 3; **p < 0.01; ***p < 0.001). B) The trans‐endothelial migration capacity of *COA4*‐overexpressing A549 and H23 cells treated with the OXPHOS inhibitor oligomycin was evaluated after 24 h. Scale bar: 275 µm (*n* = 3; *p < 0.05; **p < 0.01) C) Trans‐endothelial migration assays were used to compare the ability of A549 or H23 cells to traverse HUVEC monolayers after *COA4* knockout and subsequent rescue by *COA4* re‐expression. Scale bar: 275 µm (*n* = 3; *p < 0.05; ***p < 0.001; ****p < 0.0001). D) In vitro bioluminescence imaging (BLI) was conducted following tail vein injection of either GFP control or GFP‐*COA4* A549 cells, with fluorescence imaging of lung tissue sections performed at 4 and 24 h. Scale bar: 20 µm (*n* = 5 per group, ns, not significant; ***p < 0.001). E) In vivo extravasation in mouse lungs was assessed after tail vein injection of luciferase‐tagged A549 cells with *COA4* knockdown or overexpression (*n* = 5 per group, **p < 0.01; ***p < 0.001). F) In vivo metastasis was evaluated following tail vein injection of luciferase‐tagged A549 cells with altered *COA4* expression; fluorescence intensity, H&E staining, and statistical analysis were used to quantify metastatic burden (*n* = 5 per group, *p < 0.05; ****p < 0.0001). G) Intraperitoneal metastasis was analyzed using luciferase‐tagged A549 cells with *COA4* knockdown or overexpression, with fluorescence intensity measurements and assessment of adhesion status, supported by statistical analysis (*n* = 5 per group, ***p < 0.001). H) Immunohistochemical analysis of *COA4* expression in primary versus metastatic LUAD sites (*n* = 6 per group, ***p < 0.001). I,J) Transwell assays quantified cell migration and invasion in A549 cells: (I, left) in *COA4*‐knockdown cells, (I, right) in *COA4*‐overexpressing cells treated with oligomycin, and (J) in *COA4*‐knockout cells with subsequent *COA4* re‐expression. Scale bar: 275 µm (*n* = 3; **p < 0.01; ***p < 0.001; ****p < 0.0001) K) IF analysis of F‐actin in A549 cells with *COA4* knockdown or overexpression, with nuclei counterstained using DAPI. Scale bar: 50 µm (*n* = 3) L) Single‐cell time‐lapse imaging assays were performed on *COA4*‐overexpressing A549 cells treated with oligomycin (top) or (NH_4_)_2_MoS_4_ (100 µM, Complex IV inhibitor) (bottom), with corresponding movement trajectory plots. (*n* = 3). The data are given as mean ± SD and compared by Student's t test (A–K). Significance levels are indicated as follows: ns, not significant; *p < 0.05; **p < 0.01; ***p < 0.001; ****p < 0.0001.

Given that trans‐endothelial migration is a key step in metastasis,^[^ ^22^ ^]^ we assessed COA4's role in this process using trans‐endothelial migration assays. In both A549 and H23 cells, overexpression of *COA4* significantly enhanced the ability of the cells to traverse an endothelial cell layer, whereas *COA4* knockdown reduced this capacity (**Figure** **3**A,B). Furthermore, in *COA4*‐knockout cells, trans‐endothelial migration ability was markedly suppressed; notably, subsequent re‐expression of *COA4* restored this ability (Figure 3C). Similar results were obtained in H1299 cells upon *COA4* knockdown or overexpression, as well as in *COA4*‐knockout cells with *COA4* re‐expression (Figure S4A,B, Supporting Information).

To investigate the role of *COA4* in tumor cell extravasation in vivo, we performed lung dissections at 4 and 24 h post‐tail vein injection of GFP⁺ A549 cells overexpressing *COA4* in nude mice. At 24 h, a significantly higher number of *C* *OA4*‐overexpressing GFP⁺ cells were detected in the lung tissue compared to vector controls (Figure 3D). In experimental metastasis models, intravascular tumor cells were removed by deep perfusion; therefore, the luminescence signal detected after perfusion reflected extravasated and colonized cells. When stable *COA4*‐overexpressing luciferase cells and *COA4*‐knockdown luciferase cells were intravenously injected and analyzed 4 days post‐injection, mice injected with *COA4*‐overexpressing luciferase cells exhibited significantly higher luminescence relative to vector control cells, whereas mice injected with *COA4*‐knockdown luciferase cells showed reduced luminescence (Figure 3E). Moreover, overexpression of *COA4*‐GFP in A549 cells induced the formation of pseudopodia‐like structures at the cell membrane that colocalized with F‐actin, implicating COA4 in pseudopodia formation (Figure 6J). These data support a role for COA4 in promoting both extravasation and subsequent colonization during metastasis.

Further in vivo studies demonstrated that tail vein injection of stable *COA4*‐overexpressing A549 cells led to a significant enhancement in lung metastasis, while *COA4* knockdown resulted in markedly fewer metastatic foci (Figure 3F). H&E staining confirmed these findings (Figure 3F). In intraperitoneal injection models, *COA4* overexpression promoted LUAD cell adhesion and invasion into multiple organs—including the peritoneum, colon, small intestine, liver, and lung—along with enhanced inter‐organ adhesions, whereas COA4 knockdown markedly reduced these parameters (Figure 3G). Additional analyses showed that high *COA4* expression correlated with lymph node metastasis (Figure S4C, Supporting Information and Figure 2K). Consistently, immunohistochemical evaluation of clinical LUAD specimens revealed higher COA4 expression in metastatic lesions compared to primary tumors (Figure 3H).

Transwell assays further demonstrated that *COA4* overexpression significantly enhanced invasion and migration in A549, H23, and H1299 cells, whereas its knockdown suppressed these capabilities (Figure 3I and Figure S4D,E, Supporting Information). Similarly, CRISPR‐Cas9‐mediated knockout of *COA4* significantly inhibited cell invasion and migration, and re‐expression of *COA4* rescued these functions (Figure 3J). Time‐lapse imaging of single‐cell motility confirmed that *COA4* knockdown significantly reduced cell movement, while its overexpression promoted motility (Figure S4F, Supporting Information). Consistent with these observations, scratch assays revealed decreased migration in *COA4*‐depleted A549 and H23 cells, an effect reversed by *COA4* re‐expression (Figure S4G,H, Supporting Information).

Transcriptomic sequencing of A549 cells following *COA4* knockdown revealed significant enrichment of pathways related to cell motility—including regulation of the actin cytoskeleton, focal adhesion, and extracellular matrix (ECM)‐receptor interaction (Figure S5A,E, Supporting Information). Focal adhesion kinase (FAK), a critical tyrosine kinase downstream of the focal adhesion and extracellular matrix signaling pathways, is closely related to tumor cell motility.^[^ ^23^ ^]^ Notably, *COA4* manipulation did not alter total FAK protein levels; however, *COA4* knockdown significantly reduced FAK phosphorylation, while overexpression markedly increased it (Figure S4I, Supporting Information). Furthermore, *FAK* knockdown in A549 and H1299 cells inhibited invasion and migration, partially attenuating the effects of *COA4* overexpression (Figures S4J and S6A, Supporting Information). IF analysis revealed that *COA4* overexpression dramatically altered F‐actin morphology, leading to the formation of elongated, thick, and abundant pseudopodia‐like protrusions, which were reversed upon *COA4* knockdown (Figure 3K and Figure S6B, Supporting Information). Collectively, these in vitro and in vivo data established that *COA4* is a critical driver of trans‐endothelial migration, extravasation, and tissue colonization during LUAD metastasis.

Given our previous findings implicating COA4 in mitochondrial OXPHOS and emerging evidence indicates that metastatic tumors exhibit high OXPHOS capacity,^[^ ^13b^ ^]^ we investigated the role of mitochondrial metabolism in COA4‐mediated invasion and migration. Previous studies have identified cysteines within the CHCH domain as critical residues for mitochondrial targeting.^[^ ^24^ ^]^ Sequence analysis revealed that *COA4* harbors a CHCH domain. Therefore, we generated a *COA4* mutant by substituting cysteine residues within its CHCH domain with serines (mut*COA4*‐CS). This mutation significantly impaired the mitochondrial localization of COA4 (Figure S6C, Supporting Information). Consistently, overexpression of wild‐type *COA4* in A549 cells robustly promoted cell invasion and migration. In contrast, while mut*COA4*‐CS overexpression also exerted a partial pro‐invasive and pro‐migratory effect, this effect was significantly reduced compared to WT *COA4* (Figure S6D, Supporting Information). Collectively, these findings suggest that defective mitochondrial import of COA4 attenuates its ability to drive mitochondrial oxidative phosphorylation, thereby limiting its pro‐metastatic capacity in tumor cells. In A549 and H23 cells, treatment with the OXPHOS inhibitor oligomycin partially abrogated the enhanced migration and invasion induced by *COA4* overexpression (Figure 3I), as did the COX inhibitor (NH_4_)_2_MoS_4_, which also significantly suppressed cell motility in time‐lapse imaging assays (Figure 3L). Moreover, oligomycin significantly inhibited the trans‐endothelial migration of *COA4*‐overexpressing cells (Figure 3B), and (NH_4_)_2_MoS_4_ similarly suppressed COA4‐driven invasion and migration (Figure S11D, Supporting Information). Oligomycin and (NH_4_)_2_MoS_4_ treatments both attenuated the COA4‐induced increase in FAK phosphorylation (Figures S4K and S6E, Supporting Information) and disrupted F‐actin expression and distribution (Figure S6F,G, Supporting Information). In vivo, administration of the OXPHOS inhibitor significantly reduced lung metastases of *COA4*‐overexpressing A549 cells (Figure 6Q), and treatment with (NH_4_)_2_MoS_4_ similarly suppressed COA4‐induced lung metastasis (Figure S11F, Supporting Information). To further investigate the role of COA4 in LUAD metastasis, we crossed *COA4^fl/fl^* mice with *TP53^fl/fl^;KRAS^G12D^* mice to generate *TP53^fl/fl^;KRAS^G12D^;COA4^fl/fl^* triple‐mutant mice. Adenoviral delivery of Cre recombinase (Ad‐Cre) via intratracheal instillation with AAV9‐CMV‐Cre achieved *KRAS* ^G12D^ mutant activation and simultaneous deletion of *TP53* and *COA4*. Notably, COA4 knockout significantly suppressed lymph node metastasis in LUAD (Figure S6H,I, Supporting Information).

To further elucidate the relationship between COX activity, mitochondrial OXPHOS capacity, and the pathological grade and TNM stage of LUAD, we analyzed the LUAD dataset (GSE30219) for the expression of OXPHOS and COX genes, summarizing these as a combined OXPHOS and COX score. Stage IIB and stage IIIA tumors exhibited higher OXPHOS and COX scores than stage I tumors (Figure S7A,B, Supporting Information). Moreover, these scores correlated with tumor T and N stages, with higher scores associated with more advanced T and N stages (Figure S7C,D, Supporting Information).

Collectively, our results indicated that COA4 promotes LUAD cell migration and invasion primarily by regulating COX‐driven OXPHOS.

### 2.4 Mutant KRAS Promotes *COA4* Expression via PI3K Signaling Axis

We further investigated factors driving *COA4* upregulation in LUAD cells. Given that hypoxia and nutrient deprivation characterize the tumor microenvironment, we first assessed *COA4* expression under low oxygen and low glucose conditions. Neither *COA4* mRNA nor protein levels were significantly altered by these stressors (Figure S8A, Supporting Information).

**Figure 4:**
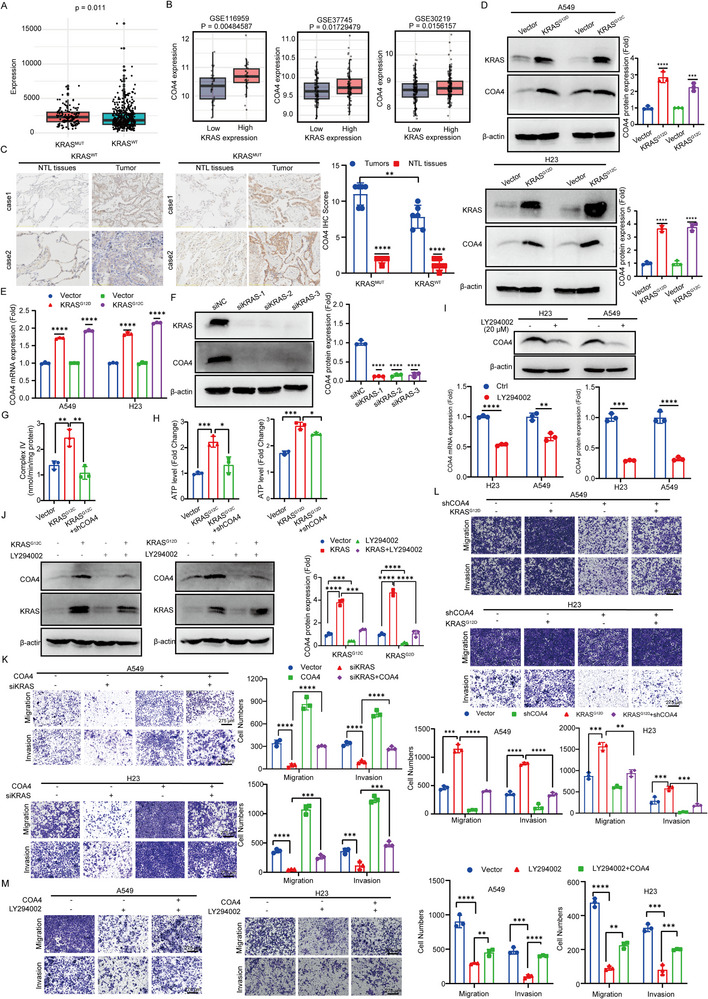
Mutant *KRAS* promotes *COA4* expression via PI3K signaling axis. A) Analysis of *COA4* expression in LUAD tissues with KRAS wild‐type and mutant status using TCGA datasets. B) Analysis of *COA4* expression in LUAD with high versus low *KRAS* expression using the GSE116959, GSE37745, and GSE30219 datasets. C) IHC analysis of COA4 expression in LUAD tissues with KRAS wild‐type (wt) and mutant (mut) status (*n* = 6 per group, **p < 0.01; ****p < 0.0001). D,E) Evaluation of COA4 protein (D) and mRNA (E) levels in A549 and H23 cells following overexpression of *KRAS* ^G12C^ and *KRAS* ^G12D^. (*n* = 3, ***p < 0.001; ****p < 0.0001) F) Western blot analysis of COA4 protein expression in A549 cells after *KRAS* knockdown.(*n* = 3, ****p < 0.0001). G) Assessment of Complex IV enzyme activity in A549 cells overexpressing *KRAS* ^G12C^, and following subsequent *COA4* knockdown. (*n* = 3, **p < 0.01) H) Analysis of ATP levels in A549 cells with *KRAS* ^G12C^ overexpression and subsequent *COA4* knockdown.(*n* = 3, *p < 0.05; ****p < 0.0001) I) Western blot and RT‐qPCR analysis of *COA4* expression in A549 and H23 cells treated with the PI3K inhibitor LY294002 (20 µM) for 48 h. (*n* = 3; **p < 0.01; ***p < 0.001; ****p < 0.0001) J) Western blot analysis of COA4 expression in *KRAS* ^G12C^‐ or *KRAS* ^G12D^‐overexpressing A549 cells following LY294002 treatment. (*n* = 3; ***p < 0.001; ****p < 0.0001) K) Transwell assays assessing the impact of *COA4* overexpression on the migration and invasion of A549 and H23 cells following *KRAS* knockdown. Scale bar: 275 µm (*n* = 3; ***p < 0.001; ****p < 0.0001) L) Transwell assays evaluating the effects of *COA4* knockdown on the migration and invasion of A549 and H23 cells overexpressing *KRAS* ^G12D^. Scale bar: 275 µm (*n* = 3; **p < 0.01; ***p < 0.001; ****p < 0.0001) M) Transwell assays analyzing the impact of *COA4* overexpression on the migration and invasion of A549 and H23 cells treated with LY294002. Scale bar: 275 µm (*n* = 3; **p < 0.01; ***p < 0.001; ****p < 0.0001) The data are given as mean ± SD and compared by two‐tailed unpaired Student's *t*‐test (A‐M). Significance levels are indicated as follows: ns, not significant; *p < 0.05; **p < 0.01; ***p < 0.001; ****p < 0.0001.

*KRAS* mutations represent prevalent genetic alterations in LUAD, yet effective targeted therapies remain limited. Targeting downstream KRAS effectors thus offers a promising therapeutic strategy. Although prior studies suggested *KRAS* regulates mitochondrial COX, the underlying mechanisms were unclear. Analysis of TCGA data revealed significantly higher *COA4* expression in *KRAS*‐mutant LUAD tissues versus wild‐type *KRAS* controls (**Figure** **4**A). Furthermore, LUAD tissues with high KRAS expression exhibited elevated *COA4* levels compared to low‐*KRAS*‐expressing tissues (Figure 4B). Immunohistochemistry confirmed increased COA4 protein in KRAS‐mutant lung tissues relative to wild‐type samples (Figure 4C).

To directly assess the KRAS‐COA4 relationship, we overexpressed *KRAS* ^G12D^ and *KRAS* ^G12C^ in A549 and H23 cells. Protein (Figure 4D) and mRNA (Figure 4E) analyses demonstrated that mutant KRAS significantly upregulates *COA4* expression. Conversely, *KRAS* knockdown markedly downregulated *COA4* in LUAD cells (Figure 4F). In *KRAS* ^G12C^‐overexpressing A549 cells, COX activity increased significantly; this effect was attenuated by *COA4* knockdown (Figure 4G). Similarly, ATP levels increased upon *KRAS* ^G12C^ or *KRAS* ^G12D^ overexpression and were partially reversed by *COA4* knockdown (Figure 4H). These findings demonstrate that *COA4* is a downstream effector of *KRAS*.

In normal lung epithelial BEAS‐2B cells, *KRAS* ^G12D^ and *KRAS* ^G12C^ overexpression also upregulated *COA4* (Figure S8B, Supporting Information), indicating *KRAS*‐driven *COA4* induction occurs in non‐cancerous pulmonary cells. Analysis across cell lines revealed elevated *COA4* expression in *KRAS*‐mutant lines (A549, H23, H460) and some wild‐type lines (e.g., H1299) (Figure S2C, Supporting Information). Moreover, data from the DepMap portal^[^ ^25^ ^]^ further demonstrated that *COA4* is overexpressed in multiple *KRAS*‐mutant cell lines (e.g., NCI‐H650, HCC2108), in *HRAS*‐mutant lines (e.g., NCI‐H1915, NCI‐H1693), and even in several *KRAS* wild‐type lines (e.g., NCI‐H838, HCC1833) (Figure S8C, Supporting Information). These observations suggest that while oncogenic *KRAS* is a key regulator of *COA4* in LUAD cells, additional factors may also contribute to its regulation. Notably, modulation of COA4 expression did not affect KRAS levels (Figure S8D, Supporting Information).

*KRAS* mutations are prevalent in other malignancies, including PAAD and COAD, occurring in 90% and 45% of cases, respectively.^[^ ^26^ ^]^ We found *COA4* significantly overexpressed in these *KRAS*‐mutant tumors (Figure S8E,F, Supporting Information). Elevated COA4 protein levels correlated with poor prognosis in PAAD (p = 0.0012), though this association was not statistically significant in COAD (p = 0.08) (Figure S8G, Supporting Information). These results implicate *COA4* in the progression of diverse *KRAS*‐driven malignancies. As *COA4*'s pro‐metastatic role in PAAD remains unreported, we selected the *KRAS* ^G12D^‐mutant ASPC‐1 cell line from DepMap/MetMap databases—characterized by high *COA4* expression and metastatic competence—and established stable *COA4*‐knockdown (sh*COA4*‐luciferase) cells (Figure S8H, Supporting Information). *COA4* depletion significantly reduced ASPC‐1 cell invasion and migration (Figure S8I, Supporting Information). Tail vein injection assays demonstrated that *COA4* knockdown markedly suppressed lung colonization and metastasis at 4 days post‐injection versus controls (Figure S8J, Supporting Information). These findings suggest *COA4*’s pro‐metastatic function extends beyond LUAD to other *KRAS*‐mutant cancers.

**Figure 5:**
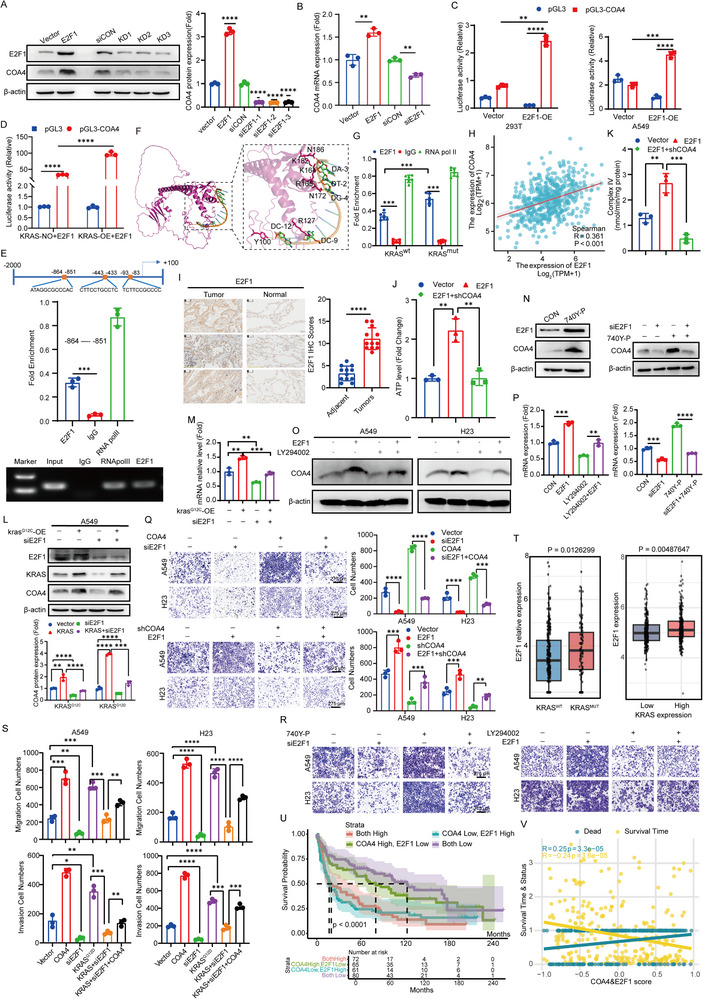
E2F1 directly binds to *COA4* promoter and promotes its expression under KRAS mutation. A,B) Western blot (A) and RT‐qPCR (B) analyses of *COA4* expression in A549 cells following *E2F1* overexpression or knockdown. (*n* = 3; **p < 0.01; ****p < 0.0001) C) Luciferase reporter assay confirming that E2F1 directly regulates *COA4* transcription in 293T and A549 cells. (*n* = 3; **p < 0.01; ***p < 0.001; ****p < 0.0001) D) Luciferase reporter assay demonstrating that KRAS enhances E2F1‐mediated transcriptional activation of the *COA4* promoter in 293T cells. (*n* = 3; ****p < 0.0001) E) ChIP‐qPCR analysis identifying the specific E2F1 binding region within the *COA4* promoter in A549 cells. (*n* = 3; ***p < 0.001) F) AlphaFold 3 molecular docking of the *COA4* promoter with E2F1, supporting their direct interaction. G) Chromatin immunoprecipitation followed by qPCR (ChIP‐qPCR) was performed to evaluate E2F1 binding to the *COA4* promoter in normal tissues and KRAS‐mutant tumor tissues. Data are presented as mean ± SD (*n* = 6, ***p < 0.001) H) Spearman's rank correlation analysis of *COA4* and *E2F1* expression in LUAD using the GEPIA2 database. I. IHC analysis of *E2F1* expression in LUAD tumor versus normal tissues (*n* = 12 per group; ****p < 0.0001) J) ATP quantification in *E2F1*‐overexpressing A549 cells following *COA4* knockdown.(*n* = 3; **p < 0.01) K) Complex IV enzyme activity in *E2F1*‐overexpressing A549 cells after *COA4* knockdown. (*n* = 3; **p < 0.01) L,M) Western blot (L) and RT‐qPCR (M) analyses of *COA4* expression in *KRAS* ^G12C^‐overexpressing A549 cells following *E2F1* knockdown. (*n* = 3; **p < 0.01; ***p < 0.001; ****p < 0.0001) N) Western blot analysis of COA4 expression in A549 cells treated with the PI3K agonist 740Y‐P(15 µM) for 48 h (left), and in 740Y‐P‐treated A549 cells following *E2F1* knockdown (right).(*n* = 3) O) Western blot analysis of COA4 expression in A549 and H23 cells treated with the PI3K inhibitor LY294002 following *E2F1* overexpression. (*n* = 3) P) RT‐qPCR analysis of *COA4* mRNA levels in A549 cells treated with LY294002 following E2F1 overexpression (left), and in A549 cells treated with 740Y‐P following E2F1 knockdown (right). (*n* = 3; **p < 0.01; ***p < 0.001; ****p < 0.0001) Q) Transwell assays assessing migration of *COA4*‐overexpressing A549 and H23 cells following *E2F1* knockdown (top), and of COA4‐knockdown cells following *E2F1* overexpression (bottom). Scale bar: 275 µm (n = 3; **p < 0.01; ****p < 0.0001) R) Transwell assays analyzing migration of A549 and H23 cells treated with 740Y‐P following *E2F1* knockdown (left), and with LY294002 following *E2F1* overexpression (right). Scale bar: 275 µm (*n* = 3) S) Transwell assays evaluating migration and invasion in A549 and H23 cells following *KRAS* ^G12D^ overexpression, *E2F1* knockdown, and *COA4* overexpression. (*n* = 3; *p < 0.05; **p < 0.01; ***p < 0.001; ****p < 0.0001) T) Analysis of *E2F1* expression in LUAD tissues: left, comparing *KRAS* wild‐type versus *KRAS* mutant samples using TCGA data; right, comparing LUAD tissues with high versus low *KRAS* expression using combined datasets (GSE30219, GSE37745, GSE118370, and GSE140797). U) Survival analysis based on *COA4* and *E2F1* expression levels using the GSE30219 database. V) Combined analysis of COA4 and E2F1 expression with survival status and overall survival time, performed using the linkET package on the GSE30219 dataset. The data are given as mean ± SD and compared by Student's *t* test (A–E, G, I–T), Spearman's rank correlation analysis (H). The Log‐rank (Mantel–Cox) test was used for survival analyses (U,V). Significance levels are indicated as follows: ns, not significant; *p < 0.05; **p < 0.01; ***p < 0.001; ****p < 0.0001.

To elucidate the downstream signaling pathway mediating *KRAS* regulation of *COA4*, we evaluated inhibitors targeting four classical KRAS effector pathways: MAPK, PI3K, RAL, and JAK/STAT. Only PI3K inhibition significantly suppressed *COA4* expression at mRNA and protein levels, while other inhibitors showed minimal effects (Figure 4I and S8K, Supporting Information). Furthermore, PI3K activation with agonist 740Y‐P increased *COA4* expression (Figure 5N), and PI3K inhibitor treatment partially abrogated *KRAS*‐induced *COA4* upregulation (Figure 4J, Supporting Information).

Functional assays revealed that *KRAS* knockdown in A549 and H23 cells reduced invasion and migration—effects partially rescued by *COA4* overexpression (Figure 4K). Similarly, *KRAS* ^G12D^ overexpression enhanced invasion and migration, whereas subsequent *COA4* knockdown attenuated these phenotypes (Figure 4L). PI3K inhibitor treatment likewise suppressed LUAD cell invasion and migration, an effect partially reversed by *COA4* overexpression (Figure 4M). Collectively, these results indicate that in LUAD cells, mutant *KRAS* drives *COA4* expression via the PI3K signaling axis, thereby promoting cellular invasion and migration.

### 2.5 E2F1 Directly Binds to *COA4* Promoter and Drives Its Expression under KRAS Mutation

No upstream transcription factors regulating *COA4* have been reported. Using JASPAR and ChIP‐Atlas databases, we predicted potential *COA4* regulators, including *TCF4*, *ZNF460*, *SP1*, *STAT4*, *ETS1*, *KLF15*, *YY1*, and *E2F1*. Among these, only *E2F1* overexpression consistently enhanced *COA4* transcription in A549 and H23 cells (**Figure** **5**A and Figure S9A, Supporting Information). *E2F1* overexpression significantly increased *COA4* mRNA and protein levels, while *E2F1* knockdown reduced its expression (Figure 5A,B and Figure S9B,C, Supporting Information). Dual‐luciferase assays in 293T and A549 cells confirmed E2F1's direct regulation of *COA4* transcription (Figure 5C), and *KRAS* ^G12D^co‐expression amplified E2F1‐mediated *COA4* promoter activation (Figure 5D). In silico analysis predicted three E2F1 binding sites in the *COA4* promoter (–864 to –851; –443 to –433; –93 to –83). ChIP‐qPCR demonstrated specific E2F1 binding to the –864 to –851 region (Figure 5E, and Figure S9D, Supporting Information). AlphaFold 3 modeling confirmed E2F1 interaction with the “ATAGGCGCCCAC” sequence (Figure 5F, Supporting Information). In *KRAS*‐mutant LUAD tissues, E2F1 specifically bound this promoter sequence and regulated *COA4* transcription (Figure 5G). These results establish E2F1 as a direct transcriptional regulator of *COA4* in LUAD.

E2F1 transcription factors regulate genes involved in cell cycle control and tumorigenesis, but their role in LUAD remains incompletely characterized. GEPIA2 and Xiantao Academic analyses revealed positive correlations between *E2F1* and *COA4* mRNA levels in LUAD (Figure 5H and Figure S9E, Supporting Information). Moreover, multiple tumor types—such as PAAD and COAD, where *COA4* is highly expressed—also exhibit a positive correlation between *E2F1* and *COA4* (Figure S9F,G, Supporting Information). IHC further confirmed that *E2F1* is overexpressed in LUAD tissues compared to normal lung tissues (Figure 5I), and both GEPIA2 and Xiantao Academic analyses demonstrated elevated *E2F1* mRNA levels in LUAD (Figure S9H, Supporting Information). Critically, high *E2F1* expression (mRNA and protein) correlated with poor prognosis in LUAD (Figure S9I), but showed no significant association in lung squamous cell carcinoma (Figure S9J, Supporting Information). Similarly, in *KRAS*‐mutant PAAD, elevated *E2F1* expression predicted poor outcomes at both transcript and protein levels (Figure S9K, Supporting Information), suggesting *KRAS*‐driven *COA4* upregulation via E2F1 may operate across multiple malignancies.

Functionally, overexpression of *E2F1* enhanced cellular ATP production, whereas *COA4* knockdown significantly reduced ATP levels in this context (Figure 5J). Similarly, *COA4* knockdown reversed the increase in COX activity induced by *E2F1* overexpression (Figure 5K). To further clarify the relationship between E2F1/COA4 expression and mitochondrial function in LUAD, we stratified patient samples based on high or low expression of both genes. Tumors with high expression of both *E2F1* and *COA4* exhibited significantly higher OXPHOS scores and COX activity scores compared to tumors with low expression of both, or those with high *E2F1* but low *COA4* (Figure S9L), demonstrating a direct association between their expression and mitochondrial oxidative capacity.

Moreover, overexpression of *KRAS* ^G12C^ or *KRAS* ^G12D^ enhanced COA4 mRNA and protein levels, an effect that was partially inhibited by *E2F1* knockdown (Figure 5L,M and Figure S9M, Supporting Information). Furthermore, treatment with a PI3K agonist increased *COA4* expression, whereas *E2F1* knockdown partially attenuated the PI3K activation‐induced upregulation of COA4 (Figure 5N). Conversely, *E2F1* overexpression partially rescued the decrease in *COA4* expression caused by a PI3K inhibitor, with similar results observed at the mRNA level (Figure 5O,P and Figure S9N, Supporting Information). Critically, we identified a physical interaction between AKT, a key downstream effector of PI3K, and E2F1, suggesting E2F1 acts as a downstream mediator of PI3K signaling to regulate *COA4* expression (Figure S9O, Supporting Information).

Functionally, *E2F1* knockdown significantly impaired the invasion and migration of LUAD cells; these effects were partially rescued by *COA4* overexpression. Conversely, *E2F1* overexpression promoted cell invasion and migration, an effect partially inhibited by *COA4* knockdown (Figure 5Q). Furthermore, treatment with a PI3K agonist significantly enhanced cell invasion and migration, and this enhancement was partially reversed by *E2F1* knockdown. Similarly, a PI3K inhibitor suppressed invasion and migration, an effect partially rescued by *E2F1* overexpression (Figure 5R and Figure S9P, Supporting Information). Additionally, *E2F1* knockdown partially inhibited the enhanced invasiveness and migratory capacity induced by *KRAS* ^G12D^ overexpression in LUAD cells, and subsequent *COA4* overexpression partially restored these functions (Figure 5S and Figure S9Q, Supporting Information).

Analysis of TCGA data revealed significantly higher *E2F1* expression in *KRAS*‐mutant lung cancer patients compared to those with wild‐type *KRAS* (Figure 5T). Consistent with this, analysis of GEO datasets indicated significantly elevated *E2F1* expression in LUAD tissues with high *KRAS* expression relative to those with low *KRAS* expression (Figure 5T).

Finally, analysis of LUAD GEO datasets demonstrated that high co‐expression of *COA4* and *E2F1* is significantly associated with poor prognosis (Figure 5U). Combined analysis using the R package linkET further revealed that higher combined expression levels of *COA4* and *E2F1* correlate with increased mortality and shorter overall survival (Figure 5V).

Collectively, these results demonstrate that E2F1 functions as an oncogenic transcription factor in LUAD by directly binding to the *COA4* promoter and activating its transcription. This regulatory axis is governed upstream by the KRAS/PI3K signaling pathway, ultimately enhancing mitochondrial OXPHOS and thereby promoting tumor invasion and migration.

### 2.6 *COA4* Interacts with and Activates CDC42 to Drive Lung Tumorigenesis

In addition to its mitochondrial localization, *COA4* was observed throughout the cytoplasm. Notably, *COA4*‐GFP overexpression revealed accumulation at the cell membrane, where it induced pseudopodia‐like protrusions (Figure 1B). This distribution suggested potential cytoplasmic roles in LUAD metastasis. To investigate this, we examined COA4‐mediated regulation of tumor cell migration through cytoplasmic protein interactions.

**Figure 6:**
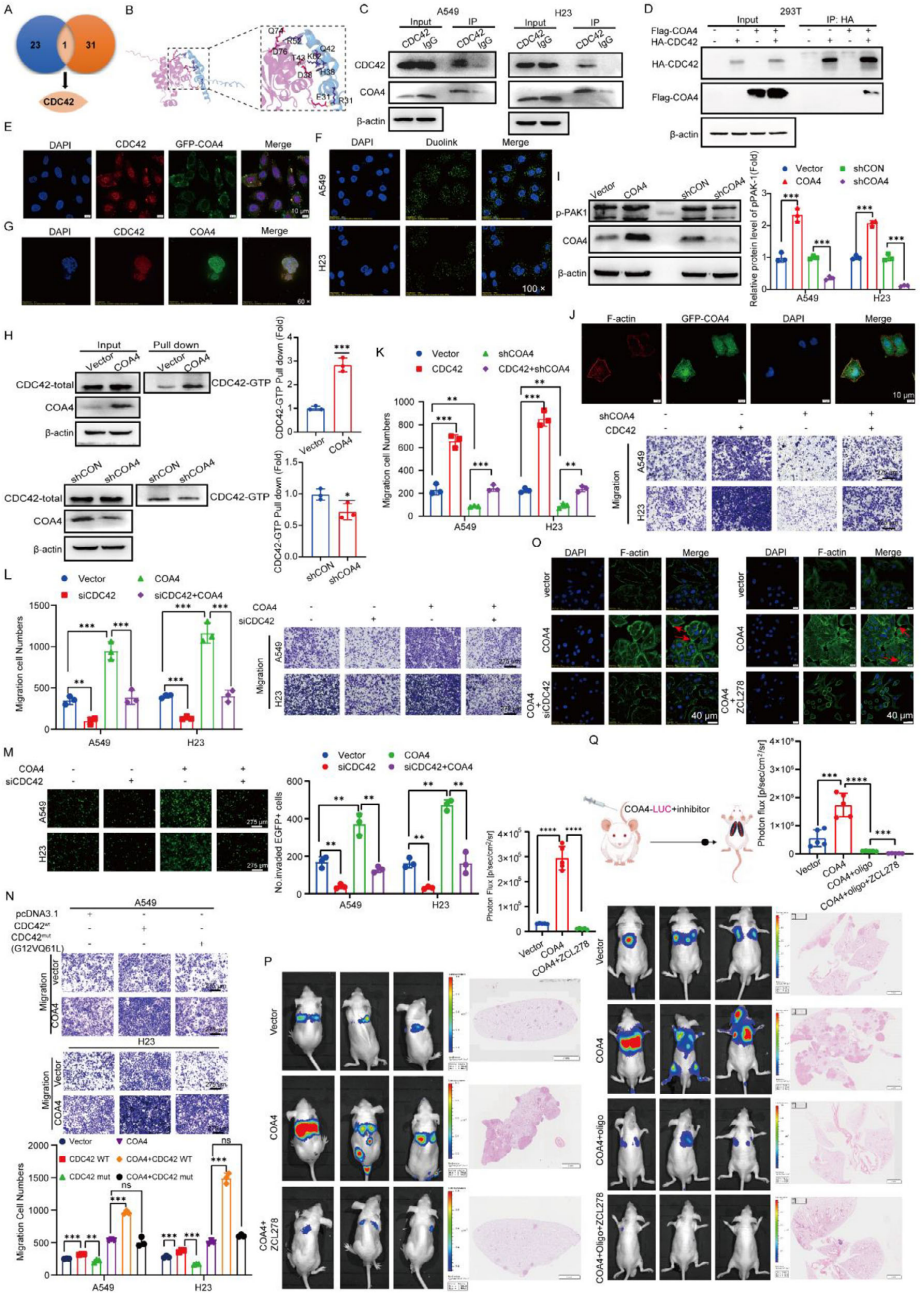
COA4 interacts with and activates CDC42 to drive lung tumorigenesis. A) Identification of COA4‐interacting proteins in A549 cells following COA4‐Flag transfection, using IP and mass spectrometry (MS), cross‐referenced with the NCBI Gene database. B) AlphaFold 3 molecular docking analysis of the 3D structures, revealing the interaction between COA4 and CDC42. C) Endogenous Co‐IP assays in A549 and H23 cells confirming the interaction between COA4 and CDC42.(*n* = 3) D. Exogenous Co‐IP in 293T cells validating the binding between COA4 and CDC42. E) IF analysis demonstrating colocalization of CDC42 (red) and COA4‐GFP in A549 cells, with nuclei counterstained with DAPI. Scale bar: 40 µm (n=3) F) PLA visualizing the interaction between CDC42 and COA4 in A549 and H23 cells. Scale bar: 20 µm (*n* = 3) G) IF analysis showing colocalization of CDC42 (red) and COA4 (green) in LUAD PDOs. Scale bar: 40 µm (*n* = 3) H) Analysis of CDC42‐GTP activity in A549 cells following COA4 knockdown or overexpression. (*n* = 3; *p < 0.05; ***p < 0.001) I) Western blot analysis of phosphorylated PAK1 (p‐PAK1) levels in A549 cells with COA4 knockdown or overexpression. (*n* = 3; ***p < 0.001) J) IF analysis revealing colocalization of COA4 with F‐actin in filopodia‐like structures. Scale bar: 10 µm (*n* = 3) K,L) Transwell assays evaluating migration in A549 and H23 cells: K) in *COA4*‐knockdown cells following *CDC42* overexpression, and L) in *COA4*‐overexpressing cells following *CDC42* knockdown. Scale bar: 275 µm (*n* = 3; **p < 0.01; ***p < 0.001) M) Trans‐endothelial migration assays assessing the migration of *COA4*‐overexpressing A549 and H23 cells after *CDC42* knockdown. Scale bar: 275 µm (*n* = 3; **p < 0.01) N) Transwell assays comparing the migration abilities of *COA4*‐overexpressing A549 and H23 cells transfected with either pcDNA3.1 (control), wild‐type *CDC42* (*CDC42* ^wt^), or the inactive mutant *CDC42* ^G12V/Q61L^. Scale bar: 275 µm (*n* = 3; ns, not significant; **p < 0.01; ***p < 0.001) O) IF analysis of F‐actin filopodia‐like structures in *COA4*‐overexpressing A549 cells following *CDC42* knockdown (left) or treatment with the CDC42 inhibitor ZCL278 (right). Scale bar: 40 µm (*n* = 3) P) In vivo metastasis analysis following tail vein injection of luciferase‐tagged A549 cells stably overexpressing *COA4*, with subsequent intraperitoneal injection of ZCL278. Metastatic burden was assessed by measuring fluorescence intensity, supported by H&E staining and statistical analysis (*n* = 5 per group, ****p < 0.0001). Q) In vivo metastasis analysis following tail vein injection of luciferase‐tagged A549 cells overexpressing *COA4*, with subsequent treatment using oligomycin in combination with ZCL278. Metastatic burden was evaluated via fluorescence intensity measurements, H&E staining, and statistical analysis (*n* = 5 per group, ***p < 0.001; ****p < 0.0001). The data are given as mean ± SD and compared by two‐tailed unpaired Student's *t*‐test (H, I, K‐Q). Significance levels are indicated as follows: ns, not significant; *p < 0.05; **p < 0.01; ***p < 0.001; ****p < 0.0001.

Integration of our proteomics data with NCBI‐CDC42 annotations (Gene ID: 51287) identified CDC42 as a putative COA4 interactor (**Figure** **6**A). Given CDC42's established role in tumor cell migration,^[^ ^27^ ^]^ we performed in silico docking using AlphaFold 3, predicting direct COA4‐CDC42 binding (Figure 6B). Endogenous co‐immunoprecipitation (Co‐IP) in A549 and H23 cells confirmed this interaction (Figure 6C). In 293T cells, co‐expression of Flag‐*COA4* and HA‐*CDC42* further validated binding (Figure 6D and Figure S10A, Supporting Information), while IF demonstrated cytoplasmic co‐localization (Figure 6E). PLA in A549/H23 cells (Figure 6F) and co‐localization studies in LUAD organoids (Figure 6G) provided additional in situ confirmation.

To determine whether COA4 regulates tumor cell migration through CDC42, we assessed the impact of altered COA4 expression on CDC42 protein levels and activity. While manipulation of *COA4* did not significantly alter total CDC42 protein levels (Figure 6H), *COA4* knockdown reduced CDC42 GTPase activity, whereas *COA4* overexpression enhanced it (Figure 6H). CDC42 activity is finely orchestrated through multi‐tiered regulatory mechanisms^[^ ^28^ ^]^: Guanine nucleotide exchange factors (GEFs) activate CDC42 by catalyzing GDP‐to‐GTP exchange, while GTPase‐activating proteins (GAPs) terminate signaling through accelerated GTP hydrolysis. Scaffold proteins further confer spatiotemporal precision to CDC42 signaling by orchestrating its subcellular localization and facilitating assembly of signalosomes with specific downstream effectors. To elucidate the molecular mechanism by which COA4 regulates CDC42 activity, we analyzed the COA4 interactome via mass spectrometry and identified proteins whose expression was altered upon COA4 knockdown. Notably, COA4 potentially interacts with TRIP10, ITSN1, and IQGAP1—key regulators of CDC42 activity—while ABR (another CDC42 activity modulator) exhibited COA4‐dependent expression changes. These proteomic data suggest that COA4 may activate CDC42 by modulating CDC42‐associated GEFs, GAPs, or scaffold proteins. To define this mechanism, we first examined the interaction and subcellular localization of canonical CDC42 regulators (ARHGAP1) and the aforementioned proteins in COA4‐modulated cells. Strikingly, COA4 overexpression significantly enhanced the CDC42‐ITSN1 interaction and pseudopodia formation, whereas COA4 knockdown suppressed CDC42‐ITSN1 binding (Figure S10B, Supporting Information). Co‐immunoprecipitation assays further confirmed that COA4 expression positively regulates the CDC42‐ITSN1 interaction (Figure S10C, Supporting Information). Critically, COA4 modulation did not alter ITSN1 protein levels (Figure S10C, Supporting Information). Collectively, these results indicate that COA4 binding to CDC42 promotes ITSN1‐CDC42 complex assembly, thereby activating CDC42 signaling. Since PAK1 is a key downstream effector of CDC42,^[^ ^29^ ^]^ we found that *COA4* knockdown markedly decreased PAK1 phosphorylation, while *COA4* overexpression increased it (Figure 6I and Figure S10D, Supporting Information). Given the established role of CDC42 activation in invasive pseudopodia formation^[^ ^30^ ^]^, we examined pseudopodial dynamics and observed that *COA4*‐GFP overexpression induced F‐actin‐enriched pseudopodia at the cell periphery (Figure 6J). These data demonstrate that COA4 promotes invasion by directly interacting with and activating CDC42.

We next evaluated CDC42's functional role in COA4‐driven migration and invasion. In A549 and H23 cells, CDC42 overexpression enhanced invasion/migration, whereas *COA4* knockdown in CDC42‐overexpressing cells partially attenuated these phenotypes (Figure 6K and Figure S10E, Supporting Information). Conversely, CDC42 knockdown significantly impaired the *COA4* overexpression‐induced increases in invasion and migration (Figure 6L and Figure S10F, Supporting Information). Furthermore, CDC42 depletion reduced the ability of *COA4*‐overexpressing cells to traverse endothelial layers (Figure 6M). Mutagenesis of key CDC42 enzymatic residues (Gly^12^, Gln^61^) demonstrated that wild‐type *CDC42* overexpression further enhanced invasion/migration in *COA4*‐overexpressing cells, whereas dominant‐negative CDC42 mutants failed to augment these effects (Figure 6N and Figure S10G, Supporting Information). These findings confirm that COA4 requires CDC42 GTPase activity to exert oncogenic functions. Treatment with the CDC42 inhibitor ZCL278 significantly suppressed COA4‐mediated invasion, migration (Figure S10H, Supporting Information), and trans‐endothelial migration (Figure S10I). Both CDC42 knockdown and ZCL278 treatment inhibited COA4‐induced F‐actin reorganization and pseudopodia formation (Figure 6O and Figure S11A, Supporting Information). In vivo, ZCL278 reduced lung metastasis of *COA4*‐overexpressing A549 cells (Figure 6P).

Given the established role of COA4 in promoting mitochondrial OXPHOS via COX regulation, we evaluated combinatorial targeting of COX and CDC42. Dual inhibition significantly reduced F‐actin expression, disrupted cytoskeletal organization, and suppressed pseudopodia formation in *COA4*‐overexpressing cells (Figure S11B, Supporting Information). Furthermore, this combination attenuated FAK phosphorylation (Figure S11C, Supporting Information), inhibited in vitro invasion/migration (Figure S11D), and impaired single‐cell motility in time‐lapse assays (Figure S11E, Supporting Information). In vivo, combined treatment suppressed COA4‐driven lung metastasis (Figure S11F, Supporting Information). Critically, simultaneous OXPHOS and CDC42 inhibition also significantly reduced COA4‐mediated metastasis (Figure 6Q).

Collectively, these results demonstrate that COA4 drives lung tumor progression through two synergistic mechanisms: 1) regulating mitochondrial OXPHOS and 2) directly activating CDC42. This dual signaling promotes invasive pseudopodia formation, facilitating metastasis. Therapeutic co‐targeting of CDC42 and mitochondrial respiration (via COX or OXPHOS inhibition) represents a promising strategy to counteract COA4‐mediated metastasis in LUAD.

### 2.7 *Saccharomyces cerevisiae*‐Derived *COA4* Possesses a Cancer‐Promoting Function in LUAD Cells

Thousands of human genes possess clear homologs in yeast, all derived from a common ancestor approximately one billion years ago, and many exhibit conserved functions.^[^ ^31^ ^]^ For example, the mitochondrial gene *NDI1* (NADH‐ ubiquinone oxidoreductase) from *S. cerevisiae* can be functionally expressed in mammalian cells, where it performs normal physiological roles. In fact, overexpression of yeast‐derived *NDI1* in mammalian cells has been employed as a therapeutic strategy to rescue mitochondrial dysfunction caused by rotenone inhibition or mutations in mammalian complex I genes.^[^ ^32^ ^]^ Martínez‐Reyes et al. demonstrated that *NDI1* overexpression can compensate for mitochondrial complex I deficiencies, thereby promoting tumor cell growth.^[^ ^14^ ^]^ Notably, previous studies have reported that COA4 participates in regulating mitochondrial COX activity of *S. cerevisiae*. Our own data show that human COA4 similarly modulates COX activity in human cell lines, prompting us to investigate whether *COA4* is evolutionarily conserved across species.

**Figure 7:**
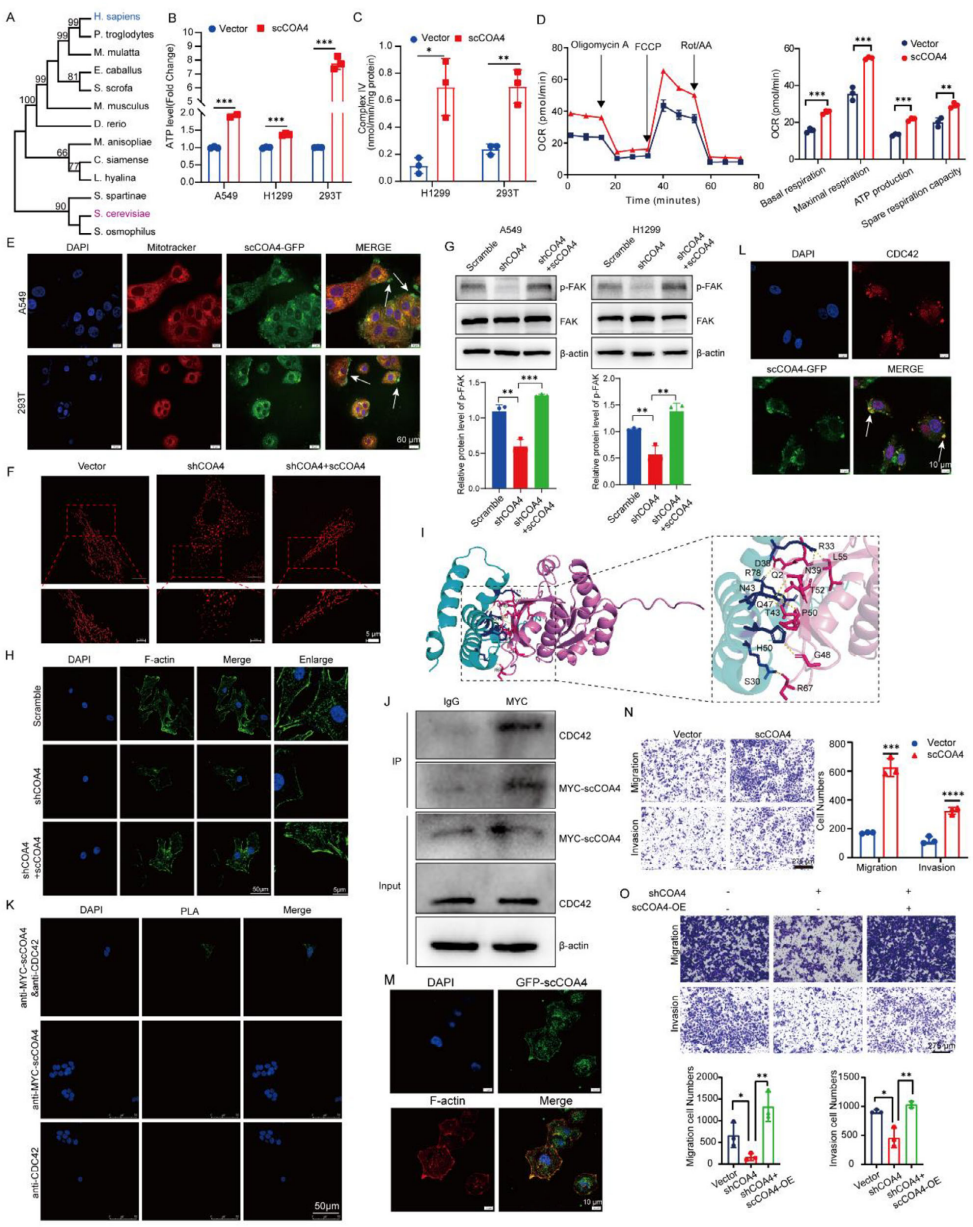
*Saccharomyces cerevisiae*‐derived *COA4* has a cancer‐promoting function in LUAD cells. A) Phylogenetic analysis of *COA4* homologs across multiple species. B) Analysis of intracellular ATP levels in A549, H1299, and 293T cells transfected with *S. cerevisiae*‐derived *COA4* (*scCOA4*). (*n* = 3; ***p < 0.001) C) Assessment of Complex IV (cytochrome c oxidase) activity in H1299 and 293T cells transfected with *scCOA4*. (*n* = 3; *p < 0.05; **p < 0.01) D) OCR analysis in A549 cells transfected with *scCOA4*, measured using the Seahorse XFe96 system. OXPHOS parameters—including basal respiration, maximal respiration, ATP production, and spare respiratory capacity—were evaluated. Quantification of basal respiration, maximal respiration, ATP production, and spare capacity according to instruction (*n* = 3; **p < 0.01, ***p < 0.001). E) IF analysis of scCOA4 (green) in 293T and A549 cells, with nuclei (DAPI, blue) and mitochondria (MitoTracker Red CMXRos, red) labeled. Scale bar: 50 µm (*n* = 3) F) SIM imaging of mitochondria in *COA4*‐knockdown A549 cells rescued by *scCOA4* re‐expression, visualized with MitoTracker Red CMXRos. Scale bar: 5 µm (*n* = 12 cells each). G) Western blot analysis of phosphorylated FAK (p‐FAK) levels in *COA4*‐knockdown A549 cells following scCOA4 re‐expression. (*n* = 3; **p < 0.01; ***p < 0.001) H) IF analysis of F‐actin filopodia‐like structures in *COA4*‐knockdown A549 cells after *scCOA4* re‐expression. Scale bar: 50 µm (*n* = 3) I. AlphaFold 3 molecular docking analysis demonstrating the 3D interaction between scCOA4 and CDC42. J) IP analysis in A549 cells transfected with *scCOA4*‐MYC, confirming its interaction with CDC42.(*n* = 3) K) PLA visualizing the interaction between scCOA4 and CDC42 in A549 cells.(*n* = 3) L) IF analysis showing colocalization of CDC42 (red) and scCOA4‐GFP in A549 cells.(*n* = 3) M) IF demonstrating colocalization of scCOA4‐GFP with F‐actin in A549 cells.(*n* = 3) N) Transwell assays evaluating the migration and invasion capabilities of A549 cells transfected with *scCOA4*.(*n* = 3; ***p < 0.001;****P < 0.0001) O) Transwell assays analyzing the migration and invasion abilities of *COA4*‐knockdown A549 cells rescued by *scCOA4* re‐expression.(*n* = 3; *p < 0.05; **p < 0.01) The data are given as mean ± SD and compared by Student's *t*‐test B–D, F–H, K, N, and O) and one‐way ANOVA D). Significance levels are indicated as follows: ns, not significant; *p < 0.05; **p < 0.01; ***p < 0.001; ****p < 0.0001.

Phylogenetic analysis revealed that *COA4* homologs exist in multiple species. Human *COA4* emerged relatively late in evolution, suggesting this gene originated in earlier organisms such as *S. cerevisiae* and *S. osmophilus* (**Figure** **7**A). We then tested whether *S. cerevisiae*‐derived *COA4* (*scCOA4*) can be functionally expressed in human cells and exert its normal physiological functions. Overexpression of *scCOA4* in 293T, A549, and H1299 cells significantly increased intracellular ATP levels (Figure 7B). Furthermore, *scCOA4* overexpression markedly enhanced COX activity (Figure 7C). Using Seahorse assays, we observed significant increases in mitochondrial oxidative respiration parameters, specifically basal respiration, maximal respiration, and ATP production (Figure 7D).

IF analyses in 293T and A549 cells revealed that the subcellular localization of scCOA4 closely mirrors that of human COA4 protein: it localizes to mitochondria, the nucleus, and the cytoplasm, and also forms pseudopodia‐like structures at the cell membrane (Figure 7E). Importantly, *scCOA4* overexpression partially rescued mitochondrial fragmentation induced by endogenous *COA4* knockdown (Figure 7F and Figure S12A, Supporting Information), suggesting *scCOA4* can fulfill essential physiological roles in mammalian cells. We further hypothesized that *scCOA4* might regulate FAK phosphorylation and F‐actin distribution similarly to human *COA4*. Indeed, *scCOA4* overexpression partially restored the decreased phospho‐FAK levels caused by *COA4* knockdown (Figure 7G) and recovered F‐actin distribution and expression (Figure 7H and Figure S12B, Supporting Information), demonstrating functional conservation between yeast and human *COA4*.

Given that CDC42 is a conserved protein initially identified in *S. cerevisiae* and retains similar functions in mammalian cells,^[^ ^33^ ^]^ we speculated that yeast‐derived COA4 might interact with human CDC42. Remarkably, AlphaFold 3 analysis revealed that despite differences in the three‐dimensional structures of human COA4 (hCOA4) and scCOA4, the yeast protein interacts with CDC42 (Figure 7I). Co‐IP (Figure 7J) and PLA (Figure 7K) experiments further confirmed the scCOA4‐CDC42 interaction. IF studies demonstrated that scCOA4‐GFP colocalizes with CDC42 and, consistent with hCOA4, accumulates in pseudopodia‐like structures at the cell membrane (Figure 7L). Moreover, *scCOA4* overexpression reversed the reduction in p‐PAK1 levels caused by *COA4* knockdown (Figure S12C, Supporting Information). Additional IF analyses confirmed that GFP‐scCOA4 colocalizes with F‐actin within pseudopodia, exhibiting prominent accumulation at these sites (Figure 7M), demonstrating conserved functional interactions across species.

To further delineate the role of *scCOA4* in LUAD cell invasion and migration, we overexpressed *scCOA4* in multiple LUAD cell lines. *scCOA4* overexpression significantly promoted invasion and migration in all lines tested (Figure 7N and Figure S12D, Supporting Information) and partially restored the impaired invasive and migratory capacity caused by endogenous *COA4* knockdown (Figure 7O). Notably, treatment with OXPHOS or COX inhibitors suppressed invasion and migration; however, *scCOA4* overexpression partially rescued these phenotypes in LUAD cells (Figure S12E, Supporting Information). Furthermore, in pancreatic cancer cells, *scCOA4* overexpression partially reversed the loss of invasion and migration following *COA4* knockdown (Figure S12F, Supporting Information). Sequence alignment revealed that both *scCOA4* and human *COA4* harbor two conserved CX9C motifs. Upon mutating these motifs in *scCOA4*, the mutant *scCOA4* failed to rescue the impaired invasion and migration caused by *COA4* knockout (Figure S12G, Supporting Information).

These findings indicate that the scCOA4 gene has retained key amino acid sequences through cross‐species evolution, preserving its molecular function. In human cells, *scCOA4* exhibits physiological regulatory functions similar to those of human *COA4*, contributing to the invasion and migration of LUAD cells.

## 3 Discussion

For decades, the Warburg effect has been considered a central metabolic hallmark of tumors. However, recent evidence challenges this paradigm, as OXPHOS is elevated—not suppressed—in LUAD cells.^[^ ^13^ ^]^ Although enhanced mitochondrial OXPHOS drives progression in many solid tumors, its regulatory mechanisms remain incompletely understood. Although *COA4* was initially identified in *S. cerevisiae* as a key regulator of mitochondrial COX activity, its role in mammalian systems remained uncharacterized. In our study, we demonstrate for the first time that COA4 localizes to the cytoplasm, nucleus, and mitochondria. Mechanistically, COA4 regulates COX activity via two distinct pathways: by modulating the transcription of nuclear‐encoded COX subunits and by affecting the protein stability of the mitochondrially encoded MTCO1. Thus, COA4 emerges as a novel regulator of mitochondrial homeostasis, linking COX activity to OXPHOS efficiency. Moreover, we identified a physical interaction between COA4 and SLC25A3—the primary copper transporter regulating COX activity. Functional studies demonstrated that genetic perturbation of *COA4* disrupts mitochondrial copper flux, indicating that COA4 coordinately regulates copper homeostasis through complex formation with SLC25A3, thereby maintaining mitochondrial integrity.

It is well documented that many human genes have clear homologs in yeast, originating from a common ancestor approximately one billion years ago, and often exhibit conserved functions. For instance, *S. cerevisiae*‐derived *NDI1* (NADH‐ubiquinone oxidoreductase) can be functionally expressed in mammalian cells and maintain its physiological activity.^[^ ^32^ ^]^ Consistent with this, our findings indicate that yeast‐derived *COA4* can be expressed in human cells, displaying a subcellular localization pattern similar to that of its human counterpart. Functionally, yeast *COA4* is capable of regulating mitochondrial COX activity and OXPHOS in human cells and even interacts with human CDC42 to facilitate tumor cell metastasis, underscoring its evolutionary conservation.

Our data also reveal that *COA4* is highly expressed in various tumor cells. In LUAD, high *COA4* expression correlates with poor prognosis, making it a potential clinical prognostic marker. Both in vitro and in vivo experiments demonstrate that COA4 enhances LUAD cell trans‐endothelial migration, extravasation, invasion, and metastasis. These findings position *COA4* as a novel oncogene driving LUAD metastasis. Moreover, knockdown of *COA4* or pharmacological inhibition of COX or OXPHOS significantly suppresses tumor cell invasion and migration, suggesting that COA4 promotes metastasis by enhancing mitochondrial oxidative metabolism. Interestingly, GFP‐tagged COA4 accumulates at the cell membrane, forming pseudopodia‐like structures—key cellular protrusions that facilitate invasion and migration—hinting at a possible additional role for COA4 in pseudopodia formation that merits further investigation.

Notably, COA4 does not appear to affect anchorage‐dependent or ‐independent proliferation of LUAD cells, although it does influence organoid formation and growth. Similar observations have been reported for COX6B2, another family member of COX, which selectively impacts PDAC cell invasion and migration without affecting proliferation.^[^ ^15a^ ^]^ Critically, whether *COX6B2*—like *COA4*—is transcriptionally regulated by KRAS and thereby promotes tumor metastasis remains unknown. Furthermore, although COX6B2 has been reported to drive PAAD progression by enhancing mitochondrial OXPHOS,^[^ ^15a^ ^]^ its potential oncogenic functions in extra‐mitochondrial compartments (e.g., cytosol) parallel to COA4 await mechanistic exploration.

Additionally, under hypoxic or nutrient‐deprived conditions, modulation of COA4 does not alter LUAD cell proliferation, although its in vivo effects on tumor growth through modulation of the immune microenvironment cannot be ruled out.

*KRAS* mutations represent one of the most common oncogenic drivers in LUAD, pancreatic adenocarcinoma, and other malignancies, yet therapeutic options directly targeting KRAS remain limited and prone to resistance. Consequently, identifying and targeting downstream effectors of KRAS is an attractive strategy. Prior studies have demonstrated that *KRAS*‐driven lung tumors exhibit increased glucose flux into the tricarboxylic acid (TCA) cycle, with mitochondrial pyruvate metabolism being essential for tumor formation in vivo.^[^ ^35^ ^]^ In our study, we show that *KRAS* drives *COA4* expression via the PI3K pathway, and that *COA4* is markedly overexpressed in LUAD tissues with *KRAS* mutations or high *KRAS* expression. In multiple *KRAS*‐mutant tumor cell lines, *COA4* is consistently upregulated, and its knockdown partially reverses KRAS‐induced invasion and migration, establishing COA4 as a critical downstream effector of *KRAS* in promoting tumor metastasis. Moreover, our in vitro and in vivo findings in pancreatic adenocarcinoma—a tumor type with one of the highest *KRAS* mutation rates—further indicate that *COA4* knockdown suppresses metastasis. These results suggest that COA4 is a common downstream mediator of metastasis in *KRAS*‐driven tumors. Interestingly, *COA4* is also highly expressed in some*KRAS*‐wildtype cells, and its modulation similarly affects invasion and migration, implying the existence of additional upstream regulatory factors besides *KRAS*. These findings underscore the therapeutic potential of targeting *COA4* not only in *KRAS*‐mutant LUAD but also in other tumor subtypes.

We further identified E2F1 as a direct transcriptional regulator mediating *KRAS*‐driven *COA4* expression. E2F1 binds directly to the *COA4* promoter, and its expression is positively correlated with that of *COA4*. Overexpression of *E2F1* enhances tumor cell invasion and migration, whereas *COA4* knockdown mitigates the malignant phenotypes induced by E2F1. Moreover, higher combined expression levels of *E2F1* and *COA4* are associated with increased OXPHOS activity, elevated COX activity scores, and poorer clinical outcomes. These findings establish that E2F1, regulated by the KRAS/PI3K axis, plays a crucial role in promoting *COA4* expression and thereby contributes to LUAD invasion and metastasis. Notably, our data reveal that modulation of *COA4* expression does not significantly impact LUAD cell proliferation, suggesting that compensatory oncogenic pathways downstream of *KRAS*—such as RAF‐MEK‐ERK, JAK‐STAT, and RAL signaling cascades—may dominantly drive proliferative processes in this malignancy.

In addition to its mitochondrial localization, COA4 is also present in the cytoplasm, where we discovered that it interacts with CDC42—a key regulator of cytoskeletal organization, cell‐to‐cell adhesion, pseudopodia activation, and protrusion formation.^[^ ^36^ ^]^ Our data indicate that COA4 directly interacts with and activates CDC42, rather than affecting its protein stability, to promote LUAD cell invasion and migration. Consistent with this, both genetic knockdown and pharmacological inhibition of CDC42 significantly reduce the invasive and migratory capabilities induced by *COA4* overexpression. Precise delineation of the key functional domain(s) within COA4 responsible for binding and activating CDC42 is crucial for elucidating the structural basis of COA4‐mediated CDC42 activation and for developing targeted therapeutic strategies to disrupt this interaction. However, persistent failure to achieve stable expression of truncated COA4 variants in our domain mapping analyses precluded definitive identification of the specific domain(s) mediating the COA4‐CDC42 interaction. Consequently, future studies will focus on elucidating the underlying mechanisms impairing truncated COA4 expression, precisely mapping the essential CDC42‐binding domain(s), and screening for small‐molecule compounds capable of selectively targeting this interface to therapeutically disrupt the COA4‐CDC42 axis. Notably, CDC42 was initially identified in *S. cerevisiae* and its function is evolutionarily conserved in mammalian cells.^[^ ^33^ ^]^ We further demonstrate that yeast‐derived COA4 interacts with CDC42 in human cells, emphasizing the evolutionary conservation of COA4's function. Given that other microbial‐derived genes, such as bacterial *AARS1/2*, have also been shown to retain conserved functions in human cells and contribute to tumorigenesis,^[^ ^37^ ^]^ it is tempting to speculate that genes from yeast or bacteria, which maintain physiological functions in mammalian cells, may be particularly susceptible to dysregulation in disease states. However, this hypothesis warrants further comprehensive investigation.

Building on our mechanistic insights, future clinical strategies for LUAD patients with *KRAS* mutations or high *COA4* expression should prioritize dual blockade of OXPHOS and CDC42 signaling. Concurrently, screening for small‐molecule degraders or inhibitors of COA4 represents a promising therapeutic avenue for this molecularly defined cohort. Notably, while COA4 constitutes a compelling target, systemic inhibition of the COA4‐OXPHOS‐CDC42 axis risks on‐target toxicity in healthy tissues due to conserved physiological functions. To circumvent this challenge, we propose: (i) tumor‐selective delivery platforms enabling spatiotemporal precision, or (ii) repurposing clinically validated agents with established safety profiles—such as OXPHOS inhibitors (CPI‐613),^[^ ^38^ ^]^ canonical CDC42 inhibitors (MBQ‐167),^[^ ^39^ ^]^ and recently developed CDC42 antagonists (Daphnepedunin A).^[^ ^40^ ^]^

Although combinatorial OXPHOS/CDC42 inhibition exhibited minimal cytotoxicity in vitro, comprehensive toxicological profiling in preclinical models remains imperative prior to clinical translation. In this study, we did not address whether combined inhibition of oxidative phosphorylation and CDC42 affects metastasis in COA4‐null tumor cells. This aspect was not explored in detail due to the high baseline COA4 expression levels in the currently utilized cell lines and transgenic mouse models. Therefore, to resolve this question, future studies will utilize PDOs established from COA4‐null clinical tumor specimens. These investigations will specifically assess the therapeutic vulnerability of COA4‐null tumors to this combinatorial regimen and determine whether COA4 deficiency intrinsically confers refractoriness to metastatic dissemination.

In summary, our study identifies COA4 as a novel regulator of mitochondrial COX activity and OXPHOS homeostasis with evolutionarily conserved functions. We demonstrate that the KRAS/PI3K/E2F1 signaling axis drives *COA4* expression, which in turn contributes to LUAD progression by reprogramming mitochondrial metabolism and by activating CDC42 to promote metastasis. Importantly, combined inhibition of OXPHOS, COX, and CDC42 markedly suppresses COA4‐induced metastasis. These findings establish *COA4* as a pro‐metastatic oncogene in *KRAS*‐driven LUAD and PDAC, highlighting its potential as a therapeutic target for clinical intervention.

## 4 Experimental Section

### Patients and Specimens

Human LUAD and paired adjacent normal tissues were obtained from patients undergoing surgical resection at the Provincial Hospital of Shandong First Medical University. A human LUAD tissue microarray (Shanghai Outdo Biotech) comprising specimens from 75 patients who underwent curative resection was used. Our study adheres to the guidelines outlined in the Declaration of Helsinki and has received approval from the Ethics Committee of Shandong Provincial Hospital Affiliated to Shandong First Medical University.

### Cell Lines and Cell Culture

A549, H1299, H23, H460, H1975, HCC827, BEAS‐2B, HUVEC, and HEK293T cell lines were obtained from Procell Life Science & Technology Co., Ltd. A549 cells were cultured in F12K medium (Gibco) with 10% FBS and antibiotics (100 U mL^−1^ penicillin, 100 µg mL^−1^ streptomycin), while BEAS‐2B, H23, H1299, H1975, HCC827, and HEK293T were maintained in RPMI‐1640 medium with the same supplementation. HUVEC cells were grown in HUVEC‐specific medium (Procell). All cultures were maintained at 37 °C, with 5% CO_2_. The following inhibitors were used: LY294002 (20 µM, 48 h, PI3K), Stattic (5–15 µm, 48 h, STAT3), BQU57 (2–6 µm, 48 h, Ral GTPase), PD0325901 (5–20 µm, 48 h, MEK), Oligomycin A (1 µm, 24 h, H⁺‐ATP synthase), ZCL278 (25 µm, 24 h, CDC42), and (NH_4_)_2_MoS_4_ (100 µM, 24 h, Complex IV), Doxycycline (2.25 µm, 0–48 h, mitochondrial ribosome inhibitor). The following agonist was used: 740Y‐P (15 µm, 48 h, PI3K). All reagents were from Selleck or MCE (Shanghai, China).

### Plasmids, siRNAs, Lentiviral Infection, and Transfection

Overexpression plasmids for *E2F1*, *CDC42*, *CDC42* ^G12V/Q61L^, *KRAS* ^G12C^, and *KRAS* ^G12D^ were obtained from Genomeditech (Shanghai). Full‐length *COA4* and *COA4* mutant plasmids were constructed by BioSune (Shanghai). Small interfering RNAs (siRNAs) targeting *E2F1*, *CDC42*, *FAK*, and *KRAS* were synthesized by Sangon Biotech (Shanghai). Transient plasmid transfection was performed using Lipofectamine 3000 (Invitrogen, L3000075) according to the manufacturer's protocol. For siRNA transfections, RNATransMate (Sangon Biotech, E607402) was used per the manufacturer's instructions.

Lentiviruses encoding *COA4* shRNA or *COA4* construct were obtained from Genomeditech (Shanghai). LUAD cells at 40% confluency were infected with lentiviral particles in the presence of 5 µg mL^−1^ polybrene. After 48–72 h, the medium was replaced with complete medium containing 1.5µg mL^−1^ puromycin.

### CRISPR‐Cas9‐Mediated Gene Knockout *COA4*

The CRISPR/Cas9‐*COA4* knockout (KO) construct, encoding the sgRNA specifically targeting COA4, was produced by Weizhen (Jinan, China). The sequences were as follows: COA4‐gRNA1: TCACCCGTTGGGTCCAGGTA; COA4‐gRNA2: GCATCGTCTTTCTTCACCCGT; COA4‐gRNA3: GACCAGCTGATCTCCCGCTCT.

CRISPR lentivirus was generated by co‐transfecting 10 µg lenti‐CRISPR gRNA or lenti‐CRISPR CTRL with pMD2.G and psPAX2 (500 ng:250 ng:250 ng) into HEK293T cells using Lipofectamine 3000. After 72 h, viral supernatants were collected, filtered (0.22 µm), and used to transduce LUAD cells and patient‐derived organoids (PDOs). Transfected cells were selected with 1.5 µg mL^−1^ puromycin.

### Xenograft Mouse Models of Lung Cancer

Nude male BALB/c mice (4–6 weeks, Beijing Vital River) were housed in specific pathogen‐free facilities. For xenograft assays, A549 cells (5 × 10⁶ in a PBS‐Matrigel mixture, 100 µL) were subcutaneously injected into the left axilla. Tumor volumes were measured weekly for 28 days. Mice were euthanized at week 4, and tumor volume/weight was analyzed. In all animal experiments, the investigators responsible for outcome assessment and data analysis were blinded to the group allocations.

### In Vivo Metastasis Assay Design and Statistical Justification

For in vivo metastasis assays, mice were randomly allocated into experimental groups by an independent investigator to minimize allocation bias. To reduce procedural bias, drug administration, in vivo bioluminescence imaging, and tissue collection were each performed by separate, designated experimenters. Crucially, investigators responsible for data analysis and phenotypic evaluation remained blinded to group assignments throughout the study.

The sample size was determined based on ethical considerations, logistical feasibility, and the anticipated effect size. We anticipated that the metastatic burden in the treatment group would differ from that in the control group by more than 50%, corresponding to a large standardized effect size (Cohen's d ≥ 2.0). Power analysis was conducted using the simplified KISS method (Festing, 2018) and G*Power software. Based on these analyses, a sample size of *n* = 5 per group was determined to provide sufficient power (α = 0.05, 1‐*β* = 0.80) to detect such an effect. The study design adheres to the 3Rs principles (Replacement, Reduction, Refinement) to minimize animal use while ensuring scientific rigor.

### In Vivo Metastasis Assays

Nude male BALB/c mice were used as previously described. Luciferase‐expressing A549 cells (2×10⁶ in 100 µL PBS) were injected into the lateral tail vein to assess lung metastasis. For treatment studies, mice received: ZCL278 (20 mg kg^−1^, i.p.)^[^ ^22^ ^]^ or DMSO every other day for 28 days; (NH_4_)_2_MoS_4_ (80 mg kg^−1^, oral gavage)^[^ ^34^ ^]^ daily; Oligomycin A (0.5 mg kg^−1^, i.v.) every other day for 14 days. No overt toxicity or body weight loss was observed in mice at the administered inhibitor doses, with all animals maintaining a favorable overall health status throughout the study.

Bioluminescence was induced by intraperitoneal D‐luciferin (150 mg kg^−1^) injection at the indicated times and captured using the IVIS Lumina III Spectrum System (PerkinElmer) after 10 min. At the study endpoint, lungs were harvested, metastases quantified, and paraffin‐embedded sections analyzed via H&E staining.

### In Vivo Extravasation Analysis

Mice were anesthetized at 4 and 24 h after GFP⁺A549 cell injection via the tail vein. The thoracic and abdominal cavities were opened to expose the heart and lungs, followed by pulmonary perfusion until the lungs turned white. Lungs were then fixed with 4% paraformaldehyde (PFA), cryosectioned, and subjected to immunofluorescence staining. Fluorescence microscopy was used to assess metastatic cell localization and abundance.

### In Vitro Bioluminescence Imaging (BLI)

Firefly luciferase‐expressing cells (1 × 10⁶ per mouse) were intravenously injected into nude mice. After 4 days, mice were anesthetized, and lung tissues were collected after post‐pulmonary perfusion. Excised lungs were incubated in PBS with 15 mg mL^−1^ D‐luciferin for 15 min, washed, and imaged on a black background using the PerkinElmer IVIS Spectrum system. Bioluminescence was analyzed with Living Image software.

### Intraperitoneal Injection Experiment

Nude mice were injected intraperitoneally with luciferase‐expressing A549 cells (5 × 10⁶/100 µL PBS) using a sterile syringe. Mice were monitored throughout the experiment and euthanized at the endpoint. Intraperitoneal adhesion and organ morphology (heart, liver, spleen, lungs, kidneys) were evaluated.

### LUAD Patient‐Derived Organoid Culture

LUAD tissues for organoid culture were obtained from post‐surgery LUAD patients at Shandong Provincial Hospital. The fresh tissue was rinsed with DPBS, cut into 1–3 mm^3^ fragments, and digested at 37 °C for 30 min with tumor tissue digestive solution (Biogenous, China). After digestion, 2% fetal bovine serum (FBS) was added, and the suspension was filtered through a 100 µm strainer. Cells were collected by centrifugation at 250×g for 3 min, and the supernatant was discarded. The pellet was resuspended in tumor organoid basal medium, centrifuged again, and repeated once more. The final cell pellet was mixed with Basement Membrane Extract (BME, Corning), plated into 24‐well plates, and cultured as 3D organoids. After solidification, 500 µL of organoid complete medium (Biogenous, China) was added to each well, with the medium being replaced every other day. The cultures were maintained in a humidified incubator at 37 °C with 5% CO_2_ for 7–10 days.

For stable transfection, the 3D cultured organoids were dissociated and plated in 2D culture. After adherence, cells were transfected with lentiviruses and polybrene (5 µg mL^−1^), and selected with puromycin (1.5 µg mL^−1^) for 5 days. Surviving organoids were digested again and re‐embedded in Matrigel for passaging.

### Western Blotting Analysis

Cells were lysed in RIPA buffer (Solarbio) with protease and phosphatase inhibitors. Lysates were separated on 10%–13% SDS‐PAGE gels and transferred to 0.22 or 0.45 µm PVDF membranes. Membranes were blocked with 5% BSA in TBST for 2 h and incubated overnight with primary antibodies at 4 °C. After washing with TBST, membranes were probed with HRP‐conjugated secondary antibodies (Invitrogen) for 1 h. Protein bands were detected by chemiluminescence (Millipore) on a Tanon 5200 system and analyzed using ImageJ (v1.52).

Primary antibodies: ACTIN (1:5000, Proteintech, 81115‐1‐RR); Coa4 (1:2500, Invitrogen, PA5‐59076); CDC42 (1:1000, Proteintech, 10155‐1‐AP); E2F1 (1:1000, Proteintech, 12171‐1‐AP); MTCO1 (1:500, ABclonal, A17889); MTCO2 (1:3000, Proteintech, 55070‐1‐AP); MTCO3 (1:2000, Proteintech, 55082‐1‐AP); KRAS (1:10000, Proteintech, 12063‐1‐AP); FAK (1:5000, ABclonal, A11131); p‐FAK‐Y397 (1:300, ABclonal, AP0302).

### Quantitative Real‑Time PCR

Total RNA was extracted using TRIzol (Invitrogen, USA), and cDNA was synthesized with the PrimeScript RT reagent kit (TAKARA, Japan) per the manufacturer's instructions. RT‐qPCR was conducted using the SYBR Green Premix Pro Taq HS qPCR Kit (Agbio, AG11701) on a Roche.LightCycler 480II. Primer sequences are listed in **Table** **3**.

**Table 3:** Primer sequences for quantitative real‑time PCR analysis of target genes.

Genes	Sequence
ACTINFW	TGGCACCCAGCACAATGAA
ACTINRV	CTAAGTCATAGTCCGCCTAGAAGCA
COA4FW	TGGTACTAGTGGCATTACATGGA
COA4RV	CAGGTCCTACAGTGTCTCA
KRASFW	GAGTACAGTGCAATGAGGGAC
KRASRV	CCTGAGCCTGTTTTGTGTCTAC
E2F1FW	ACTGACCATCAGTACCTGGC
E2F1RV	CGGGGATTTCACACCTTTTCC
MTCO1FW	CAAACGCCCCTTTTCGTCT
MTCO1RV	GTGTTGAGGTTGCGGTCTGTT
MTCO2FW	CATGAGCTGTCCCCACATTAG
MTCO2RV	CGGTCGTGTAGCGGTGAAA
MTCO3FW	TCACCCCGCTAAATCCCCTA
MTCO3RV	CGTCGGAAATGGTGAAGGGA
COX5AFW	GGGTAACATACTTCAACAAGCC
COX5ARV	AGTTGGTCTAAGTTCCTGGATG
COX5BFW	ACTGGACCCATACAATGTACTG
COX5BRV	ACAGATGCAGCCTACTATTCTC
COX6AFW	CATGCTGAATGTGTACCTGAAG
COX6ARV	GTACACTTAGGTGAGGATTGAC
COX6BFW	ACCAGAACCAGACTAGAAACTG
COX6BRV	CACAGAGATATCGCCTCCTTTA
COX7AFW	ACAGAAGCTCTTCCAGGAGGACA
COX7ARV	CAGCGTCATTGTCACTCGGT
COX7BFW	GCACTAAATCGTCTCCAAGTTC
COX7BRV	CCGACTTGTGTTGCTACATATG
COX7CFW	GAGCATCCGGAGGTTCACAA
COX7CRV	CACTGAAAATGGCAAATTCTTCCC
COX8AFW	GAAGCTTGGGATCATGGAATTG
COX8ARV	TAGGTCTCCAGGTGTGACAG
CDC42FW	GCTTGTTGGGACTCAAATTGAT
CDC42RV	CCTTTCTGTGTAAGTGCAGAAC
TCF4FW	GACTCGCCAGGCTATCCTTC
TCF4RV	AAGGGTCACTGCTGTGATGG
ZNF460FW	GTCCTCCTTTGGGTGGCTAT
ZNF460RV	CGAAGGTCACAGACTCCCTG

### Mitochondrial DNA Copy Number

Total DNA was extracted using the Fast Pure Cell/Tissue DNA Isolation Mini Kit (Vazyme, DC102‐01) per the manufacturer's protocol. Mitochondrial DNA (mtDNA) copy number was quantified using 1 µg of total DNA and primers targeting the mitochondrial D‐loop region. The nuclear gene G6PC served as an internal reference, and the mtDNA‐to‐nuclear DNA ratio was calculated. Primer sequences: D‐Loop 2: F‐GGCTCTCAACTCCAGCATGT; R‐AGGACGAGGGAGGCTACAAT;G6PC: F‐CTGTCTTTGATTCCTGCCTCAT; RGTGGCTGTGCAGACATTCAA.

### Immunohistochemistry (IHC) Analysis

Tissue sections were baked at 65 °C for 3 h, deparaffinized with xylene, and rehydrated through graded ethanol. Antigen retrieval was performed in sodium citrate buffer (pH 6.0), followed by 3% hydrogen peroxide treatment to quench endogenous peroxidase. Sections were blocked with 5% goat serum and incubated overnight at 4 °C with primary antibodies. After washing, they were incubated with HRP‐conjugated goat anti‐mouse/rabbit IgG for 1 h at room temperature. DAB staining was performed using the DAB kit (Zsbio, ZLI‐9018) per the manufacturer's instructions.

### Immunofluorescence (IF) Assay

For mitochondrial staining, cells were incubated with 100 nm MitoTracker Red CMXRos at 37 °C for 30 min. After PBS washing, cells were fixed in 4% PFA, permeabilized with 0.3% Triton X‐100, and blocked with 1% goat serum for 1 h. Primary antibodies were incubated overnight at 4 °C, followed by secondary antibody incubation at 37 °C for 1 h. Nuclei were counterstained with DAPI.

For lung adenocarcinoma organoids, Matrigel‐cultured organoids were washed with PBS, dissociated, fixed in 4% PFA, embedded in agarose, further fixed, and sectioned. Fluorescence images were captured using a confocal microscope (Olympus Microsystems, Japan), and analysis was performed with ZEN 2.0 software. Primary antibodies: Coa4 (1:350, Invitrogen, PA5‐59076), CDC42 (1:75, Santa Cruz, sc‐8401), MTCO1 (1:250, ABclonal, A17889).

### Copper Sensor‐1 Labeling

Cells (1 × 10⁴ cells mL^−1^) were cultured in confocal dishes and incubated with 5 µL of Copper Sensor‐1 (CS1; HY‐141511, MedChem Express, Shanghai, China) at a final concentration of 5 µm in 1 mL PBS. CS1 stock solution (1 mm) was prepared in dimethyl sulfoxide (DMSO). After incubation for 15 min in the dark at 37 °C, cells were imaged using a confocal microscope with excitation at 543 nm.

### F‐Actin Staining Assay

For staining, dilute 1–5 µL of the phalloidin stock solution in 200 µL PBS, then add to the coverslip or well, ensuring complete coverage. Incubate the cells for 20 min at room temperature in a humidified chamber to prevent evaporation. Apply an antifade mounting medium before imaging by fluorescence microscopy.

### Immunoprecipitation (IP)

IP was performed using Protein A/G Magnetic Beads (MCE, HY‐K0202) as per the manufacturer's instructions. Cells transfected with plasmids were lysed on ice in Pierce IP Lysis Buffer for 30 min. After centrifugation, the supernatant was incubated overnight at 4 °C with the primary antibody and beads with gentle rotation. The immunocomplex was washed, boiled in SDS‐PAGE loading buffer, and analyzed by Western blot.

### Co‑Immunoprecipitation (Co‑IP) Assay

For Co‐IP, 293T cells transfected with Flag‐COA4 and HA‐CDC42 plasmids were lysed in Pierce IP Lysis Buffer (Thermo, 87788) containing protease and phosphatase inhibitors. Subsequent steps were performed following the standard IP protocol.

### Mass Spectrometry‐Based Proteomics

A549 cells transfected with Flag‐COA4 for 48 h were lysed in Pierce IP Lysis Buffer with protease and phosphatase inhibitors. The lysates underwent immunoprecipitation using anti‐FLAG antibody and Pierce Co‐IP Kit (Thermo, 14321D). Eluates were separated by 12% SDS‐PAGE and stained with Coomassie blue (TIANGEN, PA101‐01). Distinct protein bands were excised and analyzed by LC‐MS/MS using the Q Exactive Plus mass spectrometer (Thermo). Following *COA4* knockdown in A549 cells, total protein was collected and subjected to LC‐MS/MS analysis using a Q Exactive Plus mass spectrometer (Thermo Fisher Scientific).

### RNA‐Seq

RNA sequencing (RNA‐Seq) was performed on *COA4* knockout (shCOA4), overexpression (COA4), and vector cells by Beijing Boyun Huakang Gene Technology Co., Ltd. Enrichment analysis was conducted using Gene Ontology (GO) and Kyoto Encyclopedia of Genes and Genomes (KEGG) databases.

### Wound Healing Assay

A sterile pipette tip created a linear scratch on a confluent monolayer of lung adenocarcinoma cells. After PBS wash, serum‐free medium was added to assess migration. Images of the scratch area were taken at 0, 24, and 48 h. Migration distance or wound closure percentage was quantified to evaluate healing.

### Cell Proliferation Assay (CCK‐8, Colony Formation Assay, and EdU)

Cell proliferation was measured using the Cell Counting Kit‐8(BIOSS, BA00208) as per the manufacturer's instructions. A colony formation assay was performed by plating 1000 cells per well in a 6‐well plate to assess long‐term proliferation. Additionally, cell proliferation was conducted using the Cell‐Light EdU Apollo567 In Vitro Kit (RiboBio, C10310‐1) per the manufacturer's instructions.

### Transwell Assay

Migration and invasion of lung adenocarcinoma cells were assessed using a Transwell chamber (BD, 3422). Matrigel (BD Biosciences) was applied to the inserts for invasion.

Cells were seeded in the upper chamber, cultured at 37 °C 5% CO_2_, then fixed, stained with crystal violet (Beyotime), and counted under a microscope after incubation.

### Trans‑Endothelium Migration Assay

HUVEC cells were seeded in a Transwell chamber and grown for 24 h to confluence. Stably transfected GFP^+^ LUAD cells (4 × 10⁵) were added to the apical chamber. The basolateral chamber was filled with 650 µL of culture medium containing 20% fetal bovine serum. Only migrated GFP+ LUAD cells were detected.

### Soft Agar Colony Formation Assay

A 1.2% agarose solution mixed with 20% RPMI‐1640 medium (1:1) was added to each well of a 6‐well plate and allowed to solidify. A 0.7% agarose solution with 20% RPMI‐1640 medium (1:1) was prepared, and 5 × 10^3^ cells were resuspended and added to the solidified bottom layer. The plate was incubated for 3 weeks, with 200 µL medium added every 3 days. After 21 days, colonies were counted from random sections of each plate.

### In situ Proximity Ligation Assay (PLA)

Protein–protein interactions were analyzed using the PLA reagent (Sigma–Aldrich, DUO92101) according to the manufacturer's instructions. PLA signals were visualized using the IXplore SpinSR10 microscope (Olympus).

### Structure Illumination Microscopy (SIM)

SIM imaging was performed on a Zeiss ELYRA 7 with Lattice‐SIM microscope (Carl Zeiss Microimaging). A549 cells were washed twice with PBS, incubated with 100 nM MitoTracker Red CMXRos for 30 min, and fixed with 4% PFA for 15 min. Nuclei were counterstained with DAPI. Images were processed and reconstructed using the SIM module in Zen Black software (Carl Zeiss Microimaging).

### Seahorse (OCR and ECAR)

The OCR and extracellular acidification rate (ECAR) of LUAD cells were measured using the Seahorse XFe96 Extracellular Flux Analyzer (Agilent Technologies, Santa Clara, CA, USA). Cells were seeded at a density of 8 × 10^3^ cells per well in XF96 microplates and incubated overnight at 37 °C under 5% CO_2_. Measurements were performed using the Seahorse XF Cell Mito Stress Test Kit and the Seahorse XF Glycolysis Stress Test Kit according to the manufacturer's instructions. On the assay day, media in the wells were replaced with Seahorse XF RPMI medium (pH 7.4, 5 mm HEPES), containing the same concentrations of each PPAR agonist. The medium was supplemented with 1 mm pyruvate, 2 mm glutamine, and 10 mm glucose for the Mito Stress Test. The Glycolysis Stress Test included only 2 mm glutamine. In the Mito Stress Test, cells were treated with 1.5 µm Oligomycin, followed by 1 µm FCCP after 15 min, and then 0.5 µm Rotenone and Antimycin A for 15 min. The Glycolysis Stress Test involved treating cells with 10 mm glucose, 1.0 µm Oligomycin after 15 min, and 50 mm 2‐deoxy‐D‐glucose.

### Cytochrome C Oxidase Activity Assay

COX activation was assessed using the Mitochondrial COX/Cytochrome C Oxidase Activity Kit (Solarbio, BC0940), according to the manufacturer's instructions.

### ATP Content Determination

ATP content was measured using the Enhanced ATP Assay Kit (Beyotime, S0027) according to the manufacturer's instructions.

### Luciferase Activity

The pGL3‐Basic firefly luciferase plasmid, pRL‐TK Renilla luciferase plasmid, and E2F1 pcDNA3.1 plasmid were co‐transfected into A549 and 293T cells using Lipofectamine 3000. After 48 h, luciferase activity was measured using the Dual‐Luciferase Reporter Assay System (Promega, E1910) on a Spark Multi‐Functional Microplate Reader (TECAN SPARK).

### Chromatin Immunoprecipitation (ChIP) Assay

ChIP assays were performed using the ChIP assay kit (Merck) per the manufacturer's instructions. A549 cells were crosslinked with 1% formaldehyde (Sigma) for 10 min and quenched with 125 mm glycine. Chromatin was fragmented by sonication and immunoprecipitated with antibodies against E2F1 and Protein A/G magnetic beads at 4 °C overnight. The beads were separated, and the immunoprecipitated DNA was eluted. DNA enrichment was quantified by RT‐qPCR with specific primers: F‐ATTCTCCTGCCTCAGCCTCCC; R‐TCGAGACCAGCCTGACCAACAT'). Rabbit IgG served as a negative control.

### CDC42 GTP Pull‐Down Assay

CDC42 activation was assessed using a CDC42 Activation Assay Kit (Cytoskeleton, CSK‐BK034‐S) following the manufacturer's instructions.

### Transmission Electron Microscopy

A549 cells were fixed and embedded in epoxy resin. Semithin sections were stained with methylene blue, and ultrathin sections were prepared using a diamond knife, stained with uranyl acetate and lead citrate, then examined under a transmission electron microscope.

### Single‐Cell Time‐Lapse Imaging

Cells were plated in a 24‐well plate, treated with drugs, and imaged every 2 h for 24 h using a high‐content imaging system. Migration was analyzed with the TrackMate plugin in ImageJ, and statistical analysis was performed using GraphPad Prism 8.

### Detection of Cell Membrane Potential by JC‐10

A549 cells were seeded in confocal dishes and treated with drugs. The JC‐10 probe was prepared as per the manufacturer's protocol, and fluorescence imaging was performed. Changes in mitochondrial potential were assessed by fluorescence shifts.

### Construction of Cox Proportional Hazards Regression Model

A cohort of 779 patients with LUAD from The Cancer Genome Atlas (TCGA‐LUAD) database was analyzed, comprising cases with complete clinicopathological characteristics and survival data. Overall survival (OS) was assessed using a Cox proportional hazards regression model. COA4 expression levels were stratified into low, intermediate, and high groups based on expression tertiles. The model was adjusted for age, sex, and pathological stage. All statistical analyses were performed in the R software environment using the coxph() function from the survival package.

### Statistical Analysis

All analyses were performed using GraphPad Prism 8.0.2 (GraphPad Software, San Diego, CA). Data are presented as mean ± SD from ≥3 independent experiments and displayed as bar graphs. Normality and homogeneity of variances were assessed by Shapiro–Wilk and F tests, respectively. For comparisons: Between two groups with equal variance: Two‐tailed unpaired Student *t*‐test; Among multiple groups with normal distribution: One‐way/ two‐way ANOVA followed by Dunnett's or Tukey's post hoc test; Categorical variables were analyzed using the Chi‐square test or Fisher's exact test, as appropriate. Survival curves were generated by Kaplan–Meier analysis, and group differences were evaluated by the log‐rank (Mantel–Cox) test. Statistical significance was defined as *p* < 0.05 (two‐tailed). Exact sample size (*n*), statistical tests, and *p*‐values are specified in figure legends.

## Conflict of Interest

The authors declare no conflict of interest.

## Author Contributions

X.J., W.Z., F.X., J. Z., and Q.Z. contributed equally to this work as co‐first authors. L.Y., J.X.Z., and W.Q. conceived the study, designed the experiments, and wrote the manuscript. M.G.Y. collected specimens. J.X.Z., Z.W.Y., X.F.Y., Z.J.Z., Z.Q.H., S.X.M., S.J., Z.H., X.Q.L., M.G.Y., S.S.N., W.Y., M.Q., analyzed the data; J.X.Z., Z.W.Y., X.F.Y., Z.J.Z., Z.Q.H., S.J., S.S.N., W.Y., M.Q., performed the experiments. J.X.Z., Z.W.Y., X.F.Y., Z.J.Z., Z.Q.H., L.Y., and W.Q. write, edit, and proof the manuscript. All authors approved the final submitted version of the manuscript.

## Declarations—Ethics Approval and Consent to Participate

The study was conducted following the Declaration of Helsinki. Written informed consent for research was obtained from each patient. The collection of human specimens was approved by the Ethics Committee of Shandong Provincial Hospital Affiliated to Shandong First Medical University, Jinan, China (NSFC: NO.2023‐225). Animal studies were performed according to the study protocol approved by the Animal Ethics Committee of Shandong Provincial Hospital Affiliated to Shandong First Medical University (NO. NSFC2024‐551) and were performed according to the international guidelines and the Basel Declaration.

## Consent for Publication

The manuscript has been approved by all authors for publication.

## Supporting information



Supporting Information

## Data Availability

The data that support the findings of this study are available from the corresponding author upon reasonable request.

## References

[1] H. Sung , J. Ferlay , R. L. Siegel , M. Laversanne , I. Soerjomataram , A. Jemal , F. Bray , CA Cancer J. Clin. 2021, 71, 209. 33538338 10.3322/caac.21660

[2] a) L. Ma , X. Xue , X. Zhang , K. Yu , X. Xu , X. Tian , Y. Miao , F. Meng , X. Liu , S. Guo , S. Qiu , Y. Wang , J. Cui , W. Guo , Y. Li , J. Xia , Y. Yu , J. Wang , J. Exp. Clin. Cancer Res 2022, 41, 36; 35078505 10.1186/s13046-021-02200-5 PMC8788079

[3] X. Yang , Q. Bai , W. Chen , J. Liang , F. Wang , W. Gu , L. Liu , Q. Li , Z. Chen , A. Zhou , J. Long , H. Tian , J. Wu , X. Ding , N. Zhou , M. Li , Y. Yang , J. Cai , Adv. Sci. (Weinh) 2023, 10, 2206744. 37171793 10.1002/advs.202206744 PMC10369244

[4] D. Consonni , M. Pierobon , M. H. Gail , M. Rubagotti , M. Rotunno , A. Goldstein , L. Goldin , J. Lubin , S. Wacholder , N. E. Caporaso , P. A. Bertazzi , M. A. Tucker , A. C. Pesatori , M. T. Landi , J. Natl. Cancer Inst. 2015, 107, djv059. 25802059 10.1093/jnci/djv059 PMC4838060

[5] J. Massagué , K. Ganesh , Cancer Discov. 2021, 11, 971. 33811127 10.1158/2159-8290.CD-21-0010 PMC8030695

[6] a) A. L. Oliver , Surg. Clin. North Amer. 2022, 102, 335; 35671760 10.1016/j.suc.2021.12.001

[7] a) M. Yuan , L.‐L. Huang , J.‐H. Chen , J. Wu , Q. Xu , Sign. Transd. Target. Ther. 2019, 4, 61; 10.1038/s41392-019-0099-9 PMC6914774 31871778

[8] D. K. Simanshu , D. V. Nissley , F. McCormick , Cell 2017, 170, 17. 28666118 10.1016/j.cell.2017.06.009 PMC5555610

[9] a) J. Canon , K. Rex , A. Y. Saiki , C. Mohr , K. Cooke , D. Bagal , K. Gaida , T. Holt , C. G. Knutson , N. Koppada , B. A. Lanman , J. Werner , A. S. Rapaport , T. San Miguel , R. Ortiz , T. Osgood , J. R. Sun , X. Zhu , J. D. McCarter , L. P. Volak , B. E. Houk , M. G. Fakih , B. H. O'Neil , T. J. Price , G. S. Falchook , J. Desai , J. Kuo , R. Govindan , D. S. Hong , W. Ouyang , et al., Nature 2019, 575, 217; 31666701 10.1038/s41586-019-1694-1

[10] J. Son , C. A. Lyssiotis , H. Ying , X. Wang , S. Hua , M. Ligorio , R. M. Perera , C. R. Ferrone , E. Mullarky , N. Shyh‐Chang , Y. Kang , J. B. Fleming , N. Bardeesy , J. M. Asara , M. C. Haigis , R. A. DePinho , L. C. Cantley , A. C. Kimmelman , Nature 2013, 496, 101. 23535601 10.1038/nature12040 PMC3656466

[11] a) Y. Shi , H. Zheng , T. Wang , S. Zhou , S. Zhao , M. Li , B. Cao , Mol. Cancer 2025, 24, 9; 39799325 10.1186/s12943-024-02216-3 PMC11724471

[12] D. Pore , N. Mahata , A. Pal , M. K. Chakrabarti , Mol. Immunol. 2010, 47, 1739. 20347487 10.1016/j.molimm.2010.03.001

[13] a) M. Han , E. A. Bushong , M. Segawa , A. Tiard , A. Wong , M. R. Brady , M. Momcilovic , D. M. Wolf , R. Zhang , A. Petcherski , M. Madany , S. Xu , J. T. Lee , M. V. Poyurovsky , K. Olszewski , T. Holloway , A. Gomez , M. S. John , S. M. Dubinett , C. M. Koehler , O. S. Shirihai , L. Stiles , A. Lisberg , S. Soatto , S. Sadeghi , M. H. Ellisman , D. B. Shackelford , Nature 2023, 615, 712; 36922590 10.1038/s41586-023-05793-3 PMC10033418

[14] I. Martínez‐Reyes , L. R. Cardona , H. Kong , K. Vasan , G. S. McElroy , M. Werner , H. Kihshen , C. R. Reczek , S. E. Weinberg , P. Gao , E. M. Steinert , R. Piseaux , G. R. S. Budinger , N. S. Chandel , Nature 2020, 585, 288. 32641834 10.1038/s41586-020-2475-6 PMC7486261

[15] a) K. Nie , J. Li , X. He , Y. Wang , Q. Zhao , M. Du , H. Sun , J. Wang , J. Lyu , H. Fang , L. Jin , Oncogenesis 2020, 9, 51; 32415061 10.1038/s41389-020-0231-2 PMC7229118

[16] a) A. B. Swaminathan , S. Soma , A. C. Vicary , M. Zulkifli , H. Kaur , V. M. Gohil , Genetics 2022, 221, iyac090; 35666203 10.1093/genetics/iyac090 PMC9339329

[17] a) M. Bestwick , M. Y. Jeong , O. Khalimonchuk , H. Kim , D. R. Winge , Mol. Cell. Biol. 2010, 30, 4480; 20624914 10.1128/MCB.00228-10 PMC2937524

[18] a) Y. Luo , B. Lv , S. He , K. Zou , K. Hu , Int. J. Gen. Med. 2021, 14, 1773; 33994806 10.2147/IJGM.S312277 PMC8113014

[19] S. Telang , K. K. Nelson , D. L. Siow , A. Yalcin , J. M. Thornburg , Y. Imbert‐Fernandez , A. C. Klarer , H. Farghaly , B. F. Clem , J. W. Eaton , J. Chesney , Mol. Cancer 2012, 11, 60. 22917272 10.1186/1476-4598-11-60 PMC3546037

[20] Y. Li , J. S. Park , J. H. Deng , Y. Bai , J. Bioenerg. Biomembr. 2006, 38, 283. 17091399 10.1007/s10863-006-9052-z PMC1885940

[21] D. Murata , S. Roy , S. Lutsenko , M. Iijima , H. Sesaki , Dev. Cell 2024, 59, 2578. 38986607 10.1016/j.devcel.2024.06.008 PMC11461135

[22] X. Zhang , L. Ren , J. Wu , R. Feng , Y. Chen , R. Li , M. Wu , M. Zheng , X. G. Wu , W. Luo , H. He , Y. Huang , M. Tang , J. Li , J. Exp. Clin. Cancer Res. 2022, 41, 230. 35869555 10.1186/s13046-022-02441-y PMC9308268

[23] X. Zhao , J.‐L. Guan , Adv. Drug Delivery Rev. 2011, 63, 610. 10.1016/j.addr.2010.11.001 PMC3132829 21118706

[24] C. Lehmer , M. H. Schludi , L. Ransom , J. Greiling , M. Junghänel , N. Exner , H. Riemenschneider , J. van der Zee , C. Van Broeckhoven , P. Weydt , M. T. Heneka , D. Edbauer , EMBO Mol. Med. 2018, 10, 8558. 10.15252/emmm.201708558 PMC5991575 29789341

[25] A. Tsherniak , F. Vazquez , P. G. Montgomery , B. A. Weir , G. Kryukov , G. S. Cowley , S. Gill , W. F. Harrington , S. Pantel , J. M. Krill‐Burger , R. M. Meyers , L. Ali , A. Goodale , Y. Lee , G. Jiang , J. Hsiao , W. F. J. Gerath , S. Howell , E. Merkel , M. Ghandi , L. A. Garraway , D. E. Root , T. R. Golub , J. S. Boehm , W. C. Hahn , Cell 2017, 170, 564. 28753430 10.1016/j.cell.2017.06.010 PMC5667678

[26] K. Mondal , M. K. Posa , R. P. Shenoy , S. Roychoudhury , Cells 2024, 13, 1221. 39056802 10.3390/cells13141221 PMC11274496

[27] C. Cerutti , S. Lucotti , S. T. Menendez , N. Reymond , R. Garg , I. A. Romero , R. Muschel , A. J. Ridley , Cell Rep. 2024, 43, 113989. 38536816 10.1016/j.celrep.2024.113989

[28] a) M. D. M. Maldonado , S. Dharmawardhane , Cancer Res. 2018, 78, 3101; 29858187 10.1158/0008-5472.CAN-18-0619 PMC6004249

[29] M. Ahn , S. Dhawan , E. M. McCown , P. A. Garcia , S. Bhattacharya , R. Stein , D. C. Thurmond , Diabetologia 2025, 68, 152. 39404845 10.1007/s00125-024-06286-2 PMC11663170

[30] A. Glogowska , T. Thanasupawat , J. Beiko , M. Pitz , S. Hombach‐Klonisch , T. Klonisch , Mol. Oncol. 2022, 16, 368. 33960104 10.1002/1878-0261.12981 PMC8763656

[31] J. M. Laurent , R. K. Garge , A. I. Teufel , C. O. Wilke , A. H. Kachroo , E. M. Marcotte , PLoS Biol. 2020, 18, 3000627. 10.1371/journal.pbio.3000627 PMC7259792 32421706

[32] B. B. Seo , T. Kitajima‐Ihara , E. K. Chan , I. E. Scheffler , A. Matsuno‐Yagi , T. Yagi , Proc. Natl. Acad. Sci. U S A 1998, 95, 9167. 9689052 10.1073/pnas.95.16.9167 PMC21310

[33] J. Melendez , M. Grogg , Y. Zheng , J. Biol. Chem. 2011, 286, 2375. 21115489 10.1074/jbc.R110.200329 PMC3024731

[34] a) D. C. Brady , M. S. Crowe , D. N. Greenberg , C. M. Counter , Cancer Res. 2017, 77, 6240; 28986383 10.1158/0008-5472.CAN-16-1190 PMC5690876

[35] S. M. Davidson , T. Papagiannakopoulos , B. A. Olenchock , J. E. Heyman , M. A. Keibler , A. Luengo , M. R. Bauer , A. K. Jha , J. P. O'Brien , K. A. Pierce , D. Y. Gui , L. B. Sullivan , T. M. Wasylenko , L. Subbaraj , C. R. Chin , G. Stephanopolous , B. T. Mott , T. Jacks , C. B. Clish , M. G. Vander Heiden , Cell Metab. 2016, 23, 517. 26853747 10.1016/j.cmet.2016.01.007 PMC4785096

[36] a) S. J. Heasman , A. J. Ridley , Nat. Rev. Mol. Cell Biol. 2008, 9, 690; 18719708 10.1038/nrm2476

[37] Z. Zong , F. Xie , S. Wang , X. Wu , Z. Zhang , B. Yang , F. Zhou , Cell 2024, 187, 2375. 38653238 10.1016/j.cell.2024.04.002

[38] P. A. Philip , V. Sahai , N. Bahary , A. Mahipal , A. Kasi , C. M. S. Rocha Lima , A. T. Alistar , P. E. Oberstein , T. Golan , J. P. Metges , J. Lacy , C. Fountzilas , C. D. Lopez , M. Ducreux , P. Hammel , M. Salem , D. Bajor , A. B. Benson , S. Luther , T. Pardee , E. Van Cutsem , J. Clin. Oncol. 2024, 42, 3692. 39088774 10.1200/JCO.23.02659

[39] J. I. Medina , A. Cruz‐Collazo , M. Del Mar Maldonado , T. M. Gascot , L. D. Borrero‐Garcia , M. Cooke , M. G. Kazanietz , E. H. O'Farril , C. P. Vlaar , S. Dharmawardhane , Cancer. Res. Commun. 2022, 2, 1711. 36861094 10.1158/2767-9764.CRC-22-0303 PMC9970268

[40] X. Hu , L. Gan , Z. Tang , R. Lin , Z. Liang , F. Li , C. Zhu , X. Han , R. Zheng , J. Shen , J. Yu , N. Luo , W. Peng , J. Tan , X. Li , J. Fan , Q. Wen , X. Wang , J. Li , X. Zheng , Q. Liu , J. Guo , G. P. Shi , H. Mao , W. Chen , S. Yin , Y. Zhou , Adv. Sci. 2024, 11, 2307850. 10.1002/advs.202307850 PMC10987128 38240457

