# Supplementary material for: Novel Evolutionarily Conserved Oncogene COA4 is Driven by KRAS Mutant and Promotes Cancer Metastasis Through Dual Mitochondrial Metabolism‐Dependent and ‐Independent Mechanisms
Source: Adv Sci (Weinh). 2025 Sep 11;12(42):e07533. doi: 10.1002/advs.202507533 (PMC12622526; doi:10.1002/advs.202507533)
Supplement: Supplementary file 1 — Supporting Information [file ADVS-12-e07533-s001.docx]

**Supplementary Figures**

Figure S1


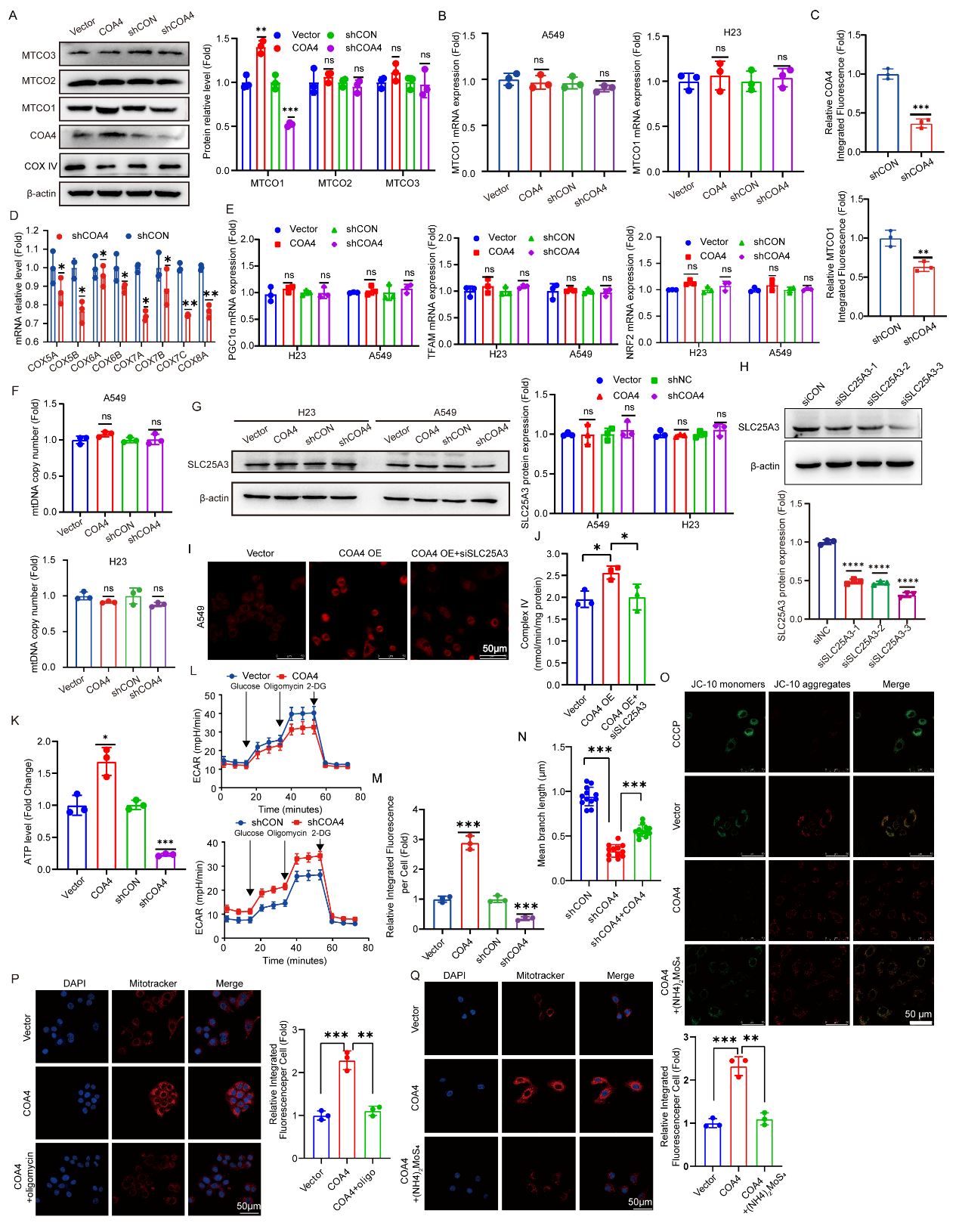


**Figure S1 COA4 manipulates mitochondrial metabolism via regulating cytochrome c oxidase**

A. Western blot analysis of the protein levels of mitochondrial-encoded complex IV subunits in H23 cells with *COA4* knockdown or overexpression. (n = 3; ns, not significant; ***P* < 0.01; ****P* < 0.001)

B. RT-qPCR analysis of *MTCO1* mRNA levels in A549 and H23 cells following *COA4* knockdown or overexpression. (n = 3; ns, not significant)

C. IF intensity of MTCO1 and COA4 in LUAD PDOs following *COA4* knockdown. (n = 3; **P < 0.01; ***P < 0.001).

D. RT-qPCR analysis of mRNA levels for nuclear-encoded complex IV subunits in H23 cells with *COA4* knockdown or overexpression. (n = 3; **P* < 0.05; ***P* < 0.01)

E. RT-qPCR analysis of *PGC1α*, *TFAM*, and *NRF2* mRNA levels in A549 and H23 cells after *COA4* knockdown or overexpression. (n = 3; ns, not significant)

F. Analysis of mitochondrial DNA (mtDNA) copy number in A549 and H23 cells with *COA4* knockdown or overexpression. (n = 3; ns, not significant)

G. Western blot analysis of SLC25A3 protein expression in A549 and H23 cells following *COA4* knockdown or overexpression. (n = 3; ns, not significant)

H. Western blot analysis confirming SLC25A3 knockdown efficiency in A549 cells. (n = 3; ****P < 0.0001)

I. Mitochondrial copper levels visualized by Copper Sensor-1 fluorescence in live A549 cells. Scale bar: 50 μm. (n = 3)

J. COX activity analysis in A549 cells upon *COA4* overexpression following *SLC25A3* knockdown. (n = 3; **P* < 0.05)

K. Measurement of ATP levels in H23 cells with *COA4* knockdown or overexpression. (n=3; **P* < 0.05; ****P* < 0.001)

L. Extracellular acidification rate (ECAR) analysis in A549 cells with *COA4* knockdown and overexpression, measured using the Seahorse XFe96 system.(n = 3)

M. Mitochondrial mass quantification in *COA4*-modulated A549 cells. (n = 3; ****P* < 0.001).

N. Quantitative analysis of mitochondrial fragmentation in *COA4*-modulated A549 cells.

O. Assessment of mitochondrial membrane potential in A549 cells overexpressing *COA4* and treated with (NH_4_)₂MoS₄, analyzed using JC-10 staining. Scale bar: 50 μm. (n = 3)

P-Q. IF analysis of mitochondrial mass using MitoTracker (red) in A549 cells overexpressing *COA4* and treated with oligomycin (P) or (NH_4_)₂MoS₄ (Q), with nuclei counterstained with DAPI. Scale bar: 50 μm. (n = 3; ***P* < 0.01; ****P* < 0.001)

The data are given as mean ± SD and compared by two-tailed unpaired Student’s t-test (A-K). Significance levels are indicated as follows: ns, not significant; **P* < 0.05; ***P* < 0.01; ****P* < 0.001; *****P* < 0.0001.

Figure S2


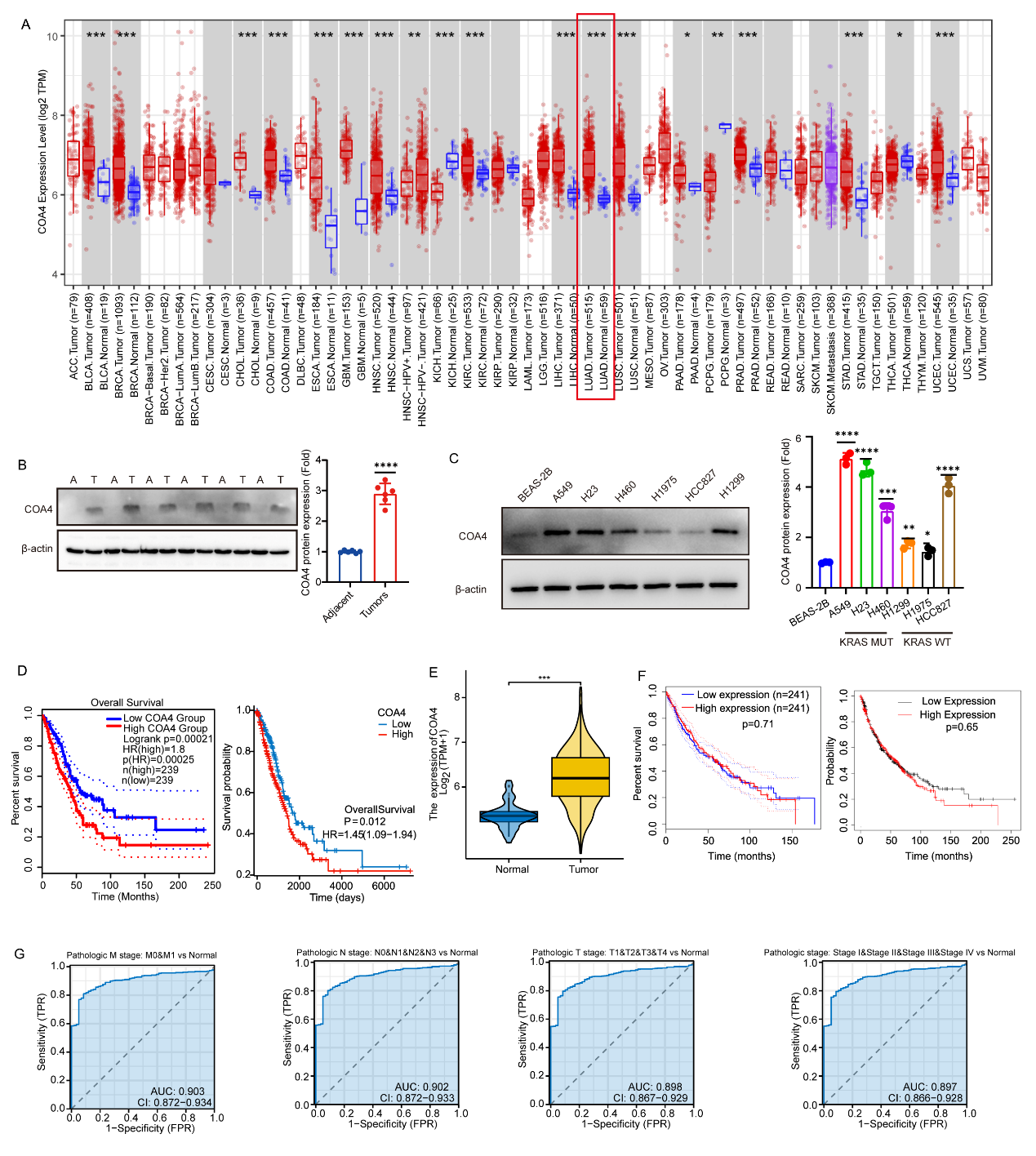


**Figure S2 Elevated *COA4* expression in LUAD correlates with poor prognosis**

A. *COA4* expression across various cancer types analyzed using the Oncomine database.
B. Western blot analysis of COA4 expression levels in LUAD tumor tissues compared with adjacent normal tissues. (n = 6 per group, *****P* < 0.0001)
C. Western blot and RT-qPCR analyses of *COA4* expression in LUAD cell lines. (n = 3, **P* < 0.05; ***P* < 0.01; ****P* < 0.001; *****P* < 0.0001)
D. Prognostic significance of *COA4* in LUAD, evaluated using GEPIA2 (left) and Xiantao Academic (right) databases.
E. *COA4* expression in lung squamous cell carcinoma (LUSC) versus normal tissues, analyzed using the Xiantao Academic database.
F. Survival analysis of *COA4* in LUSC, performed using GEPIA2 (left) and Kaplan-Meier Plotter (right).
G. Diagnostic performance of *COA4* in distinguishing LUAD from normal tissues, as assessed by AUC values for T stage (0.898), N stage (0.902), M stage (0.903), and pathological grades (0.897).

The data are given as mean ± SD and compared by Student’s t test (A-C, E). Survival analyses were plotted using the Kaplan–Meier method, with group differences evaluated with the log-rank (Mantel–Cox) test (D, F). Significance levels are indicated as follows: ns, not significant; **P* < 0.05; ***P* < 0.01; ****P* < 0.001; *****P* < 0.0001.

Figure S3


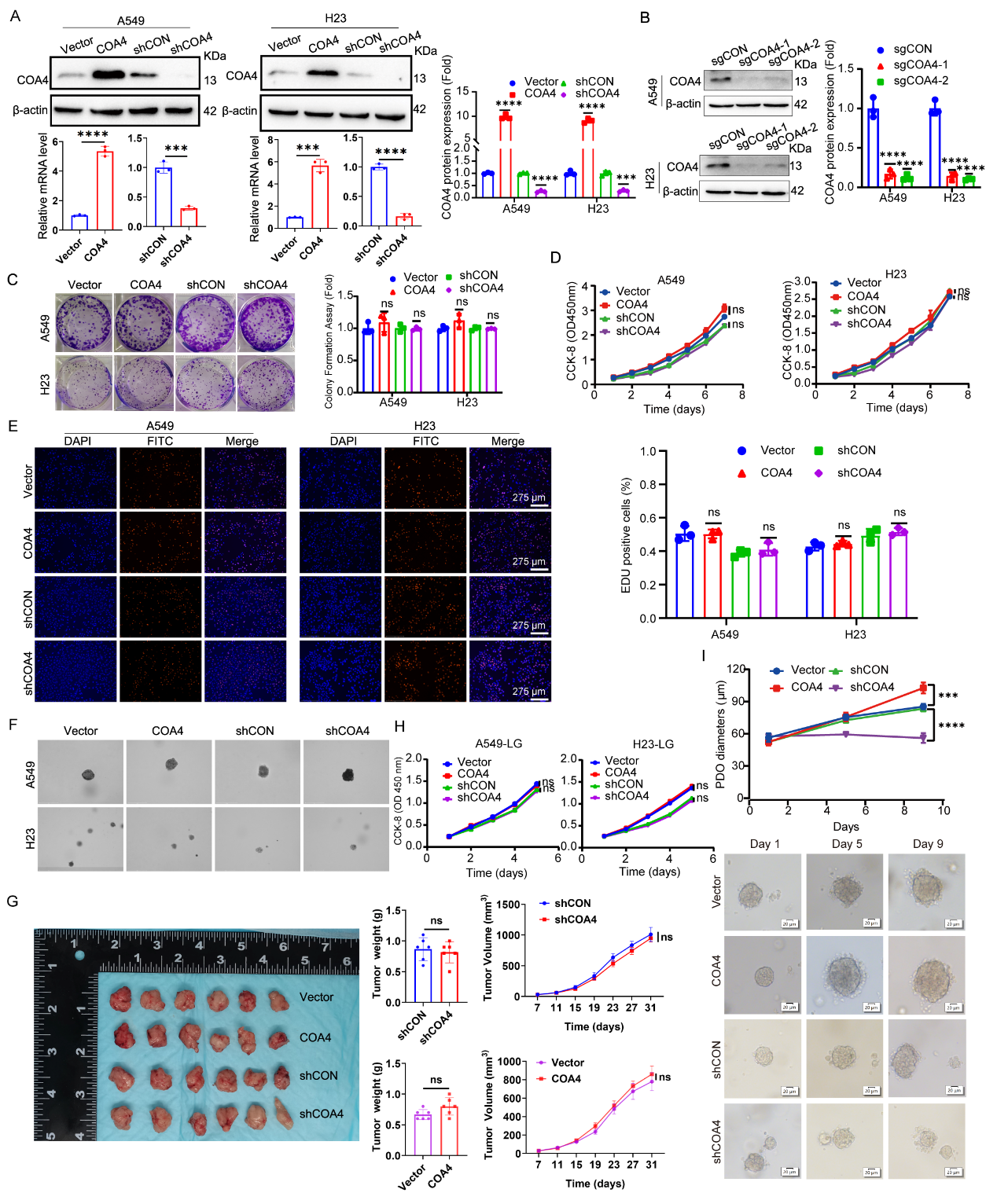


**Figure S3 *COA4* manipulation does not alter proliferation in LUAD**

A. Western blot and RT-qPCR analyses confirming the efficiency of *COA4* knockdown or overexpression. (n = 3, ****P* < 0.001; *****P* < 0.0001)
B. Western blot analysis verifying COA4 knockout efficiency using the CRISPR-Cas9 system. (n = 3, *****P* < 0.0001)
C-F. Quantification of the proliferative ability of A549 and H23 cells with *COA4* knockdown or overexpression by plate colony formation assays (C), CCK-8 assays (D), EdU incorporation assays (E), and soft agar assays (F), with statistical analysis. Scale bar: 275 μm (n = 3, ns, not significant)
G. Measurements of tumor volume and weight in xenografts derived from A549 cells with *COA4* knockdown or overexpression (n = 6 per group, ns, not significant).
H. Proliferation of A549 and H23 cells under hypoglycemic conditions, as quantified by CCK-8 assays following *COA4* knockdown or overexpression.(n = 3, ns, not significant)
I. Imaging of LUAD PDOs subjected to *COA4* overexpression or knockdown, with quantification of organoid diameters. Organoids were maintained in a humidified incubator at 37°C under normoxic conditions for 7-10 days.

The data are given as mean ± SD and compared by Student’s t test (A-C, E-G) and two-way ANOVA (D, G-I). Significance levels are indicated as follows: ns, not significant; **P* < 0.05; ***P* < 0.01; ****P* < 0.001; *****P* < 0.0001.

Figure S4


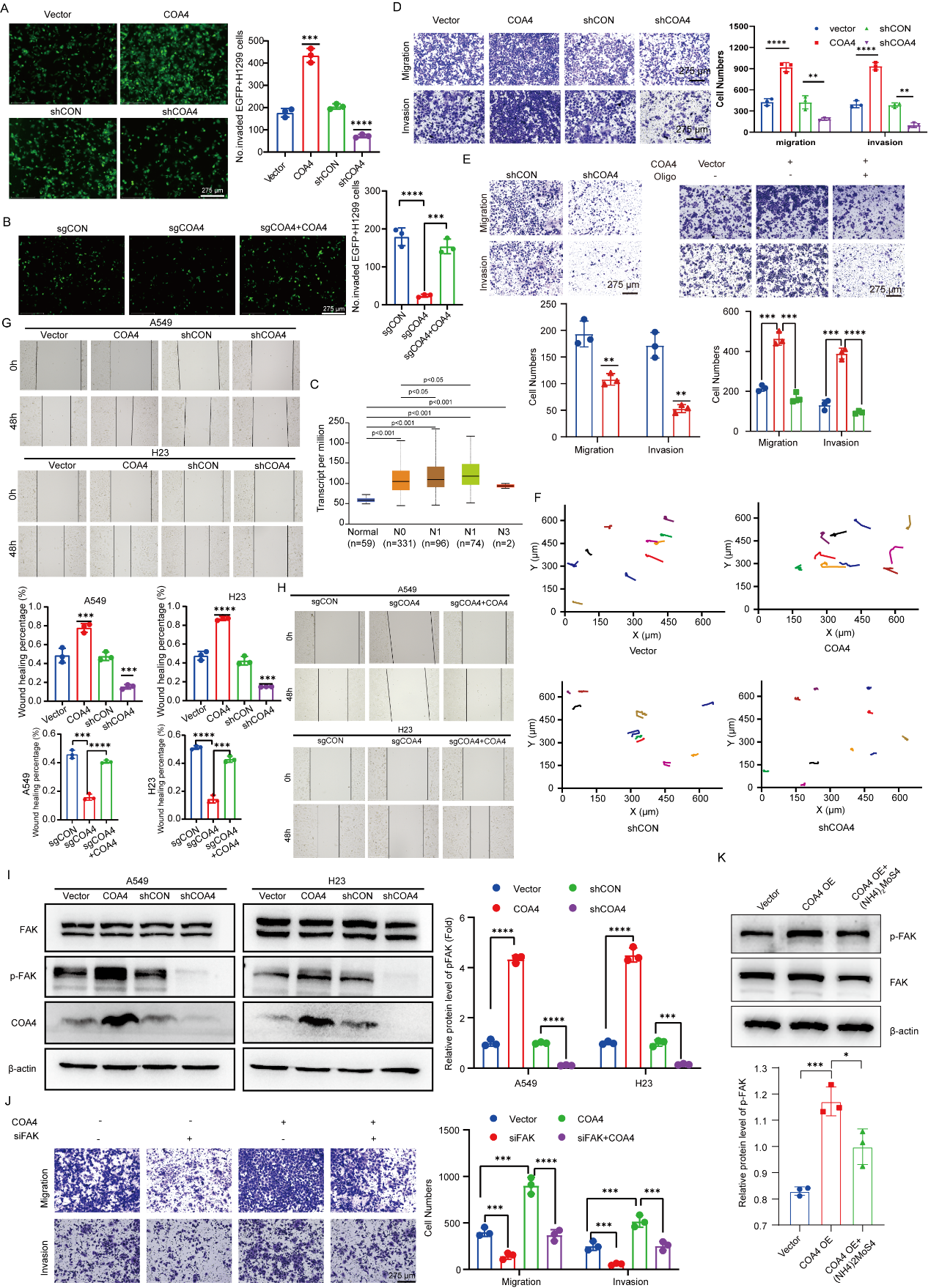


**Figure S4 COA4 involves in invasion and migration of LUAD cells**

A. Trans-endothelial migration assays analyzing the ability of H1299 cells to traverse HUVEC monolayers following *COA4* overexpression or knockdown. Scale bar: 275 μm (n = 3, ****P* < 0.001; *****P* < 0.0001)
B. Trans-endothelial migration assays assessing H1299 cells’ capacity to cross HUVEC monolayers after *COA4* knockout and subsequent rescue by *COA4* re-expression. Scale bar: 275 μm (n = 3, ****P* < 0.001; *****P* < 0.0001)
C. Correlation analysis between *COA4* expression and lymph node metastasis using the UALCAN database.
D. Transwell assays evaluating migration and invasion in H1299 cells following *COA4* knockdown or overexpression. Scale bar: 275 μm (n = 3, ****P* < 0.001; *****P* < 0.0001)
E. Transwell assays quantifying migration and invasion in H23 cells: (left) in *COA4*-knockdown cells, and (right) in *COA4*-overexpressing cells treated with oligomycin. Scale bar: 275 μm (n = 3, ***P* < 0.01; ****P* < 0.001; *****P* < 0.0001)
F. Single-cell time-lapse imaging assays performed on A549 cells with *COA4* overexpression or knockdown, with corresponding movement trajectory plots. (n = 3)
G. Wound healing assays in A549 and H23 cells following *COA4* knockdown or overexpression. Scale bar: 275 μm (n = 3, ****P* < 0.001; *****P* < 0.0001)
H. Wound healing assays in *COA4*-knockout A549 and H23 cells with subsequent *COA4* re-expression. Scale bar: 275 μm (n = 3, ****P* < 0.001; *****P* < 0.0001)
I. Western blot analysis of total FAK and phosphorylated FAK (p-FAK) protein levels in A549 and H23 cells following *COA4* knockdown or overexpression. (n = 3, ****P* < 0.001; *****P* < 0.0001)
J. Transwell assays examining the impact of *FAK* knockdown on the migration and invasion of A549 cells overexpressing *COA4*. Scale bar: 275 μm (n = 3, ****P* < 0.001; *****P* < 0.0001)
K. Western blot analysis of FAK and p-FAK levels in *COA4*-overexpressing H1299 cells treated with (NH_4_)₂MoS₄. (n = 3, **P* < 0.05; ****P* < 0.001)
The data are given as mean ± SD and compared by two-tailed unpaired Student’s t-test (A-E, G-K). Significance levels are indicated as follows: ns, not significant; *P < 0.05; **P < 0.01; ***P < 0.001; ****P < 0.0001.

Figure S5


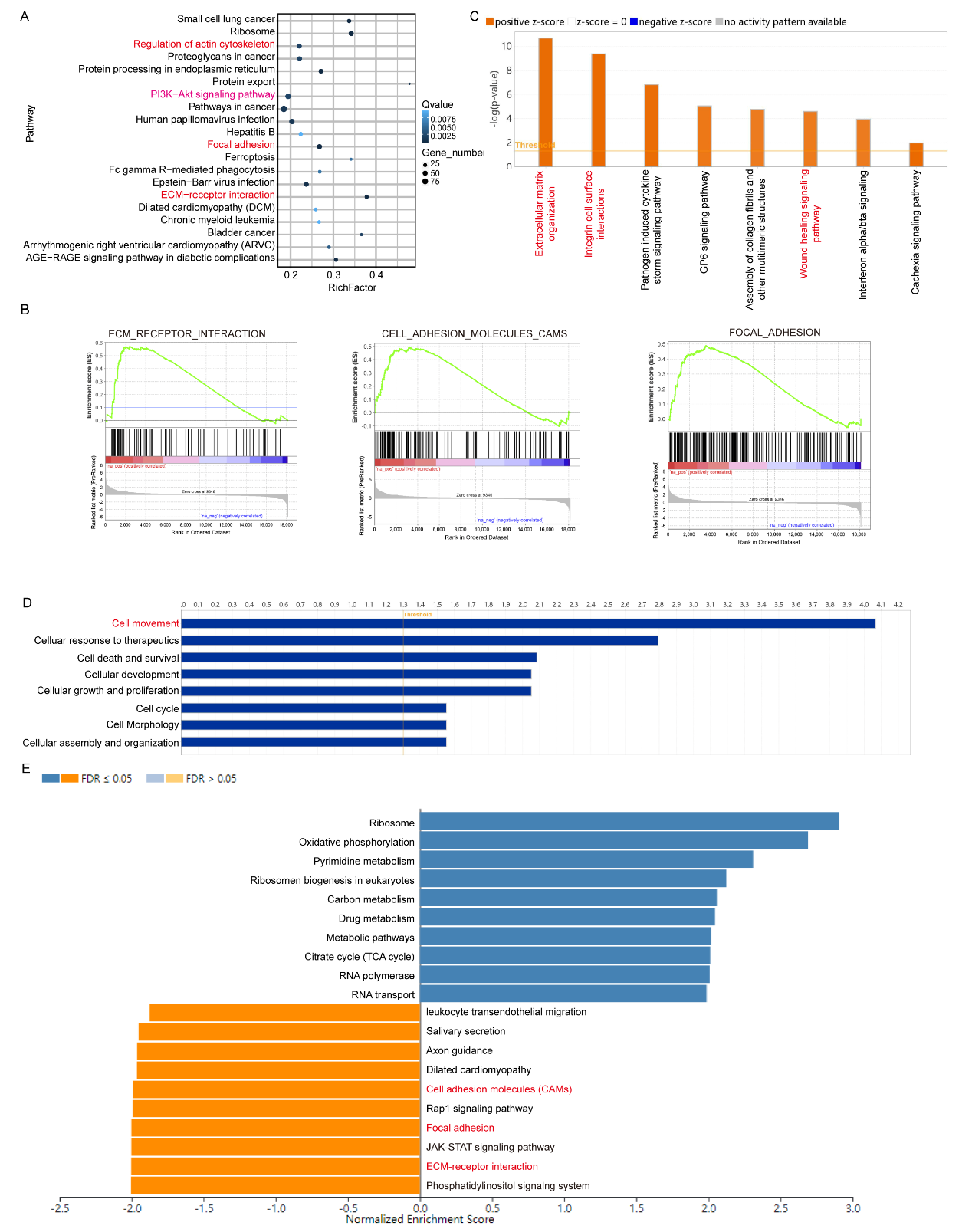


**Figure S5 COA4 modulates invasion-migration signaling pathways in lung adenocarcinoma**

A-E. Transcriptomic sequencing of *COA4*-knockdown A549 cells followed by pathway enrichment analyses: KEGG analysis (A), GSEA (B), IPA (C-D), and co-expression analysis (E), highlighting enrichment in cell motility-related pathways including actin cytoskeleton regulation, focal adhesion, and ECM-receptor interaction.

Figure S6


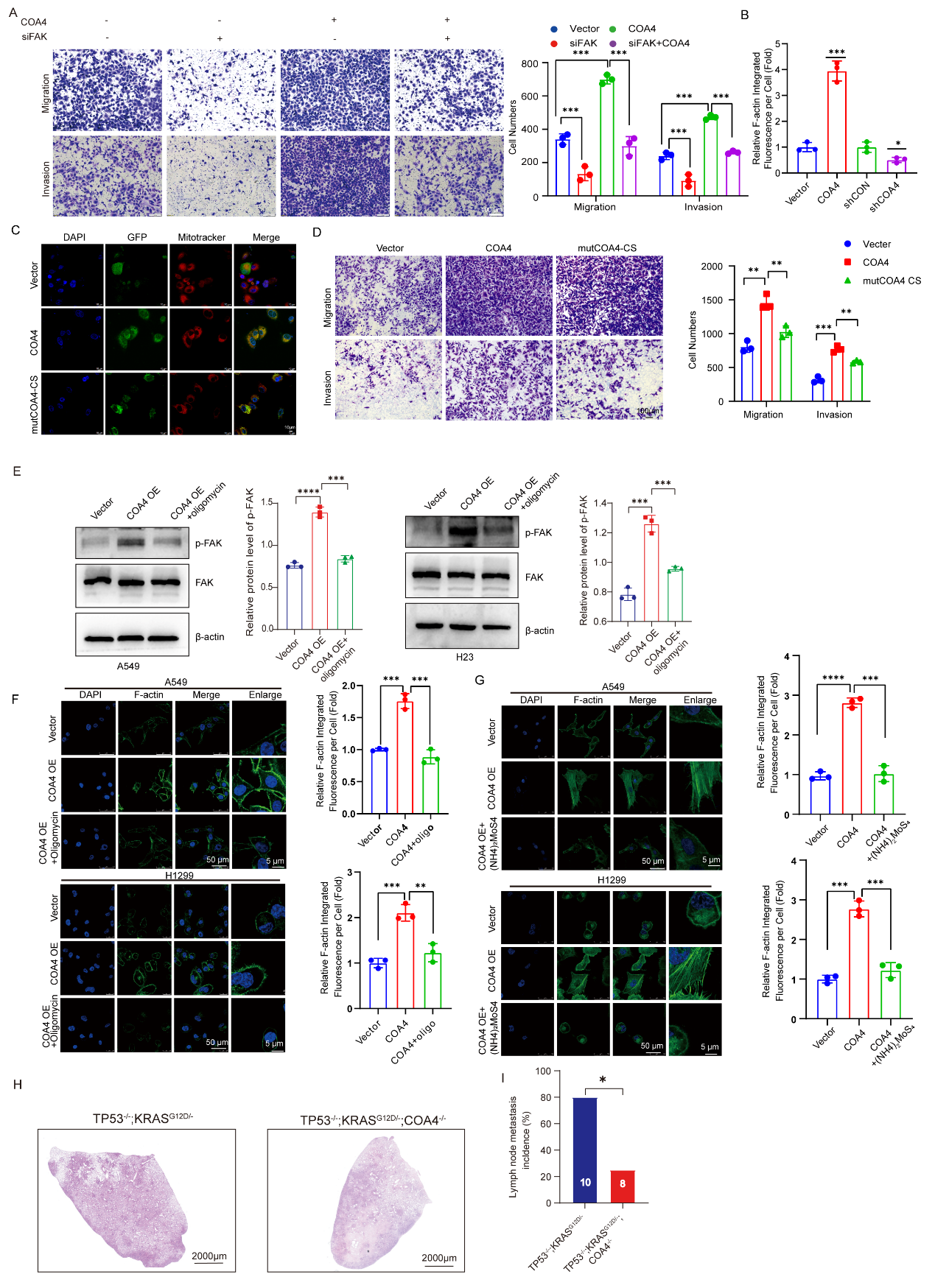


**Figure S6 COA4 promotes metastatic progression in LUAD models**

A. Transwell assays assessing the impact of FAK knockdown on the migration and invasion of H23 cells overexpressing *COA4*. Scale bar: 275 μm (n = 3, ****P* < 0.001)

B. Relative fluorescence intensity of F-actin in A549 cells following *COA4* knockdown or overexpression. (n = 3; **P* < 0.05; ****P* < 0.001)

C. Mitochondrial localization of GFP-tagged cysteine-to-serine mutant *COA4* (*mutCOA4*-CS) and wild-type *COA4* (WT-*COA4*) in A549 cells, assessed by IF co-staining with a mitochondrial marker. Scale bar: 50 μm (n = 3)

D. Invasion and migration capacities of A549 cells overexpressing GFP-WT-COA4) or mutCOA4-CS, assessed by Transwell assays. Scale bar: 275 μm (n = 3, ***P* < 0.01; ****P* < 0.001)
E. Western blot analysis of FAK and p-FAK levels in A549 and H23 cells overexpressing *COA4* and treated with oligomycin. (n = 3, ****P* < 0.001; ****P < 0.0001)
F-G. IF analysis of F-actin in *COA4*-overexpressing A549 and H1299 cells treated with oligomycin (F) and (NH₄)₂MoS₄ (G), respectively. Scale bar: 50 μm (n = 3, ***P* < 0.01; ****P* < 0.001; ****P < 0.0001)

H. Histopathological analysis of lung tissues from *TP53^-/-^;KRAS^G12D/-^;COA4^-/-^* and control *TP53^-/-^;KRAS^G12D/-^* mice by H&E staining. Scale bar: 2000 μm.

I. Quantification of lymph node metastasis incidence in*TP53^-/-^;KRAS^G12D/-^;COA4^-/-^* versus *TP53^-/-^;KRAS^G12D/-^* LUAD mice. (n = 10 and n = 8 mice per group, respectively). **P* < 0.05

The data are given as mean ± SD and compared by Student’s t test (A-B, D-G) and Chi-square test (I). . Significance levels are indicated as follows: ns, not significant; **P* < 0.05; ***P* < 0.01; ****P* < 0.001; *****P* < 0.0001.

Figure S7

**
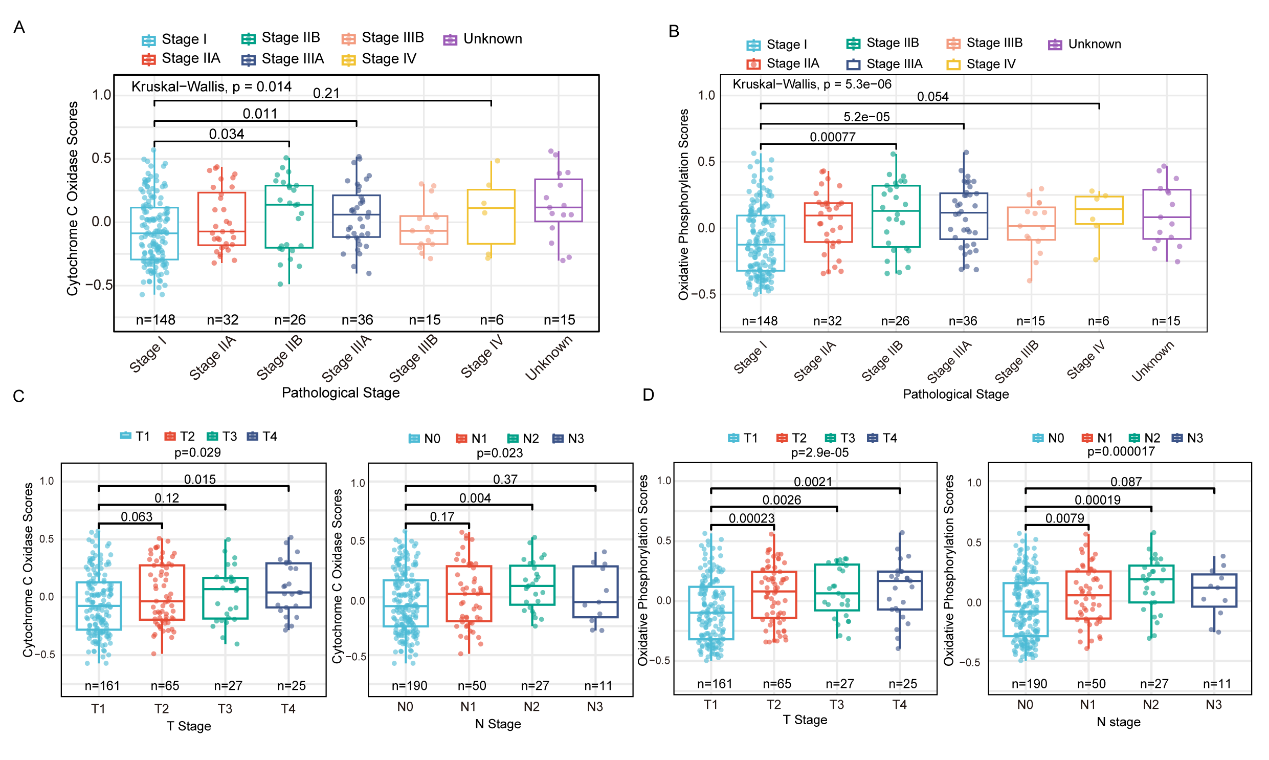
**

**Figure S7 High mitochondrial COX/OXPHOS activity correlates with aggressive progression in lung adenocarcinoma**
A-D. Gene Set Variation Analysis (GSVA) of the GSE30219 LUAD dataset to score OXPHOS and COX activity, with comparisons of COX scores (A) and OXPHOS scores (B) across different pathological stages, as well as COX scores (C) and OXPHOS scores (D) across different T and N stages.
The data are given as mean ± SD and compared by one-way ANOVA (A-D). Significance levels are indicated as follows: ns, not significant; **P* < 0.05; ***P* < 0.01; ****P* < 0.001; *****P* < 0.0001.

Figure S8


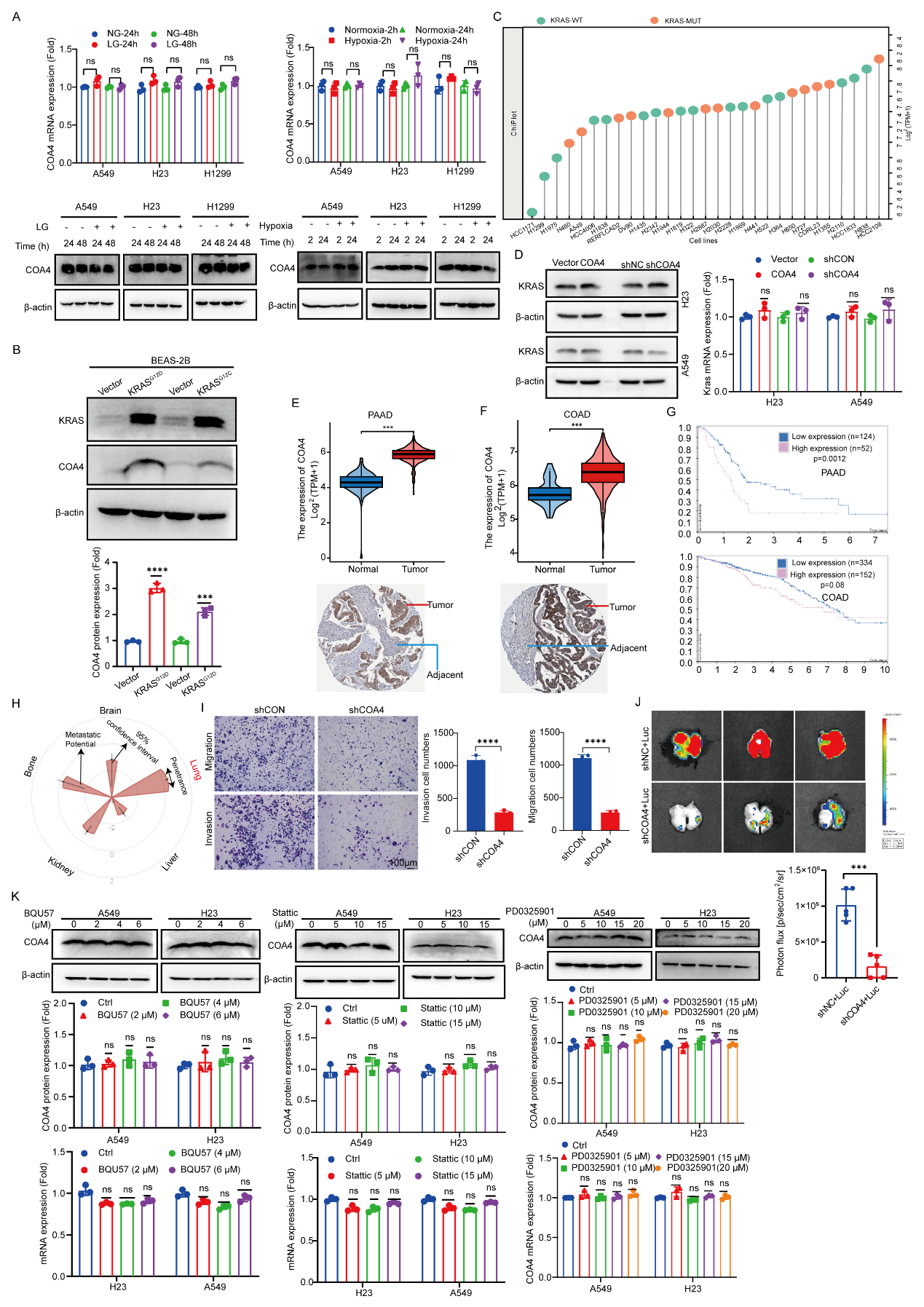


**Figure S8 Mutant *KRAS* promotes *COA4* expression via PI3K signaling axis**

A. Western blot and RT-qPCR analyses of COA4 expression levels in A549 and H23 cells under hypoxic and glucose-deprived conditions. (n = 3, ns, not significant )
B. Western blot analysis of COA4 expression in BEAS-2B cells overexpressing *KRAS*^G12C^ and *KRAS*^G12D^. (n = 3, ***P < 0.001; *****P* < 0.0001)
C. Analysis of *COA4* expression in *KRAS*-mutant, *HRAS*-mutant, and wild-type cell lines based on DepMap data.
D. Western blot analysis of KRAS protein levels in A549 and H23 cells with *COA4* knockdown or overexpression. (n = 3, ns, not significant )
E-F. *COA4* expression in PAAD (E) and COAD (F) analyzed using the Xiantao Academic Database (upper panels) and The Human Protein Atlas website (lower panels). ****P* < 0.001
G. Survival analysis of *COA4* in PAAD and COAD.
H. Analysis of the metastatic potential of ASPC1 cells across different organs using MetMap.
I. Transwell migration assay analysis of ASPC1 cells following *COA4* knockdown. Scale bar: 275 μm (n = 3, *****P* < 0.0001)
J. In vivo extravasation assessment in mouse lungs four days after tail vein injection of luciferase-tagged ASPC1 cells with *COA4* knockdown (n = 5 per group, ****P* < 0.001).
K. Western blot and RT-qPCR analyses of COA4 protein and mRNA levels in A549 and H23 cells treated with the GTPase Ral inhibitor BQU57(2–6 μM), STAT3 inhibitor Stattic (5–15 μM) and MEK inhibitor PD0325901(5–20 μM) for 48h. (n = 3, ns, not significant)
The data are given as mean ± SD and compared by Student’s t test (A-F, I-K).The Log-rank (Mantel–Cox) test was used for survival analyses (G). Significance levels are indicated as follows: ns, not significant; **P* < 0.05; ***P* < 0.01; ****P* < 0.001; *****P* < 0.0001.

Figure S9


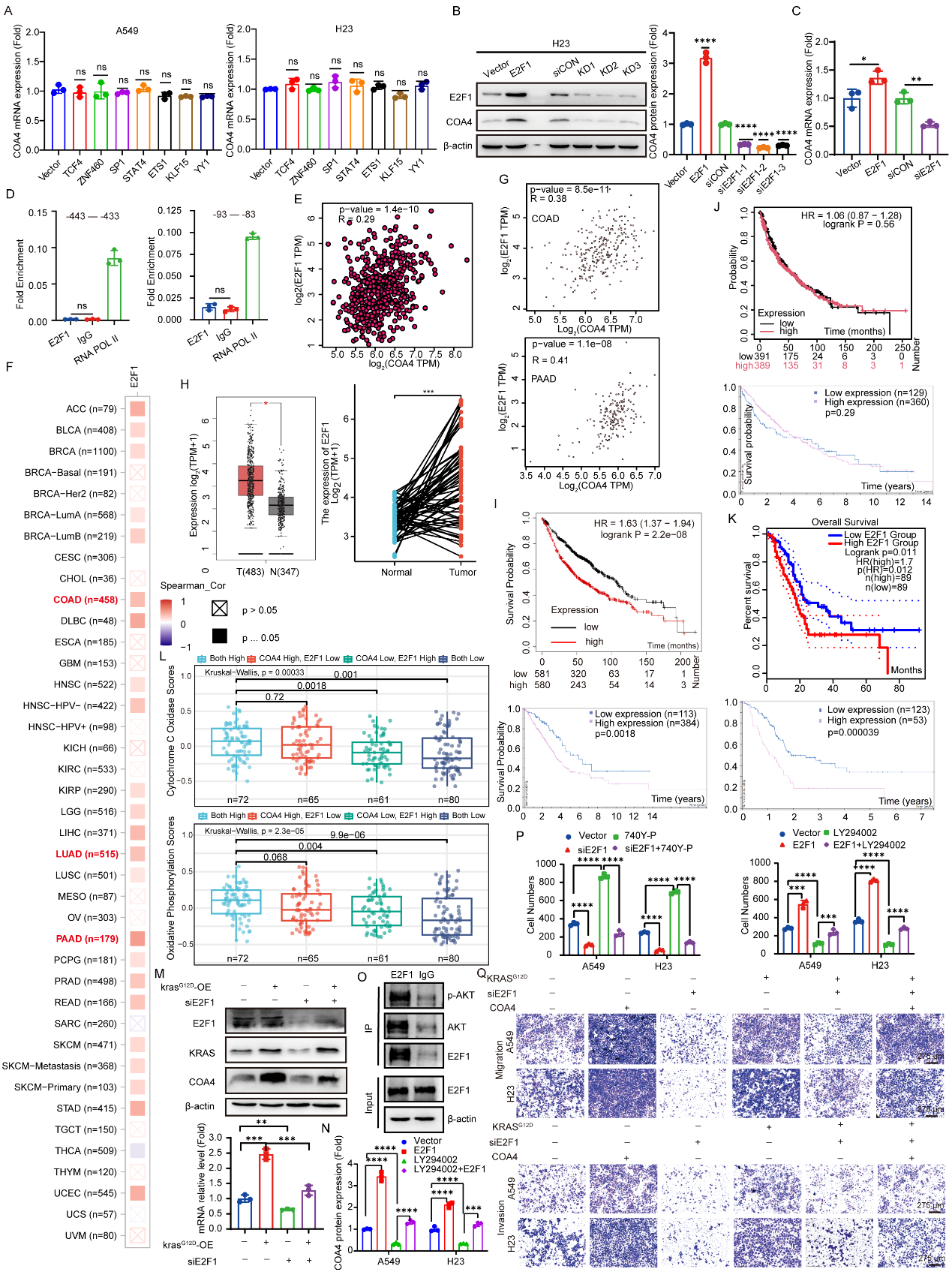


**Figure S9 E2F1 directly binds to *COA4* promoter and promotes its expression under *KRAS* mutation**

A. Predicted potential transcription factors for *COA4* identified using the JASPAR and ChIP-Atlas databases, validated by RT-qPCR. (n = 3, ns, not significant )
B-C. Western blot (B) and RT-qPCR (C) analyses of *COA4* expression in H23 cells following *E2F1* overexpression and knockdown. (n = 3, **P* < 0.05; ***P* < 0.01; *****P* < 0.0001)
D. ChIP-qPCR validation of E2F1 binding regions on the *COA4* promoter in A549 cells. (n = 3, ns, not significant )
E. Correlation analysis of *COA4* and *E2F1* expression in LUAD using the Xiantao Academic Database.
F. Analysis of the correlation between *E2F1* and *COA4* expression in various tumors using the TIMER2.0 website.
G. Correlation analysis of *COA4* and *E2F1* expression in COAD (top) and PAAD (bottom) using the Xiantao Academic Database.
H. Analysis of *E2F1* expression in LUAD using GEPIA2 (left) and the Xiantao Academic Database (right).**P* < 0.05; ****P* < 0.001.
I. Kaplan-Meier Plotter (top) and The Human Protein Atlas (bottom) were used to analyze the association between *E2F1* mRNA and protein expression levels and LUAD patient survival outcomes.
J. Kaplan-Meier Plotter (top) and The Human Protein Atlas (bottom) were used to assess the relationship between *E2F1* mRNA and protein expression levels and survival outcomes in LUSC patients.
K. GEPIA2 (top) and The Human Protein Atlas (bottom) were employed to evaluate the prognostic significance of *E2F1* mRNA and protein expression levels in PAAD.
L. R software was used to analyze the GSE30219 LUAD dataset to examine the correlation between the combined expression of *E2F1* and *COA4* and the OXPHOS and COX scores.
M. Western blot (top) and RT-qPCR (bottom) analyses of COA4 expression in *KRAS*^G12D^-overexpressing A549 cells following *E2F1* knockdown. (n = 3, ***P* < 0.01; ****P* < 0.001).
N. Western blot analysis of COA4 expression in A549 and H23 cells treated with LY294002 after *E2F1* overexpression. (n = 3; ****P* < 0.001; *****P* < 0.0001)

O. Co-IP analysis in A549 cells demonstrating specific interactions between E2F1, phospho-AKT, and total AKT. (n = 3)
P. Analysis of migration ability in A549 and H23 cells treated with 740Y-P following E2F1 knockdown (left), and with LY294002 following *E2F1* overexpression (right). (n=3; ****P* < 0.001; *****P* < 0.0001)
Q. Transwell assays evaluating migration and invasion in A549 and H23 cells following KRAS^G12D^ overexpression, *E2F1* knockdown, and *COA4* overexpression.Scale bar: 275 μm (n = 3)
The data are presented as mean ± SD and compared using Student’s t test (A–D, H, M–Q), one-way ANOVA (L), and Spearman’s rank correlation analysis (E–G). The Log-rank (Mantel–Cox) test was used for survival analyses (I–K). Significance levels are indicated as follows: ns, not significant; **P* < 0.05; ***P* < 0.01; ****P* < 0.001; *****P* < 0.0001.

Figure S10


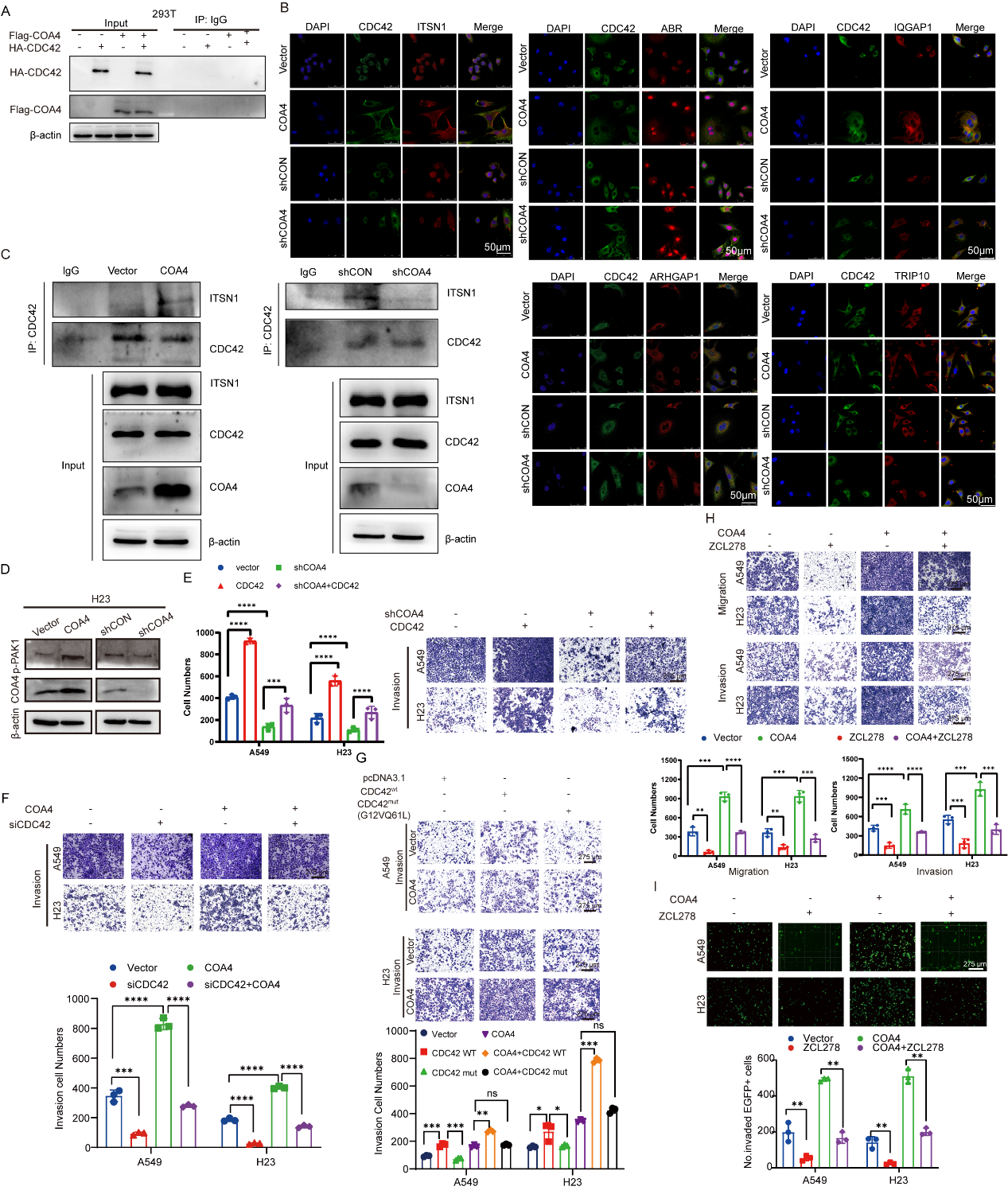


**Figure S10 COA4 interacts with and activates CDC42 to drive lung tumorigenesis**

A. Exogenous Co-IP in 293T cells validating the binding between COA4 and CDC42.

B. IF analysis demonstrating altered binding between CDC42 and its activity-regulating proteins (GEFs/GAPs) upon COA4 knockdown or overexpression. Scale bars: 50 μm. (n = 3).

C. Co-IP assays validating the effect of COA4 knockdown or overexpression on CDC42 interactions with ITSN1. (n = 3).

D. Western blot analysis of phosphorylated PAK1 (p-PAK1) expression in H23 cells with *COA4* knockdown or overexpression. (n = 3)
E-F. Transwell invasion assays evaluating: (E) the invasion of *COA4*-knockdown A549 and H23 cells after *CDC42* overexpression, and (F) the invasion of *COA4*-overexpressing cells following *CDC42* knockdown. Scale bar: 275 μm (n = 3, ****P* < 0.001; *****P* < 0.0001).
G. Transwell assays assessing the invasion ability of *COA4*-overexpressing A549 and H23 cells transfected with pcDNA3.1 (control), wild-type *CDC42* (*CDC42*^wt^), or the inactive mutant *CDC42*^G12V/Q61L^. Scale bar: 275 μm (n=3, ns, not significant; **P* < 0.05; ***P* < 0.01; ****P* < 0.001).
H. Transwell assays analyzing the migration and invasion capabilities of *COA4*-overexpressing A549 and H23 cells treated with the CDC42 inhibitor ZCL278. Scale bar: 275 μm (n = 3, ***P* < 0.01; ****P* < 0.001; *****P* < 0.0001).
I. Trans-endothelial migration assays assessing the ability of *COA4*-overexpressing A549 and H23 cells treated with ZCL278 to traverse HUVEC monolayers. Scale bar: 275 μm (n = 3, ***P* < 0.01).

The data are given as mean ± SD and compared by Student’s t test (D–I). Significance levels are indicated as follows: ns, not significant; **P* < 0.05; ***P* < 0.01; ****P* < 0.001; *****P* < 0.0001.

Figure S11


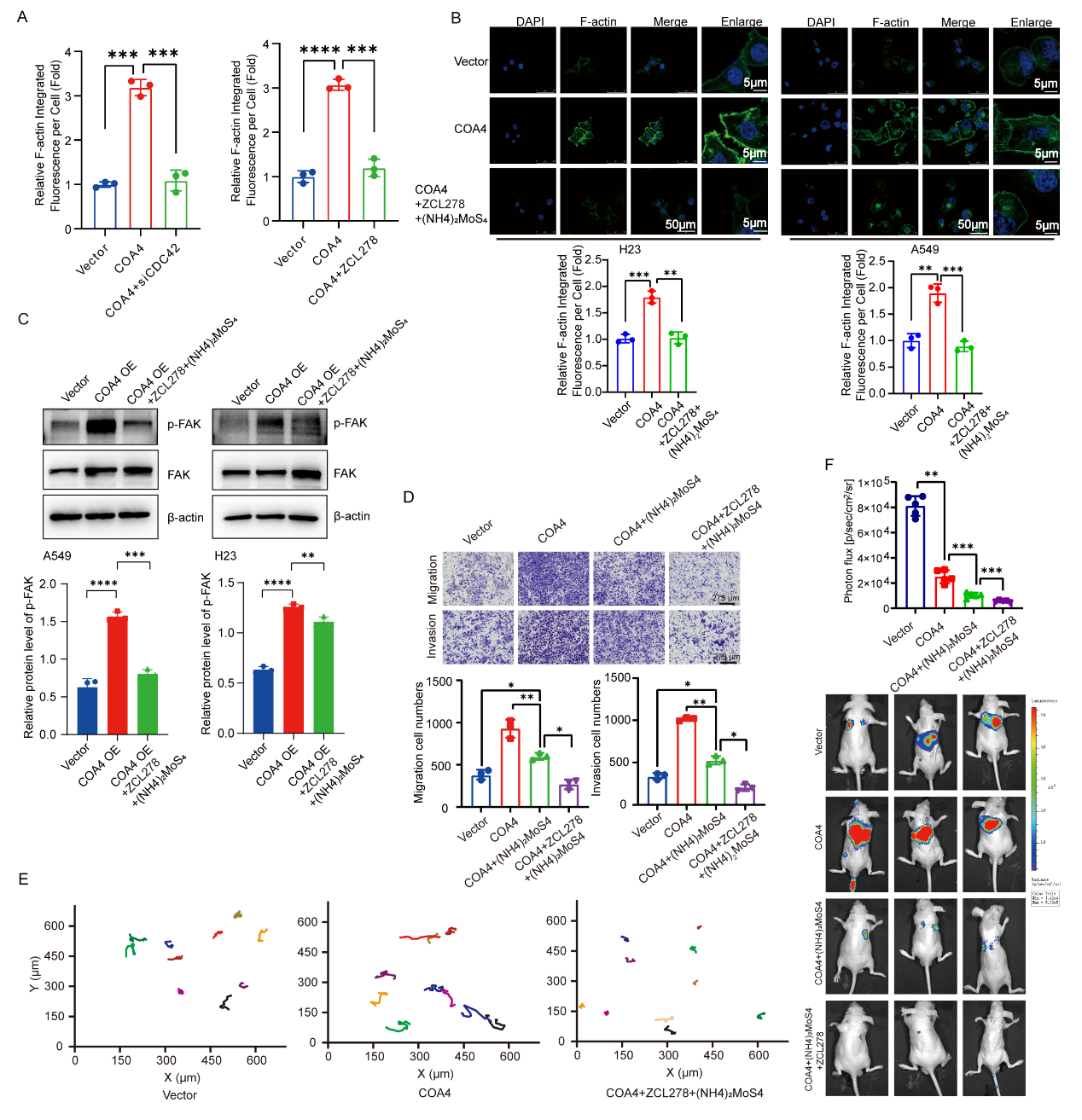


**Figure S11 *COA4* modulates lung tumorigenesis through CDC42 signaling.**
A. Quantification of F-actin fluorescence intensity in filopodia-like structures of *COA4*-overexpressing A549 cells upon CDC42 knockdown or treatment with CDC42 inhibitor ZCL278. (n=3, ****P* < 0.001; *****P* < 0.0001)
B. IF analysis of F-actin filopodia-like structures in *COA4*-overexpressing A549 and H23 cells treated with ZCL278 and (NH_4_)₂MoS₄. Scale bar: 275 μm (n=3, ***P* < 0.01; ****P* < 0.001)

C. Western blot analysis of FAK and p-FAK levels in *COA4*-overexpressing A549 and H23 cells treated with ZCL278 and (NH_4_)₂MoS₄. (n=3, ***P* < 0.01; ****P* < 0.001; *****P* < 0.0001)

D. Transwell assays evaluating the migration and invasion abilities of *COA4*-overexpressing A549 cells following treatment with ZCL278 and (NH_4_)₂MoS₄. Scale bar: 275 μm(n=3, **P* < 0.05; ***P* < 0.01)
E. Single-cell time-lapse imaging of *COA4*-overexpressing A549 cells treated with ZCL278 and (NH_4_)₂MoS₄, with corresponding movement trajectory plots. (n=3)
F. In vivo metastasis analysis via tail vein injection of luciferase-labeled *COA4*-overexpressing A549 cells, followed by intraperitoneal treatment with ZCL278 and (NH_4_)₂MoS₄. Metastatic burden was assessed by measuring fluorescence intensity (n = 5 per group, ***P* < 0.01; ****P* < 0.001)
The data are given as mean ± SD and compared by Student’s t test (A–D, F). Significance levels are indicated as follows: ns, not significant; **P* < 0.05; ***P* < 0.01; ****P* < 0.001; *****P* < 0.0001.

Figure S12

**
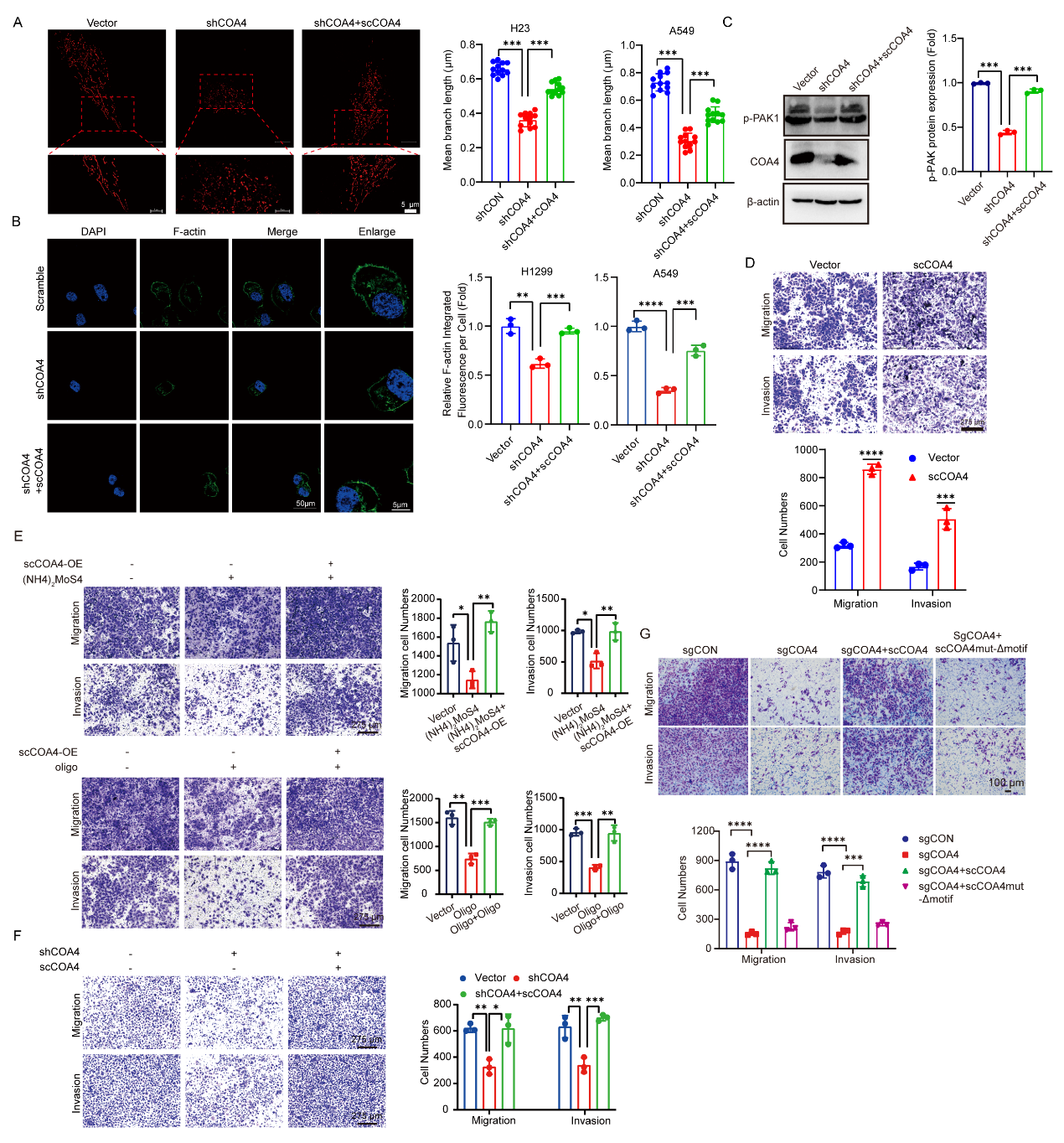
**

**Figure S12 *Saccharomyces cerevisiae*-derived COA4 has a cancer-promoting function in LUAD cells**

A. Super-resolution structured illumination microscopy (SIM) of mitochondria in *COA4*-knockdown H23 cells rescued by *scCOA4* re-expression (Left). Quantitative analysis of mitochondrial fragmentation in in *COA4*-knockdown H23 and A549 cells rescued by *scCOA4* re-expression (right). Scale bar: 5 μm (n=12 cells each, ****P* < 0.001).
B. IF analysis of F-actin-enriched filopodia-like structures in *COA4*-knockdown H1299 cells rescued with *scCOA4* (left); Quantification of filopodia-like structures in *COA4*-knockdown H1299 and A549 cells following *scCOA4* re-expression (right). Scale bar: 50 μm,(n=3, ***P* < 0.01; ****P* < 0.001; *****P* < 0.0001)
C. Western blot analysis of p-PAK1 levels in COA4-knockdown H23 cells rescued with *scCOA4*. (n=3, ****P* < 0.001)
D. Transwell assays evaluating the migration and invasion abilities of H23 cells transfected with *scCOA4*. Scale bar: 275 μm (n=3, ****P* < 0.001; *****P* < 0.0001)
E. Transwell assays analyzing the migration and invasion capabilities of A549 cells transfected with *scCOA4* and subsequently treated with (NH_4_)₂MoS₄ (top) or oligomycin (bottom). Scale bar: 275 μm (n=3, **P* < 0.05; ***P* < 0.01; ****P* < 0.001)
F. Transwell assays assessing the migration and invasion abilities of *COA4*-knockdown ASPC1 cells rescued by *scCOA4* re-expression. Scale bar: 275 μm (n=3, **P* < 0.05; ***P* < 0.01; ****P* < 0.001)

G. Transwell analysis of metastatic potential in human lung adenocarcinoma cells following COA4 knockdown and rescue with motif-mutated scCOA4 (scCOA4mut-Δmotif). Scale bar: 275 μm (n=3, ****P* < 0.001;*****P* < 0.0001)
The data are given as mean ± SD and compared by Student’s t test (A-G). Significance levels are indicated as follows: ns, not significant; *P < 0.05; **P < 0.01; ****P* < 0.001; *****P* < 0.0001.
